# Computational identification and evaluation of *Adenium obesum* phytochemicals as potential EGFR inhibitors for targeted therapy against non-small-cell lung cancer

**DOI:** 10.1371/journal.pone.0357096

**Published:** 2026-08-31

**Authors:** Md. Naziur Rahman, Abu Yousuf Hossin, Shahriar Mannan Imon Talukder, Mt. Sumaiya Siddika, Md. Harun-Ur-Rashid, Shahanaz Parveen, Md. Ridwanul Islam, Md. Yeamin Hossain, Mahfuj Ahmed, Brototi Chakraborty Dyuti, Zarin Tabassum, Ajoy Kumer

**Affiliations:** 1 College of Agricultural Sciences, IUBAT—International University of Business Agriculture and Technology, Dhaka, Bangladesh; 2 Barind Medical College & Hospital, Rajshahi, Bangladesh; 3 Department of Genetics and Plant Breeding, Faculty of Agriculture, Sher-e-Bangla Agricultural University, Dhaka, Bangladesh; 4 Department of General Medicine, Saveetha Medical College and Hospitals, Saveetha Institute of Medical and Technical Sciences, Chennai, India; 5 Department of Chemistry, College of Arts and Sciences, IUBAT—International University of Business Agriculture and Technology, Dhaka, Bangladesh; Bai Jerbai Wadia Hospital for Children, INDIA

## Abstract

Non-small-cell lung cancer (NSCLC) remains the leading cause of lung cancer–related mortality, largely driven by aberrant activation of the epidermal growth factor receptor (EGFR). Despite the clinical success of EGFR tyrosine kinase inhibitors (TKIs), intrinsic and acquired resistance, coupled with safety concerns, highlight the need for novel, safer inhibitors. Natural products represent an underexplored source of structurally diverse bioactive compounds with favorable biocompatibility. In this study, a comprehensive in silico approach is used to evaluate phytochemicals from *Adenium obesum* as potential candidate EGFR-targeting compound. Initially, sixteen phytochemicals were first assessed for predicted antineoplastic activity using PASS. High-scoring molecules were docked against the EGFR kinase domain (PDB ID: 1M17), besides performed detailed protein–ligand interaction analysis, drug-likeness and ADMET profiling, toxicity prediction and 100-ns molecular dynamics (MD) simulations. PASS-based bioactivity prediction revealed strong anticancer potential among the sixteen screened compounds, with consistently high antineoplastic and antiproliferative activity probabilities (Pa > 0.79) and low inactivity scores, supporting their selection for subsequent docking, ADMET, and molecular dynamics analyses. Next, several phytochemicals exhibited strong docking affinities, with Cardenolide achieving the highest binding score (–9.9 kcal/mol) and forming stable interactions with key catalytic residues. A 100-ns MD simulation confirmed the structural stability, persistent binding, and dynamic integrity of the EGFR–Cardenolide complex under physiological conditions. Importantly, interaction mapping revealed that Cardenolide engages conserved and functionally critical regions of the EGFR kinase domain associated with catalytic activity and structural stability, supporting its mechanistic relevance as an ATP-competitive scaffold. Additionally, predicted pharmacokinetic and toxicity profiles further supported Cardenolide’s suitability as a drug-like candidate. Collectively, these results identify Cardenolide as a computationally prioritized candidate with favorable predicted EGFR-binding characteristics, structural stability, and physicochemical and toxicity profiles. However, as the present study is based entirely on computational analyses, these findings should be considered hypothesis-generating and do not establish EGFR inhibitory activity or therapeutic efficacy. Experimental validation, including biochemical kinase inhibition and cellular assays, is therefore required to determine the actual EGFR inhibitory potential and anticancer activity of Cardenolide. Nevertheless, the findings provide a rational basis for prioritizing Cardenolide for further experimental investigation and illustrate the potential of Adenium obesum phytochemicals as a source of candidate EGFR-targeting compounds for future NSCLC drug discovery.

## Introduction

Lung cancer remains one of the most pervasive and lethal malignancies worldwide, continuing to impose an overwhelming public health burden despite advances in diagnosis and treatment [1]. Recent global estimates indicate more than 2.40 million incident cases and 1.80 million deaths in 2022, underscoring its position as the leading cause of cancer mortality across regions [2]. Among the two major pathological classes of lung cancer, small-cell lung cancer (SCLC) and non-small-cell lung cancer (NSCLC) the latter accounts for approximately 85% of all cases and is frequently diagnosed at advanced stages [3,4]. More than 70% of cases are identified at stage III or IV, leaving limited therapeutic windows and contributing to poor prognosis [5].

The molecular landscape of NSCLC is shaped by diverse genetic alterations involving EGFR, KRAS, HER2, ALK, MET, PIK3CA, and several other oncogenes [6,7]. Among these, activating mutations in the epidermal growth factor receptor (EGFR) plays a pivotal role in driving tumorigenesis through dysregulated cell proliferation, survival, angiogenesis, and metastasis. Somatic EGFR mutations occur in 15–20% of lung adenocarcinomas, with pronounced variation across demographic groups [8]. Two mutations exon 19 deletions (Del19) and the L858R substitution in exon 21 comprise nearly 90% of EGFR-mutated NSCLC cases and are strongly associated with sensitivity to EGFR-targeted therapy. Therefore, targeted inhibition of EGFR has transformed the clinical management of NSCLC [9,10]. First- and second-generation EGFR tyrosine kinase inhibitors (TKIs) include gefitinib, erlotinib, afatinib, and dacomitinib prolong progression-free survival and improve quality of life compared with conventional platinum-based chemotherapy [11,12]. The emergence of the third-generation TKI osimertinib, with superior efficacy and tolerability, further revolutionized frontline therapy [13]. Despite these advances, intrinsic and acquired drug resistance remains a formidable barrier. Resistance typically arises after therapy and may involve secondary EGFR mutations (e.g., T790M, C797S), bypass signaling through MET amplification or KRAS activation, histological transformation, or engagement of alternative pro-survival pathways [14,15]. These limitations underscore the urgency of identifying novel, mechanistically distinct EGFR inhibitors capable of overcoming resistance while maintaining favorable safety profiles.

**Fig 1:**
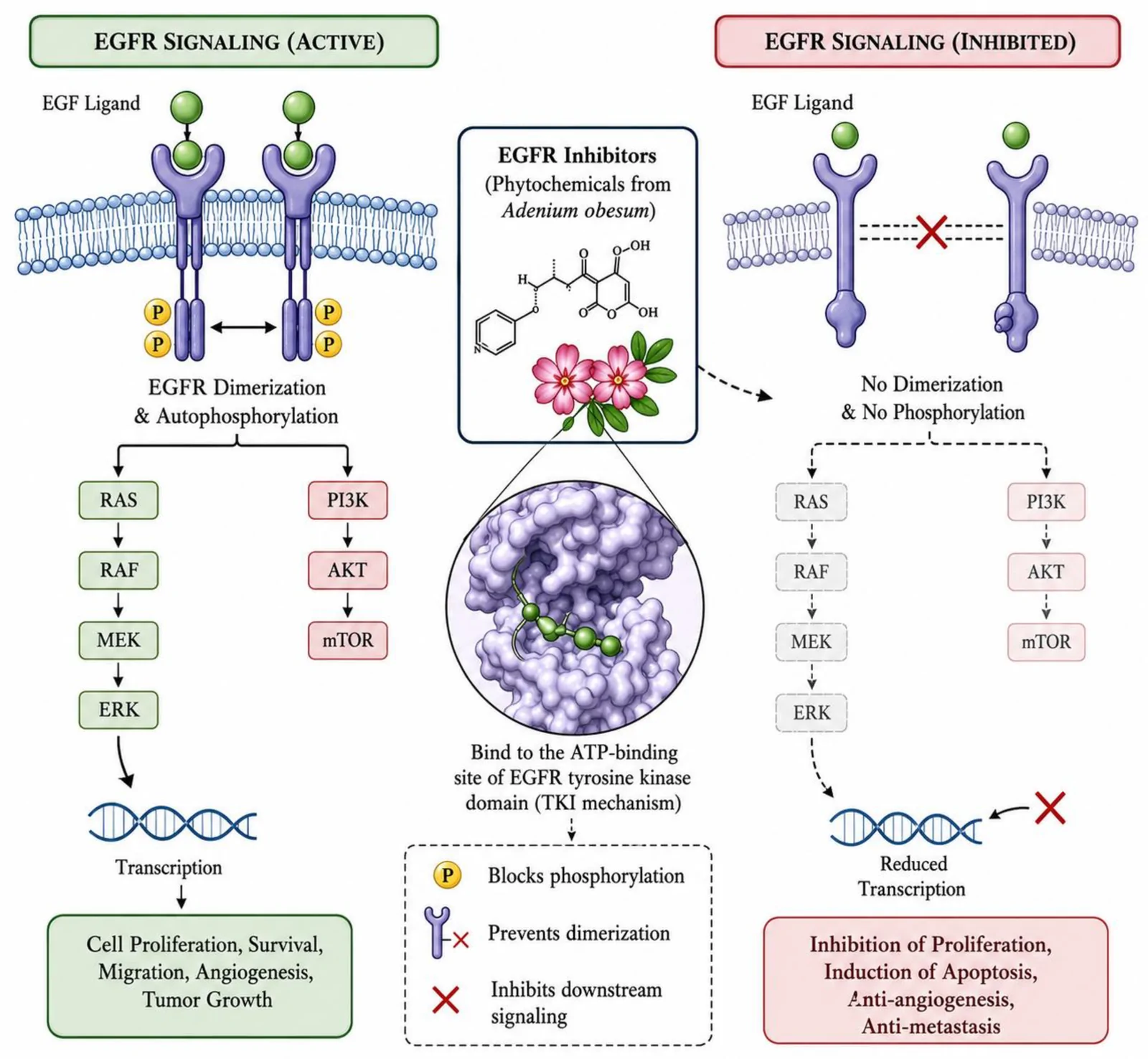
Schematic illustration of epidermal growth factor receptor signaling and its inhibition in non-small-cell lung cancer.

To provide a mechanistic overview of EGFR-driven tumor progression and therapeutic intervention in NSCLC, a schematic representation is presented in Fig 1. The illustration summarizes EGFR activation and its downstream oncogenic signaling pathways, including RAS/RAF/MEK/ERK and PI3K/AKT/mTOR, which contribute to NSCLC progression. The schematic also illustrates how EGFR kinase-domain inhibition may suppress these pathways, providing the mechanistic rationale for investigating phytochemicals as potential EGFR-targeting agents.

Natural products have re-emerged as a compelling reservoir for anticancer drug discovery due to their structural diversity, biocompatibility, and historically validated therapeutic relevance [16–18]. Many plant-derived compounds exhibit potent antiproliferative, pro-apoptotic, and signaling-modulatory effects, and several have already transitioned into clinically approved anticancer agents [19,20]. However, despite growing interest, the exploration of natural products specifically as EGFR-targeted therapeutics for NSCLC remains limited, fragmented, or insufficiently validated at molecular and pharmacokinetic levels. Many medicinally important species remain underexplored, and their pharmacological potential has not yet been systematically evaluated. Identifying such plants and characterizing their biological properties is critical for expanding the pipeline of natural compounds with therapeutic relevance. In recent years, increasing attention has been directed toward traditionally used but scientifically understudied species.

*Adenium obesum* (desert rose), a succulent shrub of the Apocynaceae family, is widely recognized for its ornamental value as well as its long history of use in traditional medicine across Africa, the Arabian Peninsula, and parts of Asia [21–23]. Phytochemical investigations consistently reveal that the species is rich in diverse secondary metabolites, including cardiac glycosides, pregnanes, flavonoids, triterpenes, and various terpenoids that underpin its broad pharmacological spectrum. Previous studies have reported significant antioxidants, antibacterial, antiviral, anti-inflammatory, and anticancer activities in its leaf, flower, and stem extracts, with several bioactive compounds such as benzofuran derivatives, rosmarinic acid, hexadecanoic acid methyl ester, and ethnomedicinal cardenolides playing central roles [21,23]. Despite this promising bioactivity, scientific research on *A. obesum* remains comparatively limited, particularly for region-specific ecotypes such as those found in the Arabian Peninsula and South Asia. The growing evidence highlighting *A. obesum’s* antioxidant, antimicrobial, and cytotoxic properties provides strong justification for selecting this species as the focus of the present study [24,25]. The plant’s rich and unique phytochemical profile suggests significant potential for discovering new therapeutic agents, especially natural antioxidants and anticancer compounds [25]. Therefore, investigating *A. obesum* is not only scientifically relevant but also timely, as it offers an underexplored yet pharmacologically potent source of bioactive molecules that may contribute to the development of safer, plant-based therapeutics.

Recent breakthroughs in computer-aided drug design (CADD) including structure-based virtual screening, molecular docking, pharmacophore modeling, ADMET profiling, bioactivity scoring, and molecular dynamics (MD) simulations offer unprecedented opportunities to accelerate natural-compound-based drug discovery [26]. These techniques enable rapid assessment of binding affinities, drug-likeness, functional stability, and interaction dynamics within protein–ligand complexes, thereby reducing reliance on costly and time-consuming in vitro or in vivo assays. Advances in artificial intelligence and data-driven drug discovery further support the development of novel therapeutics [27]. Beyond molecular docking and dynamic stability analyses, integrating evolutionary conservation and cancer bioinformatics provides an important framework for validating the biological relevance of predicted drug–target interactions [28]. Residues located within highly conserved regions of oncogenic proteins are often functionally indispensable and less tolerant to mutational disruption, making them attractive to therapeutic targets. Therefore, combining structural bioinformatics, conservation mapping, mutational landscape analysis, and clinical expression profiling can strengthen the translational significance of computationally identified inhibitors and provide mechanistic insight into their therapeutic potential [29].

In this context, the phytochemical diversity of Adenium obesum, a medicinally significant species, presents a promising but underexplored source of candidate compounds for EGFR-targeted drug discovery. Although previous literature highlights the anticancer potential of various natural compounds, including alkaloids such as lochnericine and phytoconstituents from species such as Catharanthus roseus, few studies have conducted a rigorous, multi-tier computational evaluation of A. obesum phytochemicals in the context of EGFR targeting in NSCLC [30]. Moreover, existing studies have seldom integrated molecular docking, comprehensive ADMET assessment, bioactivity prediction, and long-timescale molecular dynamics simulations to systematically prioritize candidate compounds.

To address these gaps, the present study employs a systematic in silico pipeline to identify and characterize putative EGFR-binding candidates from A. obesum. The workflow integrates molecular docking, comparison with established EGFR inhibitors, physicochemical and ADMET profiling, bioactivity prediction, and molecular dynamics simulations to evaluate the predicted binding characteristics and structural stability of selected protein–ligand complexes. Rather than establishing experimentally confirmed inhibitory activity, this computational framework is intended to prioritize phytochemicals according to their predicted EGFR-binding potential, physicochemical properties, pharmacokinetic characteristics, and molecular stability. The findings are therefore hypothesis-generating and provide a basis for selecting candidate compounds for subsequent biochemical and cellular validation. By combining phytochemical screening with complementary computational approaches, this study aims to identify candidate EGFR-targeting compounds from A. obesum that warrant further experimental investigation in the context of NSCLC drug discovery. To the best of our knowledge, this work represents the first systematic in silico assessment of A. obesum phytochemicals against EGFR, providing a computational foundation for the experimental evaluation of these compounds as potential EGFR-targeting candidates.

## Materials and methods

**Fig 2:**
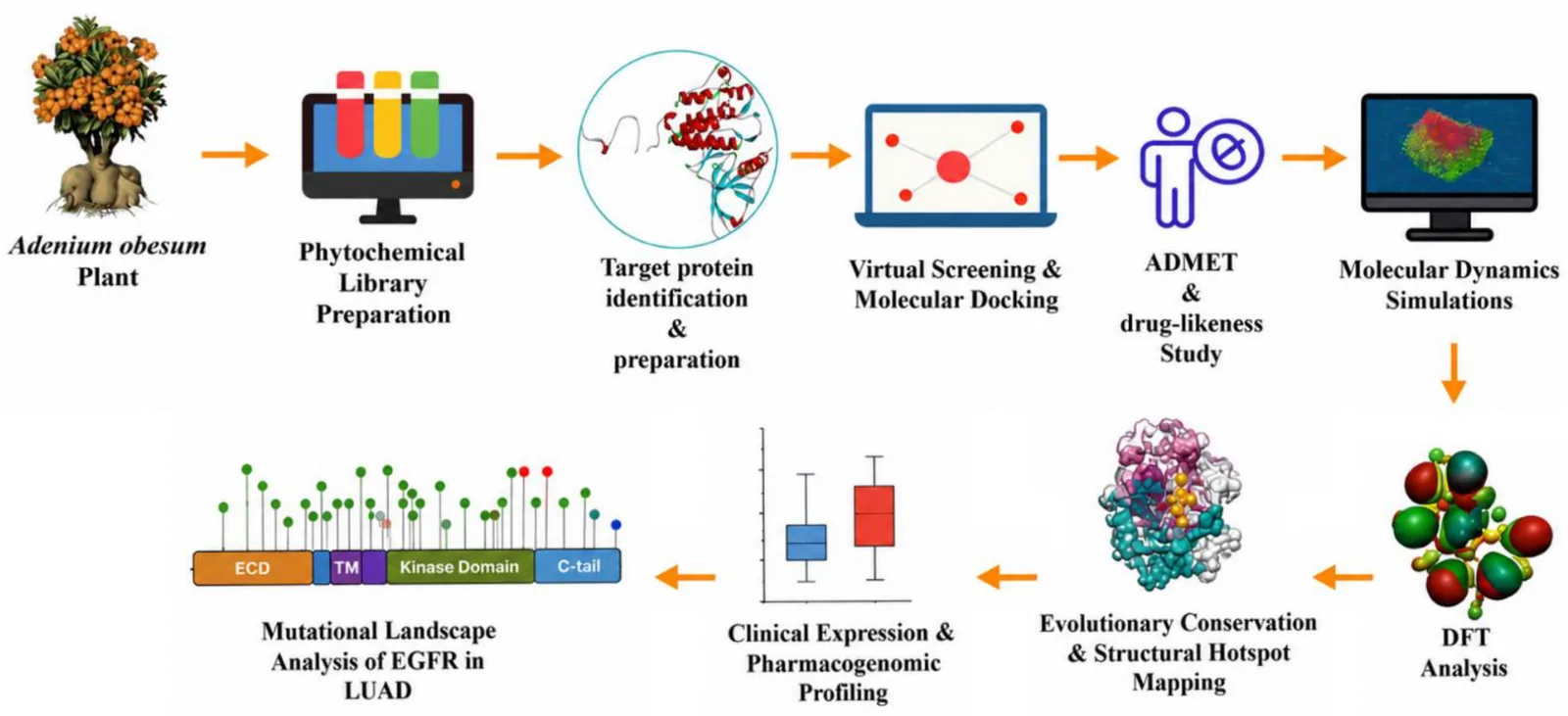
Stepwise flow diagram of the methods followed in the study.

This study employs a comprehensive *in silico* approach, integrating virtual screening and advanced molecular dynamics simulation techniques to explore the potential of anticancer plant-derived phytochemicals for EGFR-targeted lung cancer therapy. The overall systematic workflow is presented in the flowchart shown in Fig 2.

### Preparation of receptor proteins

The three-dimensional crystal structure of the human Epidermal Growth Factor Receptor (EGFR) tyrosine kinase domain was retrieved from the Protein Data Bank (PDB; https://www.rcsb.org/) using PDB ID 1M17 on 15 November 2025. In addition, two experimentally determined EGFR structures carrying clinically relevant mutation combinations were retrieved from the PDB for comparative molecular docking analysis: PDB ID 4I22, representing the L858R/T790M EGFR mutant structure, and PDB ID 6LUD, representing the L858R/T790M/C797S EGFR mutant structure. These structures were used to evaluate the predicted docking characteristics of the investigated phytochemicals across wild-type and mutation-containing EGFR backgrounds. All three protein was prepared for molecular docking and simulation analyses by removing all heteroatoms, including any co-crystallized ligands and water molecules, using Discovery Studio (Studio, 2020). Hydrogen atoms were then added to the protein structure to ensure proper valency and geometry. Energy minimization of the receptor was performed using Swiss-PdbViewer (version 4.1.0) to remove any steric clashes and optimize the overall stability of the protein structure [31]. The processed protein was finally saved in PDB format and used for subsequent molecular docking and molecular dynamics simulation studies. Structural quality parameters and essential properties have been summarized in S1Table and S1 Fig in S1 File.

### Preparation of ligands

From the Indian Medicinal Plants, Phytochemistry, and Therapeutics (IMMPAT) (https://cb.imsc.res.in/imppat/) database [32], a total of 16 bioactive phytochemicals of *Adenium obesum* were collected for this study on 15 November 2025. In addition, four well-established EGFR tyrosine kinase inhibitors Osimertinib, Afatinib, Erlotinib, and Lazertinib were selected as reference (control) drugs due to their proven therapeutic efficacy in the treatment of non-small-cell lung cancer (NSCLC) [33–35]. The chemical structures of the selected *Adenium obesum* phytochemicals and reference drugs were retrieved from the PubChem database [36] in SDF format. Using PyRx (PyRx 0.8), the three-dimensional structures of all ligands were optimized and converted into PDBQT format to make them suitable for molecular docking studies. ChemDraw Pro-8 was used to generate and present the two-dimensional structures of the phytochemicals and standard drugs, which are illustrated in S2 Table in S1 File. These prepared ligand structures were subsequently used for molecular docking and molecular dynamics simulation analyses to evaluate their binding affinity and stability against the EGFR receptor.

### Prediction of Biological Activity (PASS) analysis

The PASS analysis was performed using the widely recognized PASS Online server (http://way2drug.com/PassOnline/predict.php) access on 17 November 2025. The probability of activity (Pa) and probability of inactivity (Pi) values generated by the platform were used to assess the potential biological activities of the studied compounds [37].

### Molecular docking

Molecular docking is a fundamental computational approach in drug discovery used to predict the interaction of small molecules with target proteins and to characterize their putative binding modes [38]. In the present study, molecular docking was performed using the AutoDock Vina wizard integrated into PyRx (version 0.8, accessed November 17, 2025) [39]. Docking was performed using a grid encompassing the ATP-binding region and surrounding catalytic residues to allow comprehensive exploration of ligand-binding conformations [40]. Docking was performed with an exhaustiveness value of 8 and a maximum of nine output modes per ligand. The final pose selection was based not only on the lowest predicted binding energy but also on the consistency of the binding orientation, favorable interaction geometry with key catalytic and regulatory residues, and absence of steric clashes, ensuring biologically plausible binding conformations. The docking grid box was defined using grid box parameters detailed in Table 1, ensuring coverage of the entire receptor structure. AutoDock Vina’s default scoring function was employed to estimate the binding affinities between the ligands and the EGFR tyrosine kinase domain. For each ligand, nine different binding poses were generated. Among them, the top-ranked pose exhibiting the lowest binding energy and a root-mean-square deviation (RMSD) value of 0.000 Å for both lower and upper bounds was selected for further analysis. While AutoDock Vina provides efficient and reliable estimations of ligand–receptor binding affinity, it does not fully account for protein flexibility or solvent effects. The protein–ligand interaction patterns and binding conformations were visualized and analyzed using Discovery Studio 2024 (version 24.1.0.23298, accessed November 18, 2025).

**Table 1:** Grid box parameters used for PyRx docking analysis in this study.

EGFR Structure	PDB ID	Grid Box Center (Å)	Grid Box Dimensions (Å)
Wild-type EGFR	**1M17**	X = 23.5057 Y = 9.8472 Z = 59.8229	X = 93.8229 Y = 66.4900 Z = 51.5337
EGFR L858R/T790M	**4I22**	X = 15.7717 Y = −6.1299 Z = 12.1764	X = 62.2463 Y = 41.7308 Z = 59.0858
EGFR L858R/T790M/C797S	**6LUD**	X = −57.6458 Y = −7.7344 Z = −24.4860	X = 45.5126 Y = 62.8431 Z = 60.7468

To investigate whether clinically relevant EGFR mutations were associated with changes in the predicted docking characteristics of the investigated phytochemicals, comparative molecular docking was additionally performed against two experimentally determined mutation-containing EGFR structures. PDB ID 4I22 [41], representing an EGFR structure carrying the L858R and T790M mutations, and PDB ID 6LUD [42], representing an EGFR structure carrying the L858R, T790M, and C797S mutations, were included for comparative analysis. All 16 *Adenium obesum* phytochemicals investigated in the present study were docked against the wild-type EGFR structure (1M17) and the two mutation-containing structures (4I22 and 6LUD) using the same docking protocol and parameter settings described above. The resulting docking scores were compared across the three structural backgrounds to examine compound-dependent differences in predicted docking behavior.

### Docking protocol validation

To assess the reliability and reproducibility of the molecular docking methodology, a re-docking validation procedure was performed using the co-crystallized native ligand present in the EGFR crystal structure (PDB ID: 1M17). The crystallographic ligand was extracted from the receptor complex and subsequently prepared using the same protocol applied to the screened phytochemicals. Briefly, hydrogen atoms were added, torsional bonds were assigned, and the ligand geometry was energy minimized prior to docking analysis. The prepared native ligand was then re-docked into the ATP-binding pocket of EGFR using AutoDock Vina implemented in PyRx under the same grid coordinates (Table 1) and docking parameters employed during virtual screening. The resulting docked conformation was superimposed onto the experimentally resolved crystallographic pose using PyMOL, and the root mean square deviation (RMSD) between the two conformations was calculated to evaluate docking accuracy. An RMSD value below 2.0 Å was considered indicative of successful reproduction of the experimental binding orientation and validation of the docking protocol [43].

### Prediction of drug-likeness and absorption, distribution, metabolism, and excretion (ADMET) properties

The assessment of ADME profiles is a crucial step in pharmacological research and drug development, as it provides valuable insights into a compound’s pharmacokinetic behavior, safety, and therapeutic potential [44]. In the present study, all selected ligand compounds were subjected to comprehensive drug-likeness and ADMET analysis. The Canonical SMILES of each compound were retrieved from the PubChem database and used as input in SwissADME (http://www.swissadme.ch, accessed November 18, 2025) to predict key physicochemical and pharmacokinetic parameters. These included molecular weight, hydrogen bond donors and acceptors, lipophilicity (iLOGP), water solubility (Log S, ESOL), gastrointestinal absorption, bioavailability score, and compliance with Lipinski’s Rule of Five. In addition, medicinal chemistry parameters such as synthetic accessibility were assessed to evaluate the feasibility of compound development. To further strengthen the pharmacokinetic and toxicity assessment, additional in silico tools ADMET SAR, pkCSM, and ProTox-III were employed. These tools were used to predict critical ADMET features such as human intestinal absorption, blood–brain barrier (BBB) permeability, cytochrome P450 enzyme interactions (including CYP2D6 and CYP3A4), AMES mutagenicity, skin sensitization, and general toxicity risks. The predicted ADMET profiles of the selected *Adenium obesum* compounds were then compared with those of the standard reference drugs to determine their relative suitability as potential drug candidates.

### Molecular Dynamics simulations

Molecular dynamics (MD) simulations were conducted to evaluate the structural stability and dynamic behavior of the docked protein–ligand complexes, serving as a critical post-docking validation step.

All simulations were performed using the Desmond MD engine (Schrödinger, LLC) on 25 November 2025, which offers high computational efficiency and scalability for biomolecular systems. The production simulations were carried out for 100 ns under an NPT ensemble, maintaining constant pressure and temperature to mimic physiological conditions. The protein–ligand complex was prepared using the Protein Preparation Wizard in Maestro (Schrödinger Suite). Missing side chains and loops were rebuilt, bond orders were corrected, and hydrogen atoms were added. The protonation states of ionizable residues and ligands were assigned at physiological pH (7.2) using Epik and PROPKA, ensuring accurate charge distribution throughout the system. The OPLS4 force field was employed to parameterize both protein and ligand, providing a consistent and reliable description of molecular interactions. The system was solved in an explicit TIP3P water model using a trucked octahedron simulation box, maintaining a 10 Å buffer distance around the complex to prevent boundary artifacts. To replicate physiological ionic strength, 0.15 M NaCl was added, and periodic boundary conditions were applied in all three dimensions. Prior to the production phase, the system underwent energy minimization followed by a multi-step equilibration process, according to Desmond’s default relaxation protocol, ensuring system stability and relaxation of steric clashes.

Following the completion of the simulation, multiple structural and dynamic parameters were analyzed using the Simulation Interaction Diagram (SID) and Maestro Analysis tools. These included roots mean square deviation (RMSD) and root mean square fluctuation (RMSF) to assess structural stability and residue flexibility, radius of gyration (Rg) to determine compactness, and total and potential energy profiles to monitor system convergence. Additionally, protein–ligand interaction profiles such as hydrogen bonds, hydrophobic interactions, π–π stacking, salt bridges, and water-mediated contacts were systematically evaluated to provide a comprehensive understanding of the conformational stability and interaction persistence of the complexes throughout the simulation trajectory.

### DFT analysis

The Jaguar module of Schrodinger Suite with the B3LYP (Becke, 3-parameter, Lee-Yang-Parr) functional and the 6-31G* basis set was used to conduct DFT calculations [45,46]. Single-point energy calculations were performed. Analysis of frontier molecular orbitals provided insights into the energies of the highest occupied molecular orbital (HOMO) and the lowest unoccupied molecular orbital (LUMO), allowing for the derivation of the energy gap and global reactivity descriptors. Molecular Electrostatic Potential (MEP) maps were generated to visualize the distribution of surface charge.

(1) 𝐸𝑔𝑎𝑝 = (𝐸𝐿𝑈𝑀𝑂 − 𝐸𝐻𝑂𝑀𝑂)
(2) 𝐼 = −𝐸𝐻𝑂𝑀𝑂
(3) 𝐴 = −𝐸𝐿𝑈𝑀𝑂
(4) 𝜒 = (𝐼+𝐴) 2
(5) ω = 𝜇 2 (2𝜂)
(6) 𝜇 = − (𝐼+𝐴) 2
(7) 𝜂 = (𝐼−𝐴) 2
(8) 𝑆 = 1 (2𝜂)

### Evolutionary conservation and structural hotspot mapping of the EGFR kinase domain

To assess the biological relevance of the predicted binding site, a protein-level conservation analysis was performed on the human epidermal growth factor receptor (EGFR). The EGFR kinase-domain structure used in the docking workflow, PDB ID 1M17, was selected as the structural template for residue mapping. Residues implicated in ligand binding were first identified from the docking and molecular dynamics analyses of the present study and then evaluated for evolutionary conservation using the ConSurf framework, which estimates residue-wise conservation from homologous sequence alignments and assigns conservation grades on a scale of 1–9, where higher values indicate stronger conservation [47]. The final conservation scores were mapped onto the EGFR kinase domain and integrated with the previously identified binding-pocket residues. In addition, structurally corresponding hotspot positions relevant to clinically important EGFR mutation sites associated with NSCLC, including L858R, T790M, and C797S, were also examined in the wild-type structural context. Structural visualization and figure preparation were performed in UCSF ChimeraX [48].

### Clinical expression and pharmacogenomic profiling of EGFR in NSCLC

To provide clinical support for the structural findings, EGFR was evaluated in lung adenocarcinoma (LUAD) and lung squamous cell carcinoma (LUSC) using GEPIA3 [49]. Tumor-versus-normal expression and pathological stage analyses were performed to assess the clinical expression profile of EGFR in NSCLC. The Cell Line Screen module of GEPIA3 was additionally used to examine the association between EGFR expression and drug-sensitivity profiles across lung cancer cell lines.

### Mutational landscape analysis of EGFR in lung adenocarcinoma

To further contextualize the structural and pharmacogenomic findings, the mutation distribution of EGFR was examined using cBioPortal, a cancer genomics platform for exploring multidimensional molecular alteration data across large cancer cohorts [50]. A lung adenocarcinoma cohort was queried, and the positional distribution of EGFR alterations across the full-length receptor was visualized using the mutation-domain plot. This analysis was used to summarize the location of recurrent EGFR alterations relative to the receptor architecture, with particular attention to hotspot events in the kinase-domain region.

## Results

### PASS-Based Bioactivity Prediction

The PASS online platform was applied to predict the biological activities of the sixteen selected compounds, generating probability of activity (Pa) and probability of inactivity (Pi) values for five major pharmacological properties, including antineoplastic, apoptosis agonist, antiproliferative, antibacterial, and antifungal activities (Table 2). The PASS prediction spectrum revealed a remarkably high likelihood of anticancer and cytotoxic-related activities among the analyzed compounds, as evidenced by consistently elevated Pa values and minimal corresponding Pi values. In general, predicted activities with Pa > 0.7 were considered high-confidence, Pa values between 0.5 and 0.7 were interpreted as moderate confidence, and Pa < 0.5 indicated low-confidence predictions, consistent with standard PASS interpretation guidelines. However, PASS predictions are based on structure–activity relationships derived from known bioactive compounds and do not account for target-specific binding, dose dependency, or pharmacokinetic behavior; therefore, the results were treated as qualitative indicators and were further validated using molecular docking, ADME profiling, toxicity assessment, and molecular dynamics simulations.

**Table 2:** PASS-based prediction of antineoplastic, apoptosis agonist, antiproliferative, antibacterial, and antifungal activities (Pa/Pi) of selected compounds.

Compound Name	Antineoplastic		Apoptosis agonist		Antiproliferative		Antibacterial		Antifungal	
	Pa	Pi	Pa	Pi	Pa	Pi	Pa	Pi	Pa	Pi
Hongheloside A	0.928	0.005	0.760	0.010	0.952	0.001	0.595	0.009	0.733	0.008
SCHEMBL5933906	0.906	0.005	0.831	0.006	0.938	0.002	0.533	0.014	0.684	0.011
Obebioside A	0.881	0.005	0.763	0.010	0.977	0.001	0.576	0.010	0.743	0.008
Strospeside	0.904	0.005	0.734	0.012	0.951	0.001	0.551	0.012	0.686	0.010
Obeside C	0.904	0.005	0.734	0.012	0.951	0.001	0.551	0.012	0.686	0.010
Obetrioside B	0.908	0.005	0.690	0.016	0.988	0.000	0.669	0.005	0.810	0.005
Obebioside B	0.910	0.005	0.716	0.013	0.983	0.001	0.634	0.007	0.784	0.006
Echujin	0.902	0.005	0.767	0.010	0.983	0.001	0.652	0.006	0.800	0.005
Hongheloside C	0.925	0.005	0.751	0.011	0.982	0.001	0.674	0.005	0.801	0.005
Obebioside C	0.903	0.005	0.726	0.013	0.976	0.001	0.599	0.009	0.743	0.008
Somalin	0905	0.005	0.809	0.008	0.932	0.002	0.680	0.011	0.534	0.013
Obetrioside A	0.881	0.005	0.732	0.012	0.984	0.001	0.613	0.008	0.782	0.006
Neritaloside	0.911	0.005	0.721	0.013	0.966	0.001	0.587	0.009	0.739	0.008
Isokaempferide	0.791	0.013	0.885	0.005	0.606	0.010	0.396	0.031	0.554	0.023
Cardenolide	0.823	0.009	0.783	0.009	0.607	0.010	0.200	0.118	0.277	0.091
Mono-O- acetylacoschimperoside P	0.917	0.005	0.712	0.014	0.954	0.001	0.596	0.009	0.732	0.008

(Pa: Probability of activity; Pi: Probability of inactivity. Compounds with Pa > 0.7 are considered highly active, while Pa values between 0.5 and 0.7 indicate moderate activity.)

For antineoplastic activity, the probability of activity (Pa) values ranged from 0.791 to 0.928, while the probability of inactivity (Pi) remained extremely low (0.005–0.013), indicating strong and highly confident predictions. Among the screened compounds, Hongheloside A demonstrated the highest antineoplastic potential (Pa = 0.928), followed by Hongheloside C (Pa = 0.925), Mono-O-acetylacoschimperoside P (Pa = 0.917), Neritaloside (Pa = 0.911), and Obebioside B (Pa = 0.910). All sixteen compounds displayed Pa values greater than 0.79, satisfying the high-confidence threshold for biological activity prediction. Additionally, the apoptosis against activity revealed Pa values in the range of 0.690 to 0.885, suggesting that the majority of compounds possess a moderate to high capacity to induce programmed cell death mechanisms. Isokaempferide exhibited the highest predicted apoptosis agonist activity (Pa = 0.885), followed by SCHEMBL5933906 (Pa = 0.831) and Somalin (Pa = 0.809). The remaining compounds maintained robust apoptosis-related Pa values, all exceeding 0.690, reinforcing their cytotoxic potential at the molecular level.

Even more pronounced results were observed for antiproliferative activity, which exhibited Pa values ranging from 0.606 to 0.988. Obetrioside B recorded the highest antiproliferative probability (Pa = 0.988; Pi = 0.000), followed by Echujin (Pa = 0.983), Hongheloside C (Pa = 0.982), and Obebioside B (Pa = 0.983). These results point to a strong predicted ability of the screened compounds to inhibit uncontrolled cellular multiplication, a critical feature in anticancer activity screening. In contrast, the antibacterial and antifungal predictions exhibited relatively moderate probabilities. Antibacterial Pa values ranged from 0.200 to 0.680, with the highest activity observed in Somalin (Pa = 0.680), followed by Hongheloside C (Pa = 0.674) and Obetrioside B (Pa = 0.669). Similarly, antifungal Pa values ranged between 0.277 and 0.810, where Obetrioside B showed the maximum predicted antifungal activity (Pa = 0.810), followed by Hongheloside C (Pa = 0.801) and Echujin (Pa = 0.800).

Across all biological activity categories, the probability of inactivity (Pi) remained significantly lower than the corresponding Pa values, reinforcing the reliability of the predicted bioactivities. Notably, antineoplastic and antiproliferative activities consistently demonstrated the highest Pa scores among the screened properties, highlighting these compounds as strong candidates for further cancer-focused computational evaluation. Consequently, based on their superior anticancer-associated predictive scores, these compounds were prioritized for downstream molecular docking, ADMET analysis, and molecular dynamics simulations with selected therapeutic target proteins.

### Molecular docking analysis

Molecular docking was performed to evaluate the predicted binding poses and relative docking scores of *Adenium obesum*-derived phytochemicals within the epidermal growth factor receptor (EGFR) tyrosine kinase domain (PDB ID: 1M17). Docking scores, expressed in kcal/mol, were used as computational ranking metrics within the applied AutoDock Vina protocol, with more negative scores indicating more favorable predicted ligand–receptor interactions according to the docking scoring function. Importantly, these scores represent model-dependent computational estimates and should not be interpreted as experimentally measured binding free energies, equilibrium binding affinities, or inhibitory potencies. The docking results are summarized in Table 3 and demonstrate a heterogeneous distribution of predicted binding scores across the investigated phytochemical library.

**Table 3:** Docking scores (kcal/mol) of Adenium obesum phytochemicals and standard EGFR inhibitors against EGFR (PDB ID: 1M17).

Compound Name	Binding affinity (Kcal/mol)	Number of hydrogen bonds	Number of hydrophobic bonds
Hongheloside A	−8.1	6	1
SCHEMBL5933906	−10	0	2
Obebioside A	−7.8	2	1
Strospeside	−8.2	1	5
Obeside C	−8.3	2	1
Obetrioside B	−8.5	6	1
Obebioside B	−8.6	3	1
Echujin	−9.9	8	1
Hongheloside C	−8.8	5	3
Obebioside C	−7.8	3	1
Somalin	−8.1	1	2
Obetrioside A	−9.4	8	1
Neritaloside	−7.9	3	0
Isokaempferide	−7.9	2	10
Cardenolide	−9.9	1	8
Mono-O-acetylacoschimperoside P	−8.0	1	1
Osimertinib	−7.9	1	6
Afatinib	−8.1	3	8
Erlotinib	−7.0	2	4
Lazertinib	−8.2	3	7

For descriptive purposes, the compounds were grouped according to their relative docking-score ranges to facilitate comparative interpretation within the present dataset. Compounds with the most favorable predicted docking scores (approximately ≤ −9.0 kcal/mol) included SCHEMBL5933906 (−10.0 kcal/mol), Echujin (−9.9 kcal/mol), Cardenolide (−9.9 kcal/mol), and Obetrioside A (−9.4 kcal/mol). Under the applied docking protocol, these compounds received more favorable scores than the reference EGFR-targeting compounds Osimertinib (−7.9 kcal/mol) and Erlotinib (−7.0 kcal/mol). However, these differences should be interpreted strictly as differences in computational docking scores within the same protocol and should not be taken as evidence that these phytochemicals possess greater experimental binding affinity or inhibitory potency than the approved EGFR-targeting drugs. The results instead identify these compounds as candidates for further structural and computational evaluation based on their predicted compatibility with the EGFR binding site.

Compounds with intermediate docking scores, approximately ranging from −8.0 to −9.0 kcal/mol, included Obetrioside B (−8.5 kcal/mol), Obebioside B (−8.6 kcal/mol), and Hongheloside C (−8.8 kcal/mol). Their predicted docking scores were within a similar numerical range to those obtained for the reference compounds Afatinib (−8.1 kcal/mol) and Lazertinib (−8.2 kcal/mol). These numerical similarities indicate comparable ranking under the applied docking protocol; however, they do not establish equivalent binding affinity, inhibitory activity, or pharmacological efficacy. Accordingly, these compounds were considered candidates for further evaluation based on complementary structural, pharmacokinetic, and dynamic analyses rather than on docking scores alone.

Compounds with relatively less favorable docking scores within the investigated set included Obebioside A (−7.8 kcal/mol), Obebioside C (−7.8 kcal/mol), and Neritaloside (−7.9 kcal/mol). Although these compounds received less favorable scores than the highest-ranked phytochemicals, their docking results alone are insufficient to exclude them from further consideration. Their potential relevance should instead be assessed in conjunction with binding-mode plausibility, protein–ligand interaction patterns, physicochemical characteristics, ADMET profiles, and molecular dynamics behavior.

Overall, the docking analysis identified several *A. obesum* phytochemicals with favorable predicted docking scores within the EGFR kinase domain under the applied computational protocol. SCHEMBL5933906, Echujin, Cardenolide, and Obetrioside A were among the top-ranked compounds based on docking score, whereas other phytochemicals exhibited intermediate or relatively less favorable scores. Although several phytochemicals received numerically more favorable docking scores than the reference EGFR-targeting compounds, these findings should not be interpreted as evidence of superior inhibitory potency or therapeutic efficacy. Instead, the docking results served as an initial computational prioritization step for subsequent analyses integrating protein–ligand interaction patterns, drug-likeness, ADMET and toxicity predictions, molecular dynamics simulations, and, ultimately, experimental validation. This integrated approach was used to distinguish compounds with favorable predicted docking characteristics from those with a more balanced overall computational profile for further investigation.

### Validation of molecular docking

The reliability of the molecular docking protocol was validated through re-docking of the co-crystallized native ligand into the ATP-binding cavity of EGFR (PDB ID: 1M17). The predicted docking pose was superimposed onto the experimentally determined crystallographic conformation to evaluate the accuracy and reproducibility of the docking methodology. The detailed validation parameters and docking outcomes are summarized in S3 Table in S1 File. The superimposed structures demonstrated strong spatial agreement between the native and re-docked conformations, particularly within the rigid aromatic scaffold occupying the ATP-binding pocket (S2 Fig in S1 File). The calculated RMSD between the crystallographic and predicted poses was 1.64 Å, indicating excellent reproduction of the experimentally observed binding orientation and confirming the accuracy of the selected docking parameters. These findings validated the reliability and robustness of the docking protocol and supported its suitability for subsequent virtual screening and molecular interaction analyses of the investigated phytochemicals against EGFR.

### Analysis of protein-ligand interactions

Molecular docking of twenty selected compounds against the EGFR kinase domain (PDB ID: 1M17) revealed a broad range of binding affinities, with docking energies spanning from −7.0 to −10.0 kcal·mol^-1^. The highest binding affinity was observed for SCHEMBL5933906 (−10.0 kcal·mol^-1^), followed by Echujin (−9.9 kcal·mol^-1^), Cardenolide (−9.9 kcal·mol^-1^), and Obetrioside A (−9.4 kcal·mol^-1^). Several ligands, including Echujin, Cardenolide, and Obetrioside A, outperformed clinically approved inhibitors such as Osimertinib (−7.9 kcal·mol^-1^), Afatinib (−8.1 kcal·mol^-1^), and Erlotinib (−7.0 kcal·mol^-1^), suggesting their potential as high-affinity EGFR binders.

**Fig 3:**
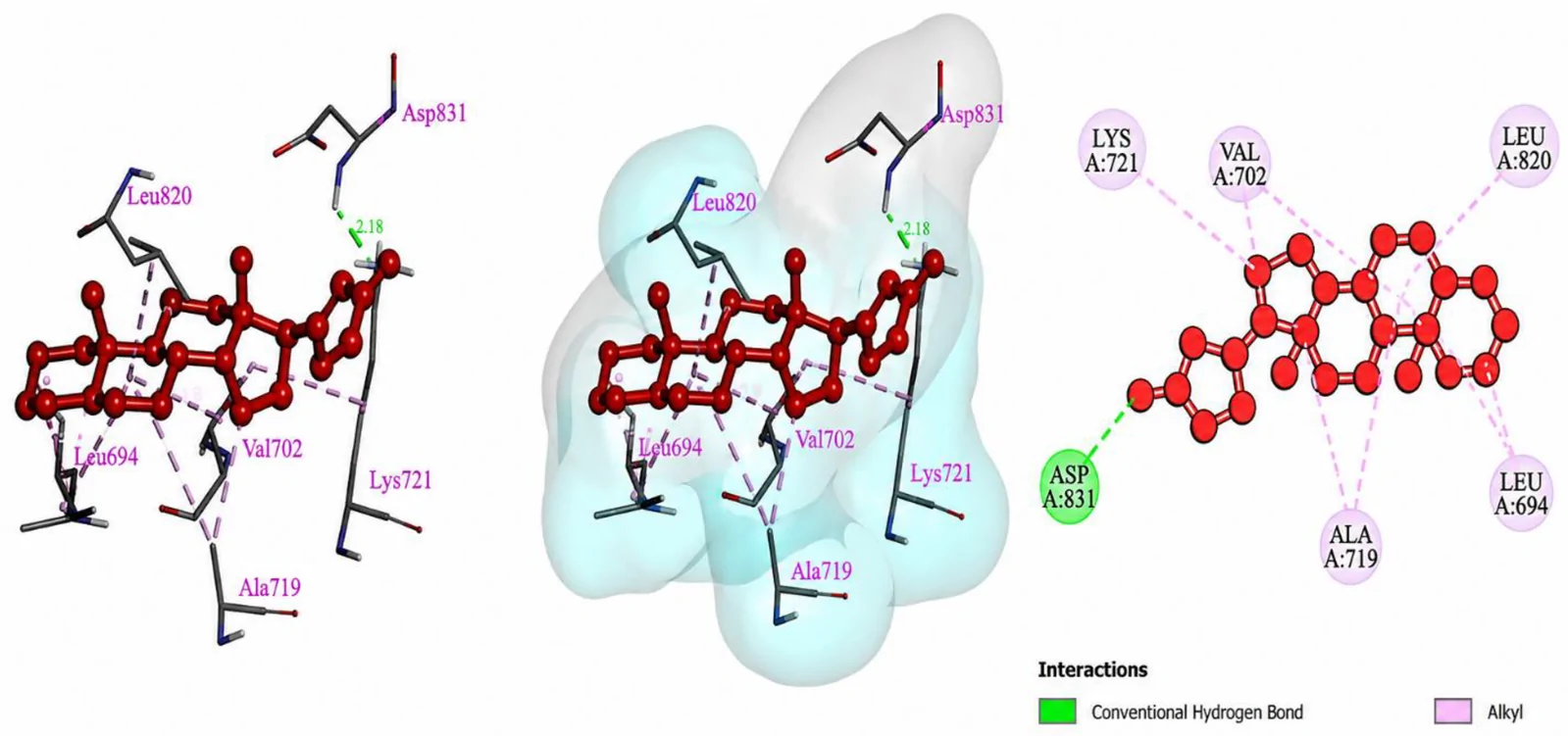
Cardenolide docking poses with EGFR (1M17): 3D ligand orientations in the binding pocket and 2D interaction maps showing H-bonds and hydrophobic.

Detailed inspection of hydrogen bonding patterns highlighted that the most promising compounds formed multiple short, directional hydrogen bonds with key catalytic residues, which are crucial for stabilizing the ligand within the ATP-binding site. The analysis of non-bond interactions is detailed in S4 Table in S1 File, while Fig 3 presents the interaction profile of the selected cardenolide compound; the remaining selected compounds are shown in S3 Fig in S1 File Echujin formed seven hydrogen bonds, including strong conventional interactions with LYS851 (1.89 Å) and MET769 (2.03 Å), as well as moderate bonds with GLU734, GLU738, and additional LYS851 contacts (2.23–2.88 Å). Obetrioside A established eight hydrogen bonds, notably engaging LYS721 (2.04 Å), MET769 (2.07 Å), and PHE699 (2.41–2.78 Å), demonstrating a dense network of short-distance interactions with residues critical for EGFR catalysis. Obetrioside B formed six hydrogen bonds, including a very strong bond with ASP831 (1.97 Å), along with interactions with MET769 and CYS773 (2.27–2.32 Å), highlighting the importance of these conserved residues in stabilizing ligand binding. Conversely, SCHEMBL5933906, despite its highest docking score, lacked strong conventional hydrogen bonds, with reported interactions limited to two hydrophobic contacts at >5.1 Å (LYS721 and MET742). This discrepancy indicates that its predicted binding energy may be primarily driven by non-directional hydrophobic interactions or scoring algorithm bias, underscoring the necessity for pose validation and subsequent MM-GBSA or molecular dynamics evaluation.

Hydrophobic interactions and π-based contacts further contributed to ligand stabilization, with residues LEU694, PHE699, VAL702, ALA719, LEU820, CYS773, and MET742 frequently involved. Cardenolide, for example, combined a strong hydrogen bond with ASP831 (2.16 Å) with multiple alkyl contacts to LEU694, VAL702, and ALA719 (3.98–5.22 Å), reflecting an optimal balance of directional and non-directional stabilization. Other high-ranking compounds, such as Hongheloside A, Obebioside A, and Strospeside, displayed extensive hydrophobic interactions, often complemented by moderate hydrogen bonds (2.7–3.5 Å) with key residues, contributing to their moderate-to-high docking energies (−7.8 to −8.5 kcal·mol^-1^). Analysis of residue-level engagement revealed several conserved “hotspots” critical for ligand recognition: MET769, ASP831, and LYS721 were consistently involved in strong hydrogen bonds, while PHE699, LEU694, VAL702, ALA719, GLU734, GLU738, CYS773, and LYS851 contributed to hydrophobic packing. High-affinity compounds preferentially interacted with multiple hotspots simultaneously, a pattern reminiscent of clinically validated EGFR inhibitors.

Integrating docking energies with detailed interaction patterns, Echujin, Obetrioside A, Obetrioside B, and Cardenolide emerged as the most structurally and energetically plausible ligands. These compounds combined multiple strong hydrogen bonds (<2.2 Å) with key hotspot residues and extensive hydrophobic contacts, indicating favorable enthalpic stabilization and optimal binding geometry. Compounds whose high docking scores relied primarily on long-range hydrophobic interactions, such as SCHEMBL5933906, require additional validation via molecular dynamics simulations to confirm binding stability and reliability. Overall, the protein–ligand interaction analysis demonstrates that several phytochemicals from *Adenium obesum* possess stronger predicted binding affinities than reference EGFR inhibitors. The integration of docking scores, hydrogen bond quality, and hydrophobic contacts provides a robust framework for prioritizing candidates for further molecular dynamics and free energy simulations, supporting their potential as novel EGFR-targeting ligands.

### Molecular docking poses and its interaction analysis

The binding modes of the selected ligands within the EGFR active site were analyzed using BIOVIA Discovery Studio and PyMOL, providing detailed 2D and 3D interaction visualizations of the docked complexes. The analysis revealed a combination of conventional hydrogen bonds, non-conventional hydrogen bonds, and hydrophobic interactions (including π-alkyl, π-sigma, and alkyl interactions) that collectively stabilize the ligand within the ATP-binding pocket. Key hydrogen bond donors and acceptors were identified, highlighting their critical role in orienting the ligands and enhancing binding affinity. The 3D representations illustrated the spatial orientation of each ligand relative to the binding cavity, while the 2D diagrams provided a clear depiction of specific residue interactions, including distances and bond types. As shown in Fig 3 and S3 Fig in S1 File, these visualizations collectively demonstrate the molecular interactions that underpin ligand binding, providing a structural rationale for the observed docking energies and supporting the selection of high-affinity compounds for further molecular dynamics simulations.

### Comparative docking across wild-type and mutant EGFR structures

To further examine the predicted binding behavior of the 16 *Adenium obesum* phytochemicals in clinically relevant EGFR mutation-containing structural backgrounds, all compounds were additionally docked against EGFR structures carrying the L858R/T790M mutation combination (PDB ID: 4I22) and the L858R/T790M/C797S mutation combination (PDB ID: 6LUD). The wild-type EGFR structure (PDB ID: 1M17), used in the initial docking analysis, served as the reference system (Table 4). This comparative analysis was performed to assess whether the predicted docking characteristics of the investigated phytochemicals varied across wild-type and mutation-containing EGFR structural backgrounds.

**Table 4:** Comparative docking scores of 16 *Adenium obesum* phytochemicals against wild-type and clinically relevant mutant EGFR structural backgrounds.

Compound	WT EGFR (1M17)	L858R/T790M (4I22)	L858R/T790M/C797S (6LUD)	Δ vs WT (4I22)	Δ vs WT (6LUD)
Hongheloside A	−8.1	−7.4	−8.4	+0.7	−0.3
SCHEMBL5933906	−10.0	−10.0	−10.8	0.0	−0.8
Obebioside A	−7.8	−8.9	−8.4	−1.1	−0.6
Strospeside	−8.2	−8.2	−9.2	0.0	−1.0
Obeside C	−8.3	−8.9	−9.3	−0.6	−1.0
Obetrioside B	−8.5	−9.2	−8.9	−0.7	−0.4
Obebioside B	−8.6	−9.4	−8.9	−0.8	−0.3
Echujin	−9.9	−9.2	−9.6	+0.7	+0.3
Hongheloside C	−8.8	−9.4	−8.9	−0.6	−0.1
Obebioside C	−7.8	−9.0	−8.8	−1.2	−1.0
Somalin	−8.1	−7.8	−8.9	+0.3	−0.8
Obetrioside A	−9.4	−9.3	−9.9	+0.1	−0.5
Neritaloside	−7.9	−7.5	−9.1	+0.4	−1.2
Isokaempferide	−7.9	−8.0	−7.2	−0.1	+0.7
Cardenolide	−9.9	−8.6	−8.5	+1.3	+1.4
Mono-O-acetylacoschimperoside P	−8.0	−7.1	−8.8	+0.9	−0.8

The docking results demonstrated compound-dependent variation across the three evaluated EGFR structures. SCHEMBL5933906 exhibited consistently favorable predicted docking scores across all three structural backgrounds, with values of −10.0 kcal/mol for wild-type EGFR, −10.0 kcal/mol for the L858R/T790M structure, and −10.8 kcal/mol for the L858R/T790M/C797S structure. Several other phytochemicals also exhibited more negative docking scores in at least one mutation-containing structural background compared with the wild-type structure. For example, Obebioside C showed docking scores of −7.8 kcal/mol against wild-type EGFR, −9.0 kcal/mol against the L858R/T790M structure, and −8.8 kcal/mol against the L858R/T790M/C797S structure. Similarly, Obebioside A, Strospeside, Obeside C, Obetrioside B, Obebioside B, and Hongheloside C exhibited more negative predicted docking scores in at least one of the evaluated mutation-containing structures relative to wild-type EGFR.

Conversely, several compounds exhibited less negative docking scores in one or both mutation-containing structural backgrounds compared with wild-type EGFR. For instance, Cardenolide exhibited docking scores of −9.9 kcal/mol against wild-type EGFR, −8.6 kcal/mol against the L858R/T790M structure, and −8.5 kcal/mol against the L858R/T790M/C797S structure. Despite this relative reduction in docking score compared with the wild-type structure, Cardenolide retained favorable predicted docking characteristics in both mutation-containing structural backgrounds. Echujin similarly showed docking scores of −9.9, −9.2, and −9.6 kcal/mol against wild-type, L858R/T790M, and L858R/T790M/C797S EGFR, respectively. Other compounds, including Hongheloside A, Somalin, Neritaloside, Isokaempferide, and Mono-O-acetylacoschimperoside P, also displayed compound-specific variations in their predicted docking scores across the evaluated structural systems.

Overall, the comparative analysis demonstrated that the predicted docking characteristics of the investigated phytochemicals varied according to the EGFR structural background, with no uniform pattern of either increased or decreased docking scores across all compounds. Some phytochemicals exhibited comparable or more negative predicted docking scores in mutation-containing structures, whereas others showed relative changes in docking scores compared with wild-type EGFR. These observations indicate that mutation-associated structural differences may influence the predicted ligand–EGFR interaction landscape in a compound-dependent manner. Importantly, the docking scores were interpreted only as computational indicators of predicted ligand–protein interaction propensity and were not used as direct measures of experimental binding affinity or inhibitory potency. Furthermore, the present analysis does not establish whether any of the investigated phytochemicals can inhibit mutant EGFR enzymatic activity or overcome clinically established EGFR tyrosine kinase inhibitor resistance. Because the evaluated mutant structures contain combinations of EGFR mutations rather than isolated single mutations, the observed differences cannot be attributed to the independent effects of L858R, T790M, or C797S individually. Therefore, these findings should be considered hypothesis-generating and provide a comparative computational assessment of the predicted interaction behavior of the investigated phytochemicals across wild-type and clinically relevant mutation-containing EGFR structural backgrounds.

### Analysis of drug-like properties

A comprehensive analysis of the physicochemical and pharmacokinetic characteristics of the 16 *Adenium obesum* phytochemicals was performed to evaluate their predicted drug-likeness, compliance with Lipinski’s Rule of Five, and bioavailability-related properties (Table 5). The investigated compounds exhibited substantial physicochemical diversity, with molecular weights ranging from 300.00 to 917.00 g/mol, hydrogen-bond acceptor counts ranging from 2 to 20, hydrogen-bond donor counts ranging from 0 to 9, and topological polar surface areas (TPSA) ranging from 26.30 to 299.00 Å². This broad variation in molecular size, polarity, hydrogen-bonding capacity, and lipophilicity was reflected in differences in predicted Lipinski rule compliance, bioavailability scores, and gastrointestinal (GI) absorption across the investigated compounds.

**Table 5:** Predicted drug-likeness and pharmacokinetic properties of *Adenium obesum*-derived phytochemicals evaluated as computationally prioritized EGFR-binding candidates.

Ligand	Number of rotatable bonds	Hydrogen bond acceptor	Hydrogen bond donor	Topological polar surface area, Å2	Consensus Log Po/w	Lipinski rule		Molecular weight (g/mol)	Bioavailability Score	Gastrointestinal absorption
						Result	violation			
Hongheloside A	6	9	2	120.75	2.93	Yes	1	576.72	0.55	High
SCHEMBL5933906	7	13	5	182.83	2.52	No	2	764.94	0.17	Low
Obebioside A	7	13	6	193.83	1.27	No	3	696.82	0.17	Low
Strospeside	4	9	4	134.91	1.54	Yes	1	550.68	0.55	High
Obeside C	4	6	4	134.91	1.69	Yes	1	550.68	0.55	High
Obetrioside B	12	20	9	299.00	1.70	No	3	917.00	0.17	Low
Obebioside B	9	15	6	183.99	0.84	No	3	754.86	0.17	Low
Echujin	10	17	8	252.75	0.21	No	3	843.00	0.17	Low
Hongheloside C	9	14	5	199.90	1.59	No	2	738.86	0.17	Low
Obebioside C	7	14	7	214.06	0.33	No	3	712.82	0.17	Low
Somalin	4	7	2	94.45	3.52	Yes	1	518.68	0.55	High
Obetrioside A	10	18	9	273.00	1.24	No	3	859.00	0.17	Low
Neritaloside	6	10	3	140.98	2.41	Yes	1	592.72	0.55	Low
Isokaempferide	2	6	3	100.13	1.94	Yes	0	300.00	0.55	High
Cardenolide	1	2	0	26.3	5.22	Yes	1	342.00	0.55	High
Mono-O-acetylacoschimperoside P	8	11	2	147.05	2.83	No	2	634.75	0.17	Low

Six compounds, Hongheloside A, Strospeside, Obeside C, Somalin, Isokaempferide, and Cardenolide showed comparatively favorable drug-likeness profiles, with no more than one predicted Lipinski rule violation. Among these compounds, molecular weights ranged from 300.00 g/mol for Isokaempferide to 592.72 g/mol for Neritaloside, while TPSA values ranged from 26.30 to 140.98 Å². Isokaempferide and Cardenolide exhibited the lowest molecular weights within this group, at 300.00 and 342.00 g/mol, respectively, and both contained a limited number of rotatable bonds. These compounds, together with Hongheloside A, Strospeside, Obeside C, and Somalin, showed a predicted bioavailability score of 0.55. High predicted GI absorption was observed for Hongheloside A, Strospeside, Obeside C, Somalin, Isokaempferide, and Cardenolide, whereas Neritaloside, despite exhibiting only one predicted Lipinski violation, showed low predicted GI absorption.

In contrast, several phytochemicals exhibited multiple predicted Lipinski rule violations. SCHEMBL5933906, Obebioside A, Obetrioside B, Obebioside B, Echujin, Hongheloside C, Obebioside C, Obetrioside A, and Mono-O-acetylacoschimperoside P showed two or three predicted violations. These compounds generally possessed higher molecular weights (634.75–917.00 g/mol), greater hydrogen-bonding capacities, and elevated TPSA values. In particular, Obetrioside B exhibited the highest molecular weight (917.00 g/mol) and TPSA (299.00 Å²) among the investigated compounds. Echujin also showed a relatively high molecular weight (843.00 g/mol), nine hydrogen-bond donors, 17 hydrogen-bond acceptors, and a TPSA of 252.75 Å². These physicochemical characteristics may present challenges for passive membrane permeability and oral absorption.

The predicted bioavailability scores and GI absorption profiles were broadly consistent with the observed physicochemical characteristics. Most compounds with no more than one predicted Lipinski violation showed a bioavailability score of 0.55, whereas compounds with two or three predicted violations generally exhibited a lower predicted bioavailability score of 0.17. Similarly, the majority of compounds with multiple Lipinski violations showed low predicted GI absorption. However, Lipinski rule compliance did not uniformly correspond to high predicted absorption, as Neritaloside demonstrated one predicted violation but low GI absorption. These findings indicate that drug-likeness and absorption are influenced by multiple interacting physicochemical properties rather than by Lipinski rule compliance alone. The predicted consensus Log Po/w values ranged from 0.21 to 5.22, indicating considerable variation in lipophilicity among the investigated phytochemicals. Several compounds showed moderate predicted lipophilicity, whereas Cardenolide exhibited the highest predicted consensus Log Po/w value (5.22). This relatively high predicted lipophilicity may represent a potential developability consideration and should be evaluated alongside its other physicochemical and pharmacokinetic characteristics. Similarly, the predicted water solubility values varied considerably among the compounds, further demonstrating that favorable drug-likeness parameters in one dimension do not necessarily indicate an optimal overall pharmacokinetic profile.

Overall, the drug-likeness assessment highlights a clear distinction between the smaller, less polar compounds and the larger, highly polar glycosylated phytochemicals. Isokaempferide, Cardenolide, Somalin, and Hongheloside A exhibited comparatively favorable predicted drug-likeness and absorption-related profiles, with physicochemical characteristics that may be more compatible with further oral drug-development investigation. However, these findings should not be interpreted as evidence of confirmed oral bioavailability or therapeutic suitability. Compounds with multiple predicted Lipinski rule violations and low gastrointestinal absorption may still possess pharmacological potential, but their predicted physicochemical and pharmacokinetic characteristics could present additional challenges that may require structural optimization, formulation strategies, alternative delivery approaches, or experimental pharmacokinetic evaluation. Collectively, these findings emphasize the importance of considering drug-likeness and predicted pharmacokinetic characteristics alongside target-binding potential when prioritizing natural-product candidates for further investigation.

### ADME analysis

The pharmacokinetic properties of the selected *Adenium obesum*–derived phytochemicals were evaluated (Table 6) using in silico absorption, distribution, metabolism, and excretion (ADME) profiling in order to assess their suitability as potential therapeutic candidates against non-small-cell lung cancer (NSCLC). Key parameters including water solubility (Log S), Caco-2 cell permeability, human intestinal absorption, volume of distribution at steady state (VDss), blood–brain barrier (BBB) permeability, cytochrome P450 (CYP) inhibition potential, total clearance, and renal OCT2 substrate affinity were analyzed and compared.

**Table 6:** Predicted ADME (Absorption, Distribution, Metabolism, and Excretion) properties of selected Adenium obesum phytochemicals.

Ligand	Absorption			Distribution		Metabolism		Excretion	
	Water Solubility Log S	Caco-2 Permeability (10-6 cm/s)	Intestinal absorption (human)%	VDss (human) (log L/kg)	B.B.B Permeability	CYP450 1A2 Inhibitor	CYP450 2C9 Inhibitor	Total clearance (ml/min/kg)	Renal OCT2 substrate
Hongheloside A	-5.577	0.726	95.618	-0.178	-1.011	No	No	0.464	No
SCHEMBL5933906	-4.410	0.601	74.287	0.259	-1.364	No	No	0.445	No
Obebioside A	-4.308	0.467	38.142	-0.376	-1.574	No	No	0.617	No
Strospeside	-5.163	0.711	72.262	-0.142	-0.952	No	No	0.585	No
Obeside C	-5.163	0.711	72.262	-0.142	-0.952	No	No	0.585	No
Obetrioside B	-2.921	-0.924	19.174	-0.209	-2.336	No	No	0.553	No
Obebioside B	-4.111	0.359	39.650	-0.410	-1.921	No	No	0.538	No
Echujin	-3.080	-0.699	33.729	-0.246	-1.941	No	No	0.605	No
Hongheloside C	-4.105	-0.350	65.178	-0.617	-2.061	No	No	0.42	No
Obebioside C	-3.846	-0.360	51.295	-0.647	-1.898	No	No	0.543	No
Somalin	-5.131	1.457	96.750	0.332	-0.616	No	No	0.484	No
Obetrioside A	-3.382	-0.560	34.518	-0.867	-2.474	No	No	0.527	No
Neritaloside	-4.748	-0.150	81.954	-0.208	-1.485	No	No	0.433	No
Isokaempferide	-3.577	0.374	84.046	-0.954	-0.907	Yes	No	0.579	No
Cardenolide	-5.166	1.370	98.343	0.765	0.239	No	No	0.478	No
Mono-O-acetylacoschimperoside P	-5.555	0.428	90.622	-0.233	-1.410	No	No	0.385	No

Most of the investigated phytochemicals exhibited moderate to high predicted intestinal absorption, indicating favorable oral bioavailability potential. Compounds such as Cardenolide (98.34%), Somalin (96.75%), Hongheloside A (95.62%), and Mono-O-acetylacoschimperoside P (90.62%) demonstrated particularly strong absorption profiles. Moderate absorption was also observed for Neritaloside (81.95%), Isokaempferide (84.05%), and Strospeside (72.26%), suggesting these compounds may be efficiently absorbed through the human gastrointestinal tract. In contrast, a few compounds such as Obetrioside B (19.17%) and Echujin (33.73%) displayed comparatively lower absorption rates, indicating relatively limited bioavailability. Additionally, this absorption trend was further supported by Caco-2 permeability predictions, where Somalin (1.457 × 10 − ⁶ cm/s) and Cardenolide (1.370 × 10 − ⁶ cm/s) showed the highest permeability, followed by Hongheloside A (0.726 × 10 − ⁶ cm/s) and Strospeside (0.711 × 10 − ⁶ cm/s). These findings indicate that the leading compounds have a strong capacity to cross intestinal epithelial barriers via passive diffusion. However, several compounds such as Echujin, Obetrioside B, and Hongheloside C showed lower permeability values, which may limit their absorption efficiency. All compounds exhibited low aqueous solubility (log S values between −2.92 and −5.58), which is characteristic of many plant-derived secondary metabolites. Although low solubility may present formulation challenges, it is often associated with improved membrane permeability and enhanced interaction with hydrophobic protein targets such as EGFR.

In terms of distribution, most phytochemicals displayed moderate predicted volumes of distribution (VDss), ranging from −0.867 to 0.765 log L/kg. Cardenolide (0.765) and Somalin (0.332) indicated broader systemic distribution, which may enhance access to tumor tissues. Meanwhile, compounds such as Isokaempferide (−0.954) and Obebioside C (−0.647) showed more restricted tissue distribution, suggesting confinement primarily within the plasma compartment. Moreover, Blood–brain barrier (BBB) permeability prediction revealed that nearly all investigated phytochemicals demonstrated negative BBB scores, indicating limited penetration into the central nervous system. Notably, Cardenolide exhibited a marginally positive BBB value (0.239), suggesting only a weak potential for CNS permeation. Although slight BBB permeability is generally undesirable for peripheral cancer targets, this low positive value remains within an acceptable range and is unlikely to result in significant neurological exposure. Moreover, such limited permeability could be contextually beneficial in cases where metastasis to the brain occurs in advanced NSCLC.

Furthermore, the metabolic profiles of the selected *Adenium obesum* phytochemicals were largely favorable. The majority of compounds were predicted not to inhibit key cytochrome P450 (CYP) isoforms, indicating a low likelihood of adverse drug–drug interactions. Among the tested phytochemicals, Isokaempferide was predicted to inhibit CYP1A2, whereas all other compounds showed no inhibitory activity toward CYP1A2 or CYP2C9. These findings suggest that, with the exception of Isokaempferide, the phytochemicals are unlikely to interfere significantly with hepatic metabolism, supporting their favorable pharmacokinetic profiles for potential use in combination therapies targeting non-small-cell lung cancer.

The predicted excretion profiles of the selected *Adenium obesum* phytochemicals indicated a range of clearance rates, reflecting balanced systemic retention across the panel. Total clearance values ranged from 0.385 to 0.617 ml/min/kg, representing slow, moderate, and relatively faster elimination. Compounds such as Mono-O-acetylacoschimperoside P (0.385 ml/min/kg) and Hongheloside C (0.420 ml/min/kg) exhibited slower clearance, suggesting prolonged systemic exposure, whereas Obebioside A (0.617 ml/min/kg) and Echujin (0.605 ml/min/kg) showed relatively higher clearance, indicative of faster elimination. Several compounds, including Cardenolide (0.478 ml/min/kg), SCHEMBL5933906 (0.445 ml/min/kg), and Neritaloside (0.433 ml/min/kg), display moderate clearance, providing an optimal balance between sufficient plasma retention and effective elimination. Importantly, none of the phytochemicals were predicted to be substrates of renal OCT2 transporters, reducing the risk of transporter-mediated renal accumulation or nephrotoxicity. Overall, the excretion profiles suggest that most compounds possess excretion profiles compatible with safe and potential therapeutic use.

Overall, the integrated ADME profiling highlights Cardenolide, Somalin, Hongheloside A, Neritaloside, and Mono-O-acetylacoschimperoside P as the most pharmacokinetically promising candidates, displaying a favorable balance of high intestinal absorption, adequate permeability, moderate distribution, CYP inhibition, and safe excretion potential. These pharmacokinetic advantages, combined with their strong molecular docking affinities toward EGFR, support their selection for further molecular dynamics simulations and advanced pharmacological investigation in the context of NSCLC targeted therapy.

### Toxicity analysis

The in-silico toxicity profiling of the selected *Adenium obesum* phytochemicals revealed a broadly favorable safety landscape, strengthening their suitability as potential EGFR-targeted therapeutic candidates for non-small-cell lung cancer (NSCLC), as summarized in Table 7. Notably, none of the screened compounds exhibited predicted hERG channel inhibition, indicating a minimal risk of cardiotoxicity, a critical parameter in anticancer drug development. The acute oral toxicity predictions, expressed as LD₅₀ values in rats, ranged from 2.387 to 3.706 mol/kg, suggesting an overall low to moderate toxicity profile across the compound library. Compounds such as SCHEMBL5933906 (3.706 mol/kg), Hongheloside C (3.156 mol/kg), Obebioside C (3.159 mol/kg), and Obebioside B (3.232 mol/kg) demonstrated comparatively higher LD₅₀ values, indicating lower acute toxicity and improved safety margins. Conversely, Somalin (2.387 mol/kg) and Isokaempferide (2.395 mol/kg) presented relatively lower LD₅₀ values, suggesting a moderately increased acute toxicity, albeit still within a tolerable range for further investigation.

**Table 7:** In silico toxicity profiling of selected Adenium obesum phytochemicals based on key safety parameters.

Compound name	hERG inhibitor	LD50 of rat (Mol/kg)	Oral Rat Chronic Toxicity	AMES test	Skin sensitization	Hepatoxicity	Max. tolerated dose (human)
Hongheloside A	No	2.589	1.266	No	No	No	−1.282
SCHEMBL5933906	No	3.706	2.832	No	No	No	−2.078
Obebioside A	No	3.141	**2.75**	No	No	No	−1.987
Strospeside	No	2.609	1.771	Yes	No	No	−1.110
Obeside C	No	2.609	1.771	Yes	No	No	−1.110
Obetrioside B	No	2.993	3.092	No	No	No	−1.771
Obebioside B	No	3.232	2.897	No	No	No	−1.949
Echujin	No	3.112	3.051	No	No	No	−2.135
Hongheloside C	No	3.156	2.965	No	No	No	−2.046
Obebioside C	No	3.159	2.879	No	No	No	−1.955
Somalin	No	2.387	1.207	No	No	No	−1.036
Obetrioside A	No	3.028	2.945	No	No	No	−1.976
Neritaloside	No	2.676	1.822	No	No	No	−1.237
Isokaempferide	No	2.395	1.704	No	No	No	0.340
Cardenolide	No	2.489	2.145	No	No	No	−0.376
Mono-O-acetylacoschimperoside P	No	2.623	1.296	No	No	No	−1.306

Oral rat chronic toxicity values largely remained within a moderate window (1.207–3.092), with Obetrioside B (3.092), Echujin (3.051), and Hongheloside C (2.965) exhibiting higher thresholds for chronic exposure tolerability. Importantly, the vast majority of the phytocompounds tested negative in the AMES mutagenicity assay, reflecting a reduced likelihood of genotoxic or carcinogenic effects. Only Strospeside and Obeside C displayed positive AMES predictions, warranting cautious interpretation and prioritizing them lower in the lead selection process. Furthermore, all compounds were predicted to be non-sensitizing to the skin and non-hepatotoxic, underscoring a favorable systemic safety profile with minimal risk of liver-associated adverse effects, a key concern in chemotherapeutic regimens.

The predicted maximum tolerated dose (MTD) in humans demonstrated compound-specific variation, with most values remaining within a negative log-scale range, suggestive of acceptable dose tolerability for therapeutic application. Interestingly, Isokaempferide exhibited a slightly positive MTD value (0.340), implying relatively higher dose tolerance in comparison to other screened constituents. In contrast, SCHEMBL5933906 (−2.078), Echujin (−2.135), and Hongheloside C (−2.046) indicated comparatively lower human dose thresholds, which may necessitate careful dose optimization. Among the evaluated candidates, Cardenolide emerged as a particularly balanced compound, displaying no cardiotoxic, hepatotoxic, mutagenic, or sensitizing effects, along with moderate acute and chronic toxicity values (LD₅₀ = 2.489 mol/kg; chronic toxicity = 2.145) and a reasonable predicted human tolerability (MTD = −0.376). This overall toxicity signature, combined with its favorable performance in the preceding in silico analyses, positions Cardenolide as one of the most promising and pharmacologically safe lead candidates from *Adenium obesum* for further experimental validation in NSCLC therapy. Collectively, the toxicity evaluation highlights that the majority of the investigated phytochemicals possess an acceptable safety and tolerability profile, thereby supporting their progression into advanced preclinical and experimental validation phases as potential EGFR target candidate for lung cancer treatment.

### Molecular dynamics simulation

To validate the structural stability and dynamic behavior of the top-ranked *Adenium obesum* phytochemical Cardenolide –EGFR complex obtained from molecular docking, a 100 ns all-atom molecular dynamics (MD) simulation was performed under physiological conditions using the Desmond simulation package. MD simulations were employed to move beyond static docking poses and evaluate whether the ligand–protein complex remains dynamically stable, structurally coherent, and energetically favorable under explicit solvent conditions.

### Protein–ligand RMSD analysis

**Fig 4:**
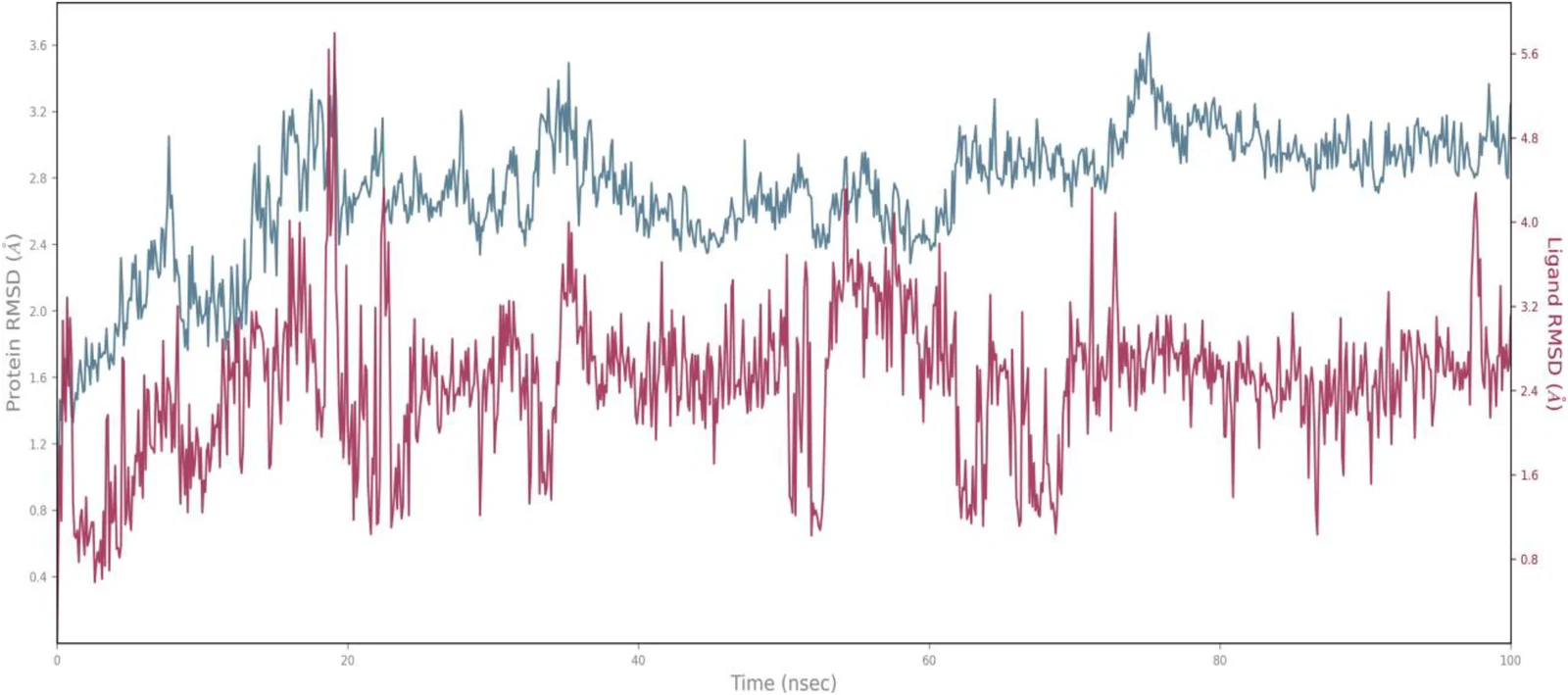
Protein–ligand RMSD analysis of the EGFR in complex with the *Adenium obesum* phytochemical Cardenolide during molecular dynamics simulation.

Protein–ligand RMSD analysis (Fig 4) was performed to assess the structural stability and equilibration behavior of the EGFR–Cardenolide phytochemical complex during the MD simulation. Following backbone alignment to the reference structure, the EGFR kinase domain rapidly equilibrated and maintained RMSD fluctuations within ~1–3 Å throughout the production phase, consistent with a stable, globular kinase fold. The absence of progressive drift or large-amplitude deviations indicates that the system reached thermodynamic equilibrium and that ligand binding does not induce destabilizing global conformational rearrangements.

Ligand stability, evaluated as RMSD relative to the protein backbone, closely mirrored the protein RMSD profile across the trajectory. The lack of abrupt RMSD excursions or sustained increases confirms persistent ligand retention within the ATP-binding cleft, with no evidence of dissociation or pocket migration. Such coherence between protein and ligand RMSD is indicative of a robust and dynamically stable binding mode. Together, these results demonstrate that the EGFR–Cardenolide complex remains structurally stable under explicit-solvent conditions, validating that the predicted binding pose is maintained beyond static docking and persists throughout long-timescale molecular dynamics simulation.

### Residue-Level Flexibility and Binding Pocket Dynamics (RMSF Analysis)

**Fig 5:**
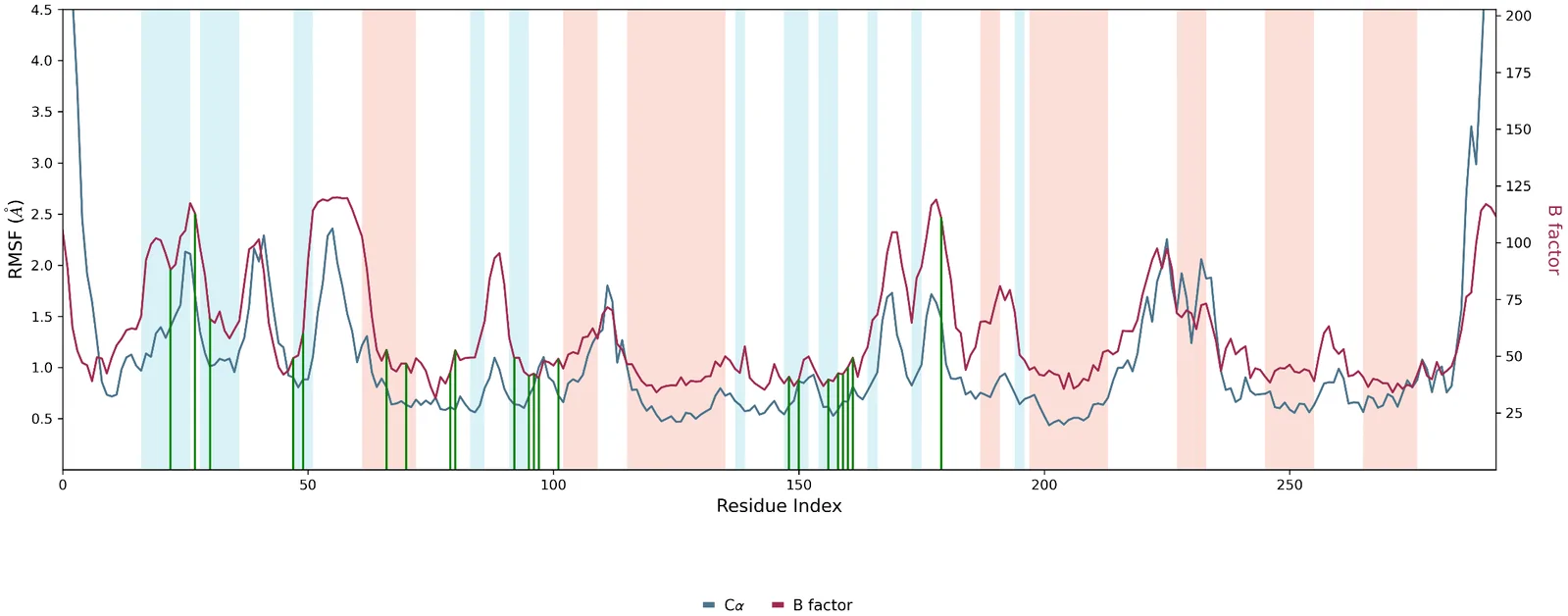
Residue-level flexibility of EGFR in complex with the *Adenium obesum* phytochemical Cardenolide.

Residue-wise RMSF analysis (Fig 5) reveals that EGFR maintains a dynamically stable conformation upon binding the *Adenium obesum* phytochemical Cardenolide, with the majority of residues fluctuating below ~1.5 Å throughout the 100 ns simulation. Elevated flexibility is primarily confined to the N- and C-terminal regions, consistent with their solvent-exposed and functional peripheral nature. In contrast, residues directly involved in ligand binding exhibit markedly suppressed fluctuations, indicating ligand-induced stabilization of the kinase active site. This localized rigidity suggests effective conformational restraint of the binding pocket, a hallmark of productive EGFR inhibition. Moderate flexibility observed in adjacent loop regions likely reflects adaptive motion rather than destabilization, enabling conformational accommodation without compromising binding integrity. Notably, the RMSF profile qualitatively aligns with experimental B-factor trends, supporting the physiological relevance of the simulated dynamics. Collectively, these results demonstrate that the Cardenolide stabilizes EGFR through selective suppression of residue-level mobility at functionally critical regions, reinforcing its potential as a computationally prioritized candidate for targeted therapy in non-small-cell lung cancer.

### Protein–ligand contact analysis

**Fig 6:**
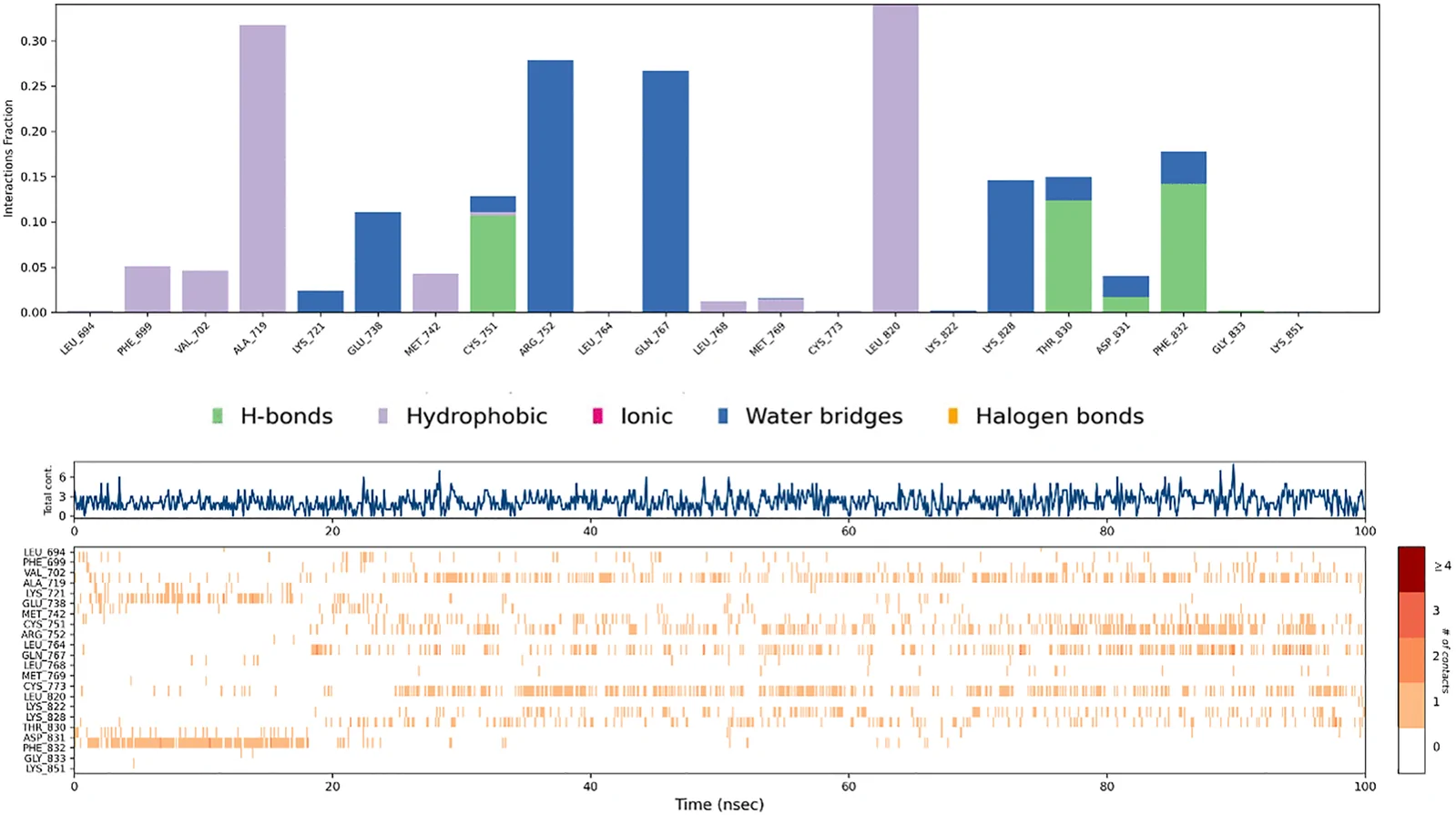
Protein–Ligand Contact Landscape During 100-ns MD Simulation. (A) Fractional interaction plot showing hydrogen bonds, hydrophobic contacts, ionic interactions, water bridges, and halogen bonds formed between the ligand and EGFR residues across the trajectory. (B) Contact timeline map illustrating the temporal persistence of interactions across the simulation.

Protein–ligand contact profiling across the 100-ns MD simulation revealed a highly stable and cooperative interaction network between the *Adenium obesum* phytochemical Cardenolide and the EGFR kinase domain, indicating a well-defined and persistent binding mode essential for high-affinity inhibition. Throughout the trajectory, the ligand consistently engaged multiple residues spanning the hinge region, catalytic loop, and activation loop, forming a dense interaction architecture that remained preserved over time. The fractional contact histogram (Fig 6A) demonstrated that hydrophobic residues such as ALA719, PHE699, LEU820, VAL702, and MET742 made the largest contributions to ligand stabilization, with ALA719 and LEU820 displaying the highest fractional occupancies (>0.30). These residues constitute the EGFR hydrophobic core, and their extensive involvement suggests that the ligand embeds deeply within the ATP-binding cleft, exploiting van der Waals complementarity to restrict pocket fluctuations. Such persistent hydrophobic anchoring is a hallmark of clinically validated EGFR inhibitors and is typically associated with reduced dissociation frequency and strong binding enthalpy. In addition to hydrophobic interactions, the ligand formed highly stable hydrogen bonds with CYS751, THR830, ASP831 and PHE832, all of which showed notable fractional contact contributions (0.10–0.15). The presence of CYS751, a key catalytic-loop residue within the hydrogen-bonding network, is particularly significant as this interaction often governs the inhibitory mechanism in ATP-competitive EGFR inhibitors. The combined contribution of THR830 and ASP831 further strengthens the polar anchoring effect, ensuring that the ligand maintains its orientation even during subtle conformational breathing of the active site. The contact landscape also revealed frequent water-mediated interactions, especially with GLU738, ARG752, GLN767, PHE832, and THR830. Water bridges in EGFR are functionally important stabilizers, and their consistent formation suggests that the ligand efficiently integrates into the solvent shell of the pocket, allowing it to remain stably coordinated as local hydration layers reorganize during the simulation.

Temporal interaction mapping across the entire 100-ns trajectory (Fig 6B) confirmed that the ligand maintained simultaneous contacts with 6–8 residues at nearly all timepoints, indicating a low probability of detachment and strong pocket residency. Several residues including PHE699, ALA719, CYS751, ARG752, THR830, and ASP831 showed dense, continuous contact patterns, reaffirming their roles as core stabilizing anchors. While hydrophobic residues displayed short intermittent bursts of contact, characteristic of flexible van der Waals interactions, polar residues preserved more continuous interactions, consistent with a rigid hydrogen-bond–based stabilization network. This mixed dynamic behavior rigid polar anchoring complemented by adaptive hydrophobic contacts is highly desirable for kinase inhibitor design, as it often correlates with high specificity and effective binding free energy. Mechanistically, the observed interaction patterns indicate that the phytochemical engages the EGFR ATP pocket in a manner analogous to known therapeutic EGFR inhibitors such as erlotinib and gefitinib. The strong hinge-region interactions with ALA719 and MET742, together with catalytic loop engagement at CYS751 and activation-loop involvement at THR830 and PHE832, highlight a binding mode that restricts ATP access and potentially disrupts kinase activation. The persistence and diversity of contacts, including the contribution of long-lived water bridges, further suggest that the ligand remains both geometrically and energetically favored within the active site. The combination of stable hydrophobic packing, well-positioned hydrogen bonding, and adaptive solvent-mediated interactions supports a robust inhibitory mechanism and aligns strongly with the low ligand RMSD, and favorable binding energy values associated with potent inhibitors.

Overall, the protein–ligand contact analysis provides compelling evidence that the Adenium obesum phytochemical Cardenolide is not only capable of stably occupying the EGFR active site, but also of establishing a sophisticated and persistent interaction network typically associated with highly potential kinase inhibitors. The long-lived contacts distributed across structural hot-spot residues, the continuous contact multiplicity throughout the trajectory, and the coordinated behavior of hydrophobic, polar, and water-bridged interactions collectively suggest that the Cardenolide possesses a strong inhibitory potential suitable for further optimization as a targeted therapeutic candidate for NSCLC.

Additionally, secondary structure element (SSE) analysis (S4 Fig in S1 File) demonstrated that approximately 46% of the protein structure was maintained as stable helices and β-strands throughout the simulation, with ~30% α-helical and ~16% β-sheet content. The temporal persistence of these elements confirms that ligand binding does not induce secondary structure destabilization or unfolding. From a mechanistic perspective, preservation of the kinase domain’s secondary structure is critical, as EGFR inhibitors must selectively restrain enzymatic activity without triggering non-physiological conformational collapse. The observed SSE stability therefore reinforces the structural compatibility of the Cardenolide with the EGFR binding landscape.

Moreover, ligand RMSF (S5 Fig in S1 File and torsional analyses (S6 Fig in S1 File) revealed limited internal flexibility, with only a small number of rotatable bonds exhibiting controlled conformational transitions. This restrained flexibility is advantageous from a drug design perspective, as excessive torsional freedom often incurs entropic penalties upon binding. The torsion profile demonstrated that the ligand predominantly occupied low-energy conformational states while bound to EGFR, indicating minimal conformational strain. Such behavior suggests that the ligand’s bioactive conformation is energetically favorable and does not require significant distortion to maintain binding a hallmark of efficient and selective inhibitors. Furthermore, stable radius of gyration (rGyr) and solvent-accessible surface area (SASA) values indicate that the ligand maintained a compact, well-buried conformation within the binding pocket, reducing solvent exposure and enhancing hydrophobic complementarity. These physicochemical trends collectively point toward favorable enthalpy–entropy compensation during binding. Also, Time-dependent analysis of ligand physicochemical Cardenolide properties (S7 Fig in S1 File) further corroborated binding stability. The radius of gyration remained essentially constant, indicating that the ligand neither collapsed nor excessively extended during the simulation. Solvent-accessible surface area (SASA) and polar surface area (PSA) exhibited only minor fluctuations, reflecting consistent burial of hydrophobic moieties and stable exposure of polar functional groups. Moreover, the limited presence of intramolecular hydrogen bonds suggests that ligand–protein interactions are favored over internal ligand stabilization, reinforcing the notion that binding is driven by productive intermolecular contacts rather than intramolecular constraints.

Taking together, the molecular dynamics simulation results provide compelling dynamic evidence that the selected *Adenium obesum* phytochemical Cardenolide forms a structurally stable, energetically favorable, and dynamically resilient complex with EGFR. The combined analyses of RMSD, RMSF, secondary structure preservation, interaction persistence, and ligand conformational behavior collectively validate the compound’s inhibitory potential at an atomistic level. These findings strongly justify further free energy calculations and experimental validation, positioning the identified phytochemical Cardenolide as a lead potential candidate for EGFR-targeted therapy against non-small-cell lung cancer.

### Principal Component Analysis (PCA)

Principal component analysis (PCA) was performed to investigate the dominant collective motions and conformational dynamics of the protein–ligand complex throughout the 100 ns molecular dynamics simulation. The projection of the trajectory onto the first two principal components (PC1 and PC2) revealed that PC1 accounted for 33.1% of the total variance, whereas PC2 contributed 19.2%, together explaining 52.3% of the overall conformational motion observed during the simulation.

**Fig 7:**
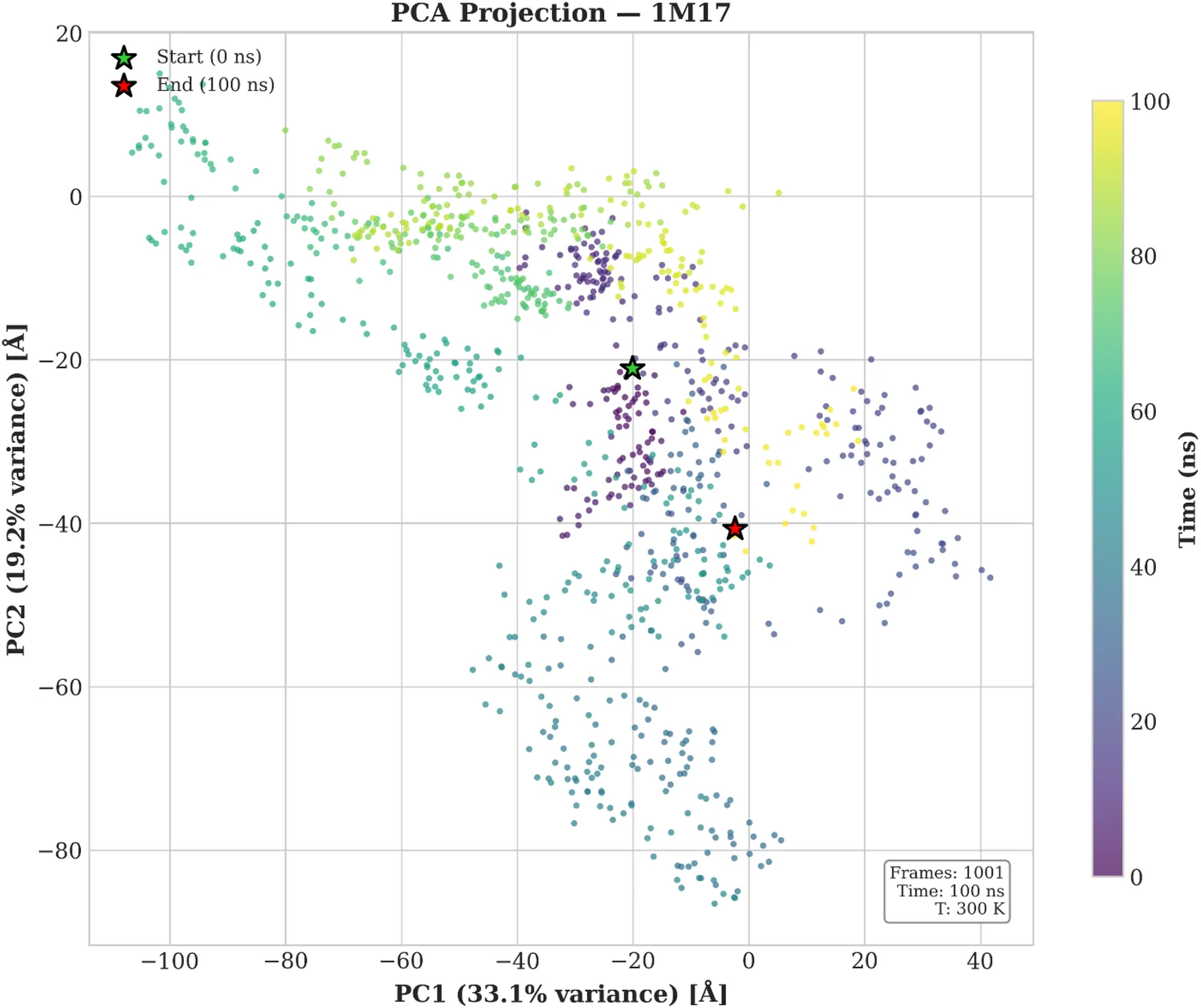
Principal Component Analysis (PCA).

The PCA projection demonstrated (Fig 7) broad conformational sampling across the simulation trajectory, indicating that the complex explored multiple structural states during the simulation period. The trajectory initially occupied a localized region in conformational space and gradually expanded toward distinct regions along both PC1 and PC2 axes, suggesting progressive structural adaptation of the protein–ligand complex over time. The distribution of frames showed a continuous transition pattern rather than abrupt or highly scattered movements, indicating stable conformational evolution during the simulation. Several populated clusters were observed within the PCA landscape, reflecting the presence of metastable conformational states. These clustered regions imply that the complex adopted multiple energetically favorable conformations while maintaining structural integrity throughout the simulation. The separation between the starting and ending frames further suggests a conformational shift from the initial structure toward an adapted equilibrium state, likely associated with ligand accommodation within the binding pocket. Overall, the PCA results indicate that the complex underwent controlled conformational fluctuations while preserving dynamic stability across the 100 ns simulation. The absence of extreme dispersion or isolated outliers suggests that the system remained structurally coherent, supporting the stability of the ligand-bound complex during molecular dynamics simulation.

### Electronic Reactivity and DFT-based descriptors

**Fig 8:**
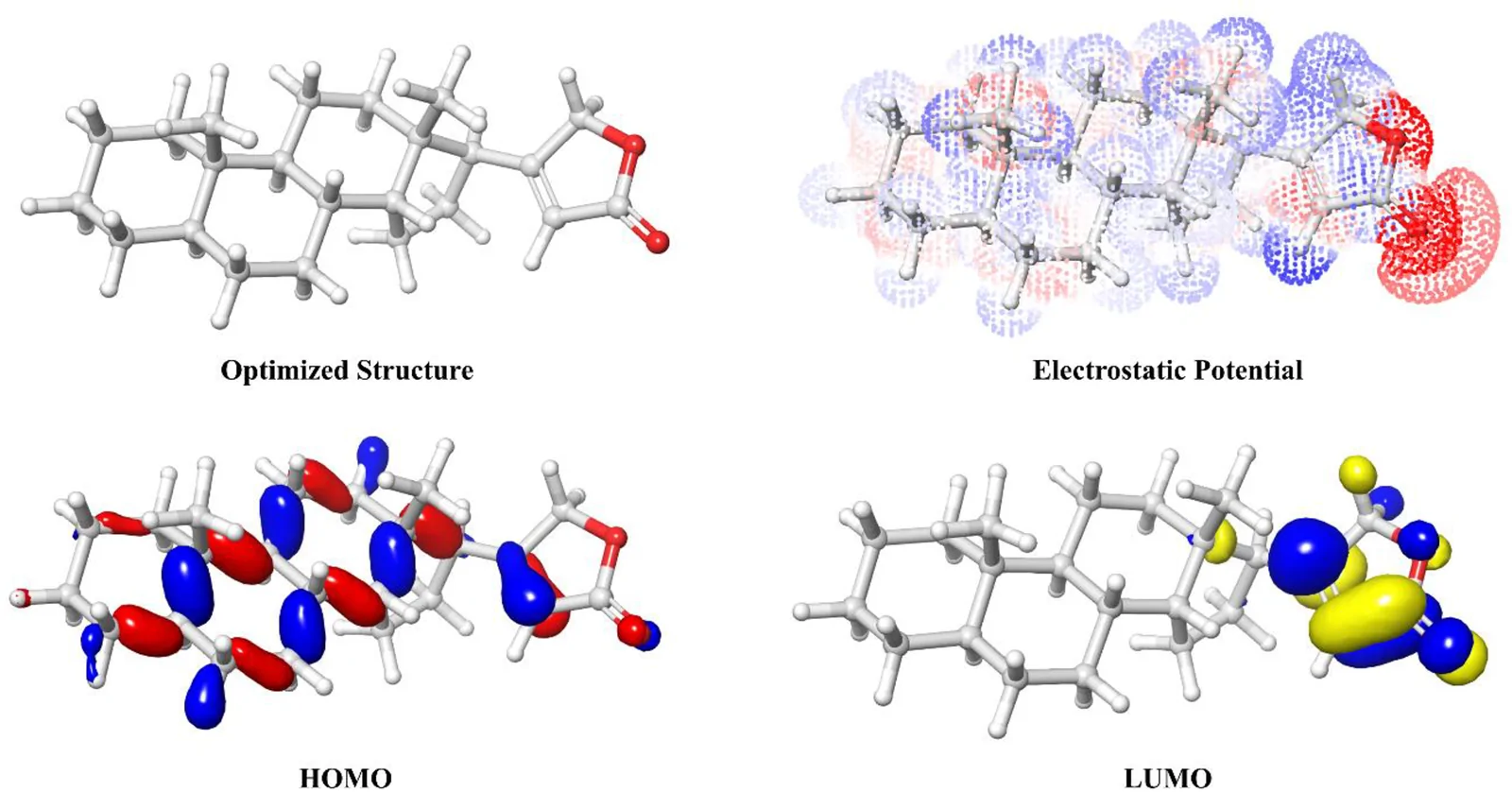
Optimized molecular structure, frontier molecular orbital (HOMO–LUMO) distributions, and molecular electrostatic potential (MEP) map of Cardenolide obtained through density functional theory (DFT) analysis.

Density functional theory (DFT) calculations were performed to investigate the electronic characteristics and reactive behavior of the selected lead ligand Cardenolide and to complement the molecular docking and molecular dynamics findings at the quantum-chemical level. DFT calculations were performed on the selected ligand to obtain the optimized geometry and electronic descriptors (Table 8). The geometric optimization generated a stable molecular conformation, which was subsequently used for the calculation of electronic properties. Fig 8 illustrates the optimized molecular structure, frontier molecular orbital (HOMO–LUMO) distributions, and molecular electrostatic potential (MEP) map of the ligand obtained through DFT analysis. The frontier molecular orbital (FMO) analysis was conducted to determine the HOMO and LUMO energy levels and evaluate the electronic characteristics of the compound. The HOMO and LUMO energies were observed at −7.123 eV and −1.221 eV, respectively, resulting in an energy gap (ΔE) of 5.902 eV. The calculated ionization potential (I) and electron affinity (A) were 7.123 eV and 1.221 eV, respectively. Additionally, the chemical potential (μ), hardness (η), electronegativity (χ), softness (σ), and electrophilicity index (ω) were determined as −4.172 eV, 2.951 eV, 4.172 eV, 0.169, and 2.948, respectively. Overall, the DFT analysis provided complementary electronic-level insight into the interaction behavior of Cardenolide and supported its prioritization as a potential EGFR-binding phytochemical. Importantly, these quantum chemical descriptors were interpreted as supportive physicochemical indicators rather than standalone evidence of inhibitory activity.

**Table 8:** Density functional theory (DFT)-calculated quantum chemical descriptors of the selected lead ligand Cardenolide.

Parameter	Value
HOMO (eV)	−7.123
LUMO (eV)	−1.221
Ionization potential, I (eV)	7.123
Electron affinity, A (eV)	1.221
Energy gap, ΔE (eV) = I-A	5.902
Chemical potential, μ (eV) = - I + A/2	−4.172
Hardness, η (eV) = I-A/2	2.951
Softness, σ = 1/n	0.169
Electronegativity, χ (eV) = I + A/2	4.172
Electrophilicity index, ω = μ2/2η	2.948

### Evolutionary conservation and hotspot distribution in the EGFR kinase domain

Conservation mapping showed that the predicted ligand-binding pocket is located within a functionally constrained region of the EGFR kinase domain (S8 Fig in S1 File). Several residues identified as major interaction sites in the docking and molecular dynamics analyses were moderately to highly conserved, including Phe699 (8), Val702 (9), Ala719 (9), Lys721 (9), Met769 (7), Leu820 (9), Thr830 (8), Asp831 (9), and Phe832 (9) that are present in Table 9. These findings indicate that the principal ligand-contact residues are concentrated within an evolutionarily preserved segment of the kinase pocket, supporting the structural plausibility of the predicted binding mode. In addition, hotspot positions corresponding to major clinically relevant EGFR mutation sites were found within the same kinase region, further reinforcing the biological and translational relevance of the identified binding pocket. Overall, this conservation-guided structural analysis provides an additional layer of support for the EGFR-Cardenolide interaction model identified in the present study.

**Table 9:** Conservation grades of key EGFR kinase-domain residues involved in ligand binding and hotspot mapping.

Residue	Evidence	ConSurf grade	Interpretation
Phe699	Docking + MD	8	Highly conserved pocket residue
Val702	Docking + MD	9	Highly conserved pocket residue
Ala719	Docking + MD	9	Highly conserved pocket residue
Lys721	Docking hotspot/ Structural hotspot	9	Highly conserved functional residue
Met769	Docking hotspot	7	Conserved pocket residue
Leu820	Docking + MD	9	Highly conserved hydrophobic anchor
Thr830	Docking + MD region	8	Highly conserved pocket residue
Asp831	Docking + MD	9	Highly conserved functional residue
Phe832	MD-supported pocket residue	9	Highly conserved pocket residue

### Clinical expression profile and pharmacogenomics context of EGFR in NSCLC

**Fig 9:**
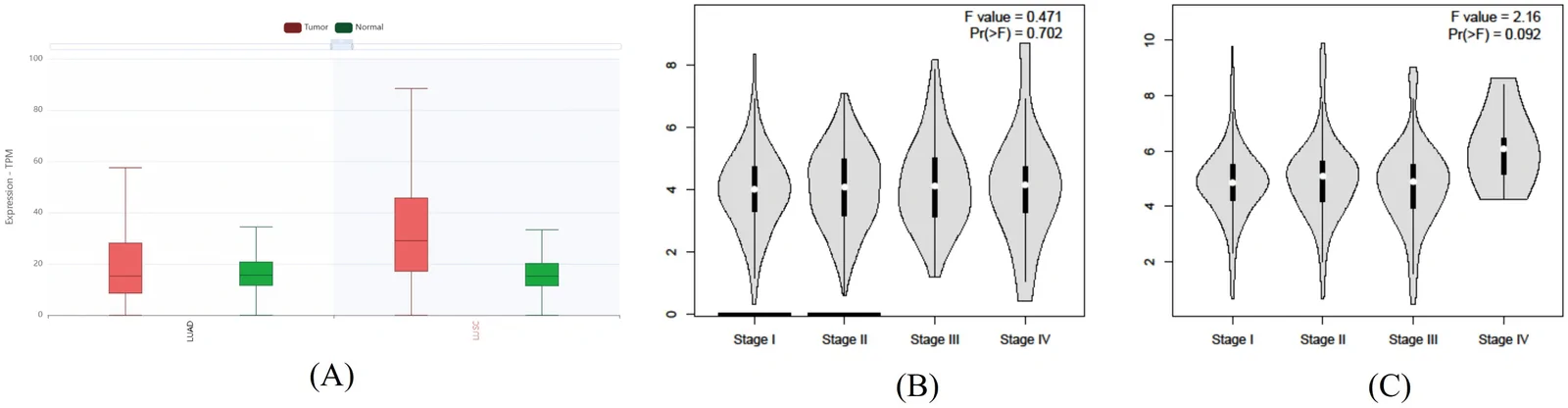
Clinical expression profile of EGFR in non-small-cell lung cancer. (A) Differential expression of EGFR in lung adenocarcinoma (LUAD) tumor and lung squamous cell carcinoma (LUSC) tumor and normal tissues. (B) Pathological stage-wise expression pattern of EGFR in LUAD. (C) Pathological stage-wise expression pattern of EGFR in LUSC.

**Fig 10:**
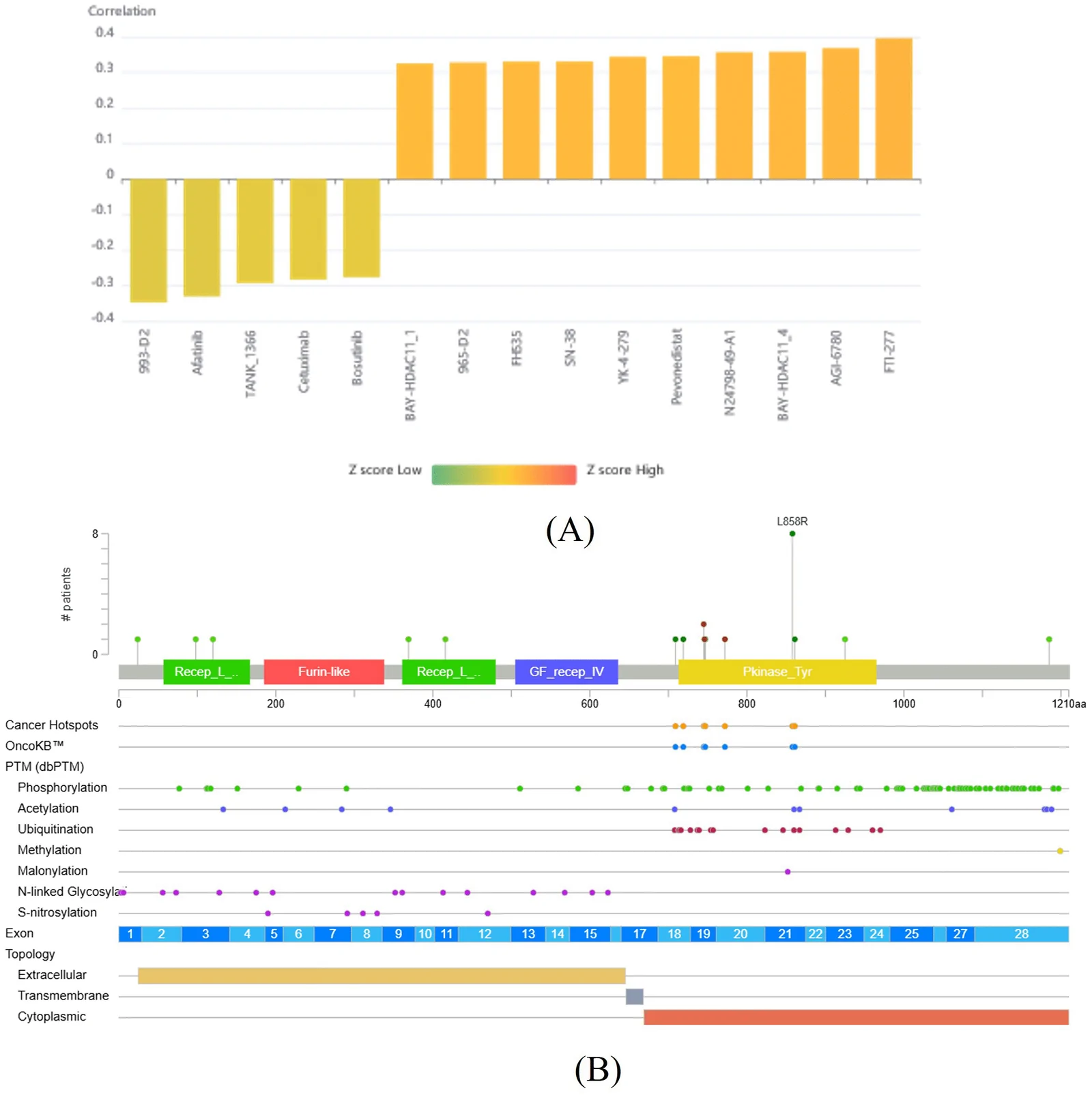
Pharmacogenomic and mutational landscape of EGFR in lung cancer. (A) Correlation between EGFR expression and drug sensitivity across lung cancer cell lines. (B) Positional distribution of EGFR mutations across the receptor structure in a lung adenocarcinoma cohort, showing hotspot alterations within the kinase region.

EGFR showed differential expression patterns between tumor and normal tissues in both LUAD and LUSC (Fig 9A). However, pathological stage analysis did not reveal marked stage-dependent variation in either cohort (Fig 9B-C), indicating that EGFR expression was not strongly associated with tumor stage in the present analysis. Drug-response screening further showed that EGFR expression was associated with distinct pharmacogenomic patterns across lung cancer cell lines (Fig 10A). Under the selected response metric, negative associations were observed for agents including afatinib, 993-D2, EphB4_9721, cetuximab, TANK_1366, trametinib, pelitinib, and RAF-9304, whereas positive associations were observed for compounds including BMN540215, C-75, A-484954, CD532, tricostatin A, parthenolide, sorafenib, PHA-665752, imatinib, and GNF-2. These results indicate that EGFR-linked drug-response patterns remain detectable in lung-derived cell-line datasets.

### Mutational landscape of EGFR in lung adenocarcinoma

Mutation profiling showed that EGFR alterations were distributed across several structural regions of the receptor, with a clear concentration of clinically relevant events in the kinase-domain segment (Fig 10A, B). The mutation-domain plot highlighted a prominent L858R hotspot within the tyrosine kinase region, together with additional lower-frequency alterations positioned nearby. This pattern is consistent with the recognized importance of kinase-domain mutations in EGFR-driven lung adenocarcinoma and reinforces the biological relevance of targeting this region in the present study. Overall, the cBioPortal-based mutation analysis provides a concise genomic context for the EGFR-focused structural findings and supports the therapeutic significance of the kinase-domain binding pocket identified in this work.

## Discussion

The present study combined molecular docking, molecular dynamics simulations, pharmacokinetic profiling, toxicity prediction, density functional theory (DFT), and cancer bioinformatics analyses to investigate the predicted EGFR-targeting potential of phytochemicals derived from *Adenium obesum* in the context of non-small-cell lung cancer (NSCLC). The computational workflow identified substantial differences among the investigated compounds in terms of predicted protein–ligand interaction characteristics, physicochemical properties, pharmacokinetic profiles, and structural behavior. Importantly, the findings should be interpreted as hypothesis-generating rather than as evidence of experimentally confirmed EGFR inhibition. Within this computational framework, Cardenolide was selected for subsequent detailed characterization because it demonstrated a comparatively balanced profile across the evaluated parameters, rather than because it exhibited the most favorable docking score. This distinction is important because docking scores alone cannot establish experimental binding affinity, enzymatic inhibition, therapeutic efficacy, or clinical applicability.

The prioritization of Cardenolide in the present study was based on an integrated interpretation of the computational results obtained during the initial screening process. Molecular docking identified several compounds with highly favorable predicted interactions with the EGFR kinase domain. Notably, SCHEMBL5933906 exhibited a slightly more negative docking score than Cardenolide against wild-type EGFR (−10.0 versus −9.9 kcal/mol) and also showed favorable predicted docking scores against the L858R/T790M and L858R/T790M/C797S mutation-containing EGFR structures (−10.0 and −10.8 kcal/mol, respectively). Echujin likewise demonstrated highly favorable predicted docking characteristics, with scores of −9.9, −9.2, and −9.6 kcal/mol against wild-type, L858R/T790M, and L858R/T790M/C797S EGFR, respectively. These findings indicate that Cardenolide was not selected because it possessed the strongest predicted docking performance among all investigated phytochemicals.

Instead, candidate selection considered the broader predicted physicochemical and pharmacokinetic characteristics of the compounds. SCHEMBL5933906 had a molecular weight of 764.94 g/mol, two predicted Lipinski rule violations, a topological polar surface area (TPSA) of 182.83 Å², a predicted bioavailability score of 0.17, and low predicted gastrointestinal absorption. Echujin similarly exhibited a molecular weight of 843.00 g/mol, three predicted Lipinski rule violations, a TPSA of 252.75 Å², a predicted bioavailability score of 0.17, and low predicted gastrointestinal absorption. In comparison, Cardenolide had a molecular weight of 342.00 g/mol, one predicted Lipinski rule violation, one rotatable bond, a TPSA of 26.30 Å², a predicted bioavailability score of 0.55, and high predicted gastrointestinal absorption. Its predicted human intestinal absorption was 98.343%, compared with 74.287% for SCHEMBL5933906 and 33.729% for Echujin. These differences suggested that Cardenolide possessed a comparatively more balanced predicted physicochemical and absorption profile under the criteria applied in the present computational workflow.

The toxicity predictions provided additional supportive, although not decisive, evidence for candidate selection. Cardenolide was not predicted to inhibit hERG, induce AMES mutagenicity, cause skin sensitization, or produce hepatotoxicity in the evaluated computational models. However, similar absence of predicted liabilities was also observed for SCHEMBL5933906 and Echujin across several of these endpoints. Therefore, toxicity predictions were considered as part of the overall assessment rather than as a distinguishing factor that established the superiority of Cardenolide. Taken together, the initial docking, drug-likeness, pharmacokinetic, and toxicity predictions supported the selection of Cardenolide for subsequent molecular dynamics investigation. Molecular dynamics simulation was subsequently performed to further characterize the temporal stability and interaction behavior of the selected Cardenolide–EGFR complex; it was not used as a comparative criterion for selecting Cardenolide over SCHEMBL5933906 or Echujin because these compounds were not subjected to molecular dynamics simulations in the present study. Accordingly, Cardenolide should be regarded as a computationally prioritized candidate for further investigation rather than as an experimentally validated or definitively superior EGFR inhibitor.

The predicted interaction profile of Cardenolide provided additional structural context for its computational prioritization. Clinically effective EGFR tyrosine kinase inhibitors (TKIs) commonly occupy the ATP-binding cleft and establish stabilizing interactions with residues located within the hinge region, catalytic loop, and activation loop, thereby interfering with ATP-dependent kinase signaling [51–53]. In the present study, Cardenolide was predicted to interact with residues including Val702, Ala719, Lys721, Met769, Asp831, and Phe832 within the EGFR kinase domain. These interactions provide a structurally plausible basis for the predicted binding mode; however, the present docking results cannot establish that these contacts are sufficient to produce enzymatic inhibition or that the compound acts through an ATP-competitive mechanism.

The predicted interaction pattern of Cardenolide also overlapped with residues exhibiting high evolutionary conservation. Conservation mapping indicated that several docking-associated residues, including Val702, Ala719, Met769, and Leu820, displayed high ConSurf conservation scores. Highly conserved residues may contribute to important structural or functional properties of proteins and can therefore represent biologically relevant regions for ligand interaction. The overlap between conserved residues and the predicted Cardenolide binding region supports the structural plausibility of the computationally predicted binding pose. Nevertheless, conservation alone does not demonstrate inhibitory activity or guarantee that a ligand targeting such residues will retain activity against clinically relevant EGFR variants.

The localization of the predicted binding region within the EGFR kinase domain is also relevant to the biology of NSCLC. Activating and resistance-associated EGFR alterations, including L858R, T790M, and C797S, are clinically important determinants of therapeutic response and acquired resistance [54,55]. The L858R alteration affects the conformational equilibrium of the kinase and promotes constitutive signaling, whereas T790M is associated with acquired resistance to earlier-generation EGFR TKIs through changes in ATP affinity and inhibitor recognition. C797S, in turn, can interfere with covalent inhibitor binding and contribute to resistance against third-generation EGFR TKIs. In the present study, the predicted Cardenolide binding region was located within the EGFR kinase domain in proximity to residues relevant to EGFR structure and function. To further examine whether predicted docking behavior varied in mutation-containing structural backgrounds, Cardenolide and the other investigated phytochemicals were additionally evaluated against EGFR structures containing L858R/T790M and L858R/T790M/C797S mutation combinations. Cardenolide retained favorable predicted docking scores in both mutation-containing structures (−8.6 and −8.5 kcal/mol, respectively), although these values were less negative than its predicted score against wild-type EGFR (−9.9 kcal/mol). These findings suggest that the predicted interaction profile of Cardenolide was maintained in the evaluated mutation-containing structural backgrounds, but they do not demonstrate that the compound can overcome EGFR TKI resistance or inhibit mutant EGFR enzymatically. Moreover, because the evaluated structures contain combinations of mutations, the present analysis cannot distinguish the independent effects of L858R, T790M, and C797S on ligand binding.

The reliability of the molecular docking protocol was further assessed by re-docking the co-crystallized EGFR ligand. The experimentally resolved ligand conformation was reproduced with an RMSD of 1.64 Å, which falls within the commonly used threshold of <2.0 Å for assessing the ability of a docking protocol to reproduce an experimentally observed binding pose [56]. The agreement between the native and re-docked conformations supports the technical reliability of the docking protocol under the specific computational conditions used in this study. Nevertheless, successful redocking validates the docking procedure rather than the biological activity of the newly investigated phytochemicals. Therefore, the predicted interactions of Cardenolide should be interpreted as structurally plausible computational hypotheses requiring experimental validation.

The dynamic behavior observed during molecular dynamics simulations provided additional information regarding the temporal stability of the selected Cardenolide–EGFR complex. Unlike static docking, molecular dynamics simulations allow the behavior of protein–ligand complexes to be examined over time and can provide insights into conformational adaptation, ligand persistence, and residue-level fluctuations under simulated conditions [57,58]. Throughout the simulation period, the Cardenolide-bound complex maintained overall structural integrity without evidence of substantial destabilization of the receptor. The observed behavior, together with the maintenance of interactions within the binding region, suggests that the predicted Cardenolide–EGFR complex remained dynamically compatible with the kinase domain during the simulated trajectory.

The observed suppression of fluctuations in functionally relevant regions and preservation of secondary structural elements further suggest that Cardenolide binding did not induce major disruption of the global receptor architecture. However, these observations should not be interpreted as direct evidence of inhibitory activity. A stable protein–ligand complex in a molecular dynamics simulation indicates that the modeled complex is computationally compatible under the simulated conditions, but it does not establish experimentally measurable binding affinity or enzymatic inhibition.

Principal component analysis (PCA) provided additional information regarding the dominant collective motions of the simulated system. The first two principal components accounted for 52.3% of the total system motion, indicating that a substantial proportion of the observed conformational variability was represented by a limited number of dominant collective motions. The formation of distinct yet relatively compact conformational clusters suggested that the receptor–ligand system sampled a restricted set of conformational states during the simulation. The absence of highly dispersed trajectory distributions further indicated that the Cardenolide-bound complex did not undergo pronounced global structural destabilization during the simulated period. Collectively, these findings support the computational stability of the modeled Cardenolide–EGFR complex and provide a basis for further experimental investigation, while recognizing that molecular dynamics simulations remain predictive and cannot substitute for biochemical validation.

The predicted physicochemical and pharmacokinetic characteristics of Cardenolide contributed substantially to its computational prioritization. Successful drug development generally requires an appropriate balance among molecular size, lipophilicity, polarity, and hydrogen-bonding properties to facilitate adequate absorption, distribution, and exposure [59,60]. In the present study, Cardenolide exhibited a molecular weight of 342.00 g/mol, one predicted Lipinski rule violation, one rotatable bond, and a relatively low TPSA of 26.30 Å². It was also predicted to have high gastrointestinal absorption and a human intestinal absorption value of 98.343%. These characteristics were comparatively more favorable than those predicted for several larger, highly glycosylated phytochemicals investigated in the present study.

The relatively lower molecular size and polarity of Cardenolide compared with several glycosylated compounds may reduce some of the physicochemical limitations commonly associated with large, highly polar natural products [61]. In contrast, extensive glycosylation can increase molecular weight, polarity, and hydrogen-bonding capacity, potentially influencing passive membrane transport and oral exposure [62]. Several compounds with favorable docking scores exhibited multiple predicted Lipinski rule violations, high molecular weights, elevated topological polar surface areas (TPSA), and low predicted gastrointestinal absorption. For example, SCHEMBL5933906 and Echujin, which demonstrated highly favorable predicted EGFR docking characteristics, exhibited two and three predicted Lipinski rule violations, respectively, together with low predicted gastrointestinal absorption. Similarly, several glycosylated phytochemicals, including Obebioside A, Obetrioside B, Obebioside B, Hongheloside C, Obebioside C, and Obetrioside A, showed multiple predicted Lipinski violations and low gastrointestinal absorption. These findings highlight an important limitation of docking-based prioritization: strong predicted protein–ligand interactions do not necessarily correspond to favorable physicochemical or pharmacokinetic properties required for successful drug development.

Nevertheless, the predicted physicochemical profile of Cardenolide should not be considered uniformly favorable. Its consensus Log Po/w value of 5.22 indicates relatively high predicted lipophilicity, which may introduce potential pharmacokinetic liabilities that require experimental assessment. Therefore, the significance of the ADME findings is best understood in terms of an overall balance of predicted properties rather than as evidence of an unequivocally optimal drug-like profile. Furthermore, all absorption, distribution, metabolism, and pharmacokinetic characteristics reported in this study are computational predictions and require experimental confirmation.

Safety prediction analyses provided additional context for the computational prioritization of Cardenolide. Cardiac glycosides and structurally related steroidal compounds have been investigated for anticancer activity, but some members of these chemical classes may exhibit cardiotoxicity, hepatotoxicity, or other systemic adverse effects [63–65]. In the present study, Cardenolide was not predicted to inhibit hERG or to exhibit AMES mutagenicity, skin sensitization, or hepatotoxicity in the evaluated computational models. However, similar favorable predictions were observed for several other investigated compounds, and therefore these results do not establish a unique safety advantage for Cardenolide. Rather, the absence of predicted liabilities across the evaluated endpoints provided supportive evidence for its further computational investigation. As computational toxicity predictions cannot replace experimental safety assessment, these findings should be regarded as preliminary indicators requiring subsequent validation.

DFT calculations were performed to further characterize the electronic properties of Cardenolide following geometry optimization. The optimized structure indicated a stable molecular conformation suitable for electronic analysis. The calculated HOMO and LUMO energies were −7.123 eV and −1.221 eV, respectively, corresponding to an energy gap of 5.902 eV. These values indicate a relatively stable electronic structure with limited predicted chemical reactivity under the applied computational framework. The calculated ionization potential (7.123 eV) and electron affinity (1.221 eV) further supported the predicted electronic stability of the optimized ligand.

The calculated global reactivity descriptors, including a chemical potential of −4.172 eV and hardness of 2.951 eV, suggested an electronically stable system, whereas the electrophilicity index of 2.948 indicated moderate electron-accepting characteristics. The molecular electrostatic potential (MEP) map further revealed distinct electron-rich and electron-deficient regions that may contribute to electrostatic complementarity and potential hydrogen-bonding interactions with biological targets. These results provide additional molecular-level information regarding the electronic characteristics of Cardenolide. However, DFT-derived descriptors do not directly establish EGFR binding or inhibitory activity and should therefore be considered complementary to, rather than independent confirmation of, the docking and molecular dynamics findings.

Clinical expression and pharmacogenomic analyses provide additional context for the therapeutic importance of EGFR in NSCLC. EGFR dysregulation and aberrant signaling are implicated in lung adenocarcinoma (LUAD) and lung squamous cell carcinoma (LUSC), where persistent receptor activation can contribute to tumor proliferation, survival, invasion, and therapeutic resistance. In the present bioinformatics analysis, EGFR expression remained elevated across pathological stages, suggesting that EGFR-associated signaling may remain biologically relevant throughout disease progression. Previous studies have also indicated continued dependence on EGFR-related signaling in subsets of tumors following treatment failure, supporting the importance of the EGFR pathway as a therapeutic target [66,67].

The concentration of clinically important mutations within the EGFR kinase domain further emphasizes the relevance of evaluating candidate compounds against mutation-containing structures. The additional docking analysis performed against L858R/T790M and L858R/T790M/C797S EGFR structures indicated compound-dependent variation in predicted docking behavior across the investigated phytochemicals. Some compounds retained or even exhibited more negative docking scores in mutation-containing structures, whereas others showed less negative scores relative to wild-type EGFR. These observations indicate that mutation-associated structural differences may influence predicted ligand interaction patterns in a compound-dependent manner. However, docking outcomes alone cannot determine whether these compounds retain biochemical activity against mutant EGFR or whether they can overcome established mechanisms of EGFR TKI resistance. Experimental enzymatic and cellular studies using relevant EGFR variants will therefore be necessary to establish the biological significance of these computational observations.

Previous studies have investigated several naturally occurring compounds in relation to EGFR signaling and EGFR-targeted anticancer activity. Representative examples include curcumin, epigallocatechin-3-gallate (EGCG), quercetin, resveratrol, berberine, and genistein, which have been reported to influence EGFR-associated signaling or tyrosine kinase activity in different experimental and computational contexts [68–70]. Among these compounds, curcumin have been investigated in relation to EGFR tyrosine kinase activity [71], whereas EGCG, quercetin, resveratrol, and berberine have been associated with modulation of EGFR-related signaling pathways and downstream cancer-related processes [72–75]. In comparison with these previously investigated natural products, the present study extends the exploration of plant-derived EGFR-targeting candidates to phytochemicals from *Adenium obesum*. To the best of our knowledge, the present work provides the first systematic computational assessment of 16 *A. obesum* phytochemicals against EGFR using an integrated workflow incorporating molecular docking, drug-likeness and ADME prediction, toxicity profiling, molecular dynamics simulation, and evaluation against mutation-containing EGFR structures. Thus, the present findings expand the computational investigation of natural-product-based EGFR-targeting candidates and provide testable hypotheses for subsequent experimental validation.

Taken together, the present computational investigation identified Cardenolide as a computationally prioritized candidate among the evaluated *Adenium obesum* phytochemicals based on the overall balance of predicted EGFR-binding characteristics, physicochemical properties, pharmacokinetic parameters, toxicity-related predictions, and subsequent molecular dynamics characterization. Importantly, this prioritization does not imply that Cardenolide is superior to all other investigated compounds in every computational parameter. In particular, SCHEMBL5933906 and Echujin exhibited comparable or more favorable predicted docking scores, while Cardenolide was prioritized because of its comparatively more balanced predicted drug-likeness and absorption characteristics. Molecular dynamics simulations subsequently provided additional information regarding the temporal stability of the selected Cardenolide–EGFR complex but were not used to establish comparative superiority over compounds that were not subjected to molecular dynamics simulations.

The integration of evolutionary conservation analysis, mutation-containing EGFR docking, clinical expression profiling, ADME prediction, toxicity assessment, DFT analysis, and molecular dynamics simulation provides a broader computational characterization than docking alone. The predicted interaction of Cardenolide with conserved regions of the EGFR kinase domain, together with its retained predicted docking compatibility in the evaluated mutation-containing structures, provides a hypothesis for further investigation of its potential relevance to EGFR-driven NSCLC. Nevertheless, the present study remains entirely computational, and none of the findings demonstrate experimentally confirmed EGFR binding, enzymatic inhibition, anticancer efficacy, or the ability to overcome clinically established EGFR resistance mechanisms. Therefore, Cardenolide should be considered a computationally prioritized, hypothesis-generating candidate rather than a confirmed EGFR inhibitor. Biochemical assays measuring EGFR kinase inhibition, cellular studies in EGFR-driven NSCLC models, and subsequent in vivo investigations will be required to determine whether the computationally predicted characteristics of Cardenolide translate into measurable biological activity and therapeutic potential.

## Conclusion

The present study provides a comprehensive computational investigation of Adenium obesum phytochemicals as candidate compounds for EGFR targeting in the context of non-small-cell lung cancer (NSCLC). By integrating bioactivity prediction, molecular docking, interaction profiling, pharmacokinetic and toxicity assessment, molecular dynamics simulations, density functional theory (DFT), and cancer bioinformatics analyses, this work extends beyond conventional virtual screening approaches to establish a biological and clinically contextualized framework for lead identification.

Among the sixteen phytochemicals evaluated, several compounds demonstrated favorable predicted antineoplastic activity and strong binding affinity toward the EGFR kinase domain. However, substantial differences emerged when binding stability, pharmacokinetic feasibility, dynamic behavior, and biological relevance were collectively considered. Notably, Cardenolide exhibited the most balanced overall profile, combining stable target engagement, favorable drug-likeness characteristics, acceptable predicted safety, and persistent conformational stability during 100-ns molecular dynamics simulations. The ligand maintained a cooperative interaction network involving functionally important residues within the hinge region, catalytic loop, and activation loop of EGFR, closely resembling the binding characteristics of established ATP-competitive kinase inhibitors. Importantly, the integration of evolutionary conservation and cancer bioinformatics analyses substantially strengthened the translational significance of the computational findings. The predicted binding interactions of Cardenolide localized within highly conserved and clinically relevant regions of the EGFR kinase domain, including residues associated with structural stability and kinase regulation. Several interacting residues overlapped with evolutionarily constrained hotspot regions adjacent to clinically important mutation sites implicated in NSCLC pathogenesis and therapeutic resistance. These findings suggest that the identified scaffold targets biologically indispensable regions of EGFR rather than nonspecific surface interactions, thereby reinforcing the mechanistic plausibility of the proposed inhibitory activity. Pharmacokinetic and toxicity analyses further distinguished smaller aglycone-type compounds from highly glycosylated cardenolides. While multiple glycosylated phytochemicals demonstrated strong docking affinities, their elevated molecular size and polarity suggested less favorable oral bioavailability and membrane permeability. In contrast, Cardenolide displayed a more balanced physicochemical profile compatible with improved pharmacokinetic feasibility. Toxicity predictions additionally indicated minimal risks of major cardiotoxic, hepatotoxic, mutagenic, or adverse drug–drug interaction liabilities, supporting its relative developability as a therapeutic lead candidate. The molecular dynamics and PCA analyses collectively demonstrated that the EGFR–Cardenolide complex remained structurally stable while exploring a limited and well-defined conformational landscape. Furthermore, DFT calculations revealed a stable electronic framework characterized by moderate chemical reactivity and favorable charge distribution capable of supporting electrostatic and hydrogen-bonding interactions with the receptor. Together, these findings support the formation of a stable and biologically relevant protein–ligand complex. Overall, this study provides the first integrated computational evidence supporting *Adenium obesum* as a promising and underexplored source of EGFR-targeting phytochemicals for NSCLC. More importantly, the study highlights the value of combining structural bioinformatics, evolutionary conservation analysis, molecular simulation, and cancer genomics with ethnopharmacological resources to improve the mechanistic interpretation and translational relevance of computational drug discovery. Among the investigated compounds, Cardenolide emerged as the most promising lead scaffold for future EGFR-targeted anticancer development.

Nevertheless, several limitations should be acknowledged. All findings remain predictive and computational in nature, and no experimental validation was performed in the present study. Although the integrated analyses collectively provide high-confidence support for the EGFR-targeting potential of Cardenolide, these results should be interpreted as hypothesis-generating rather than definitive evidence of therapeutic efficacy. Experimental validation through in vitro kinase inhibition assays, NSCLC cell-line cytotoxicity studies, resistance-associated mutation models, and subsequent in vivo pharmacological evaluations will be essential to confirm the biological activity, selectivity, and safety of the identified compounds. Future studies may additionally investigate structure–activity relationships, semisynthetic optimization strategies, and the potential effectiveness of Cardenolide against resistant EGFR variants. Such studies will be critical for determining the clinical relevance and therapeutic potential of Cardenolide and related *Adenium obesum* phytochemicals as prospective EGFR-targeted interventions for non-small-cell lung cancer. Overall, the present study highlights *Adenium obesum* as a promising and underexplored source of EGFR-targeting phytochemicals and demonstrates the value of integrating structural biology, molecular simulation, evolutionary bioinformatics, and cancer genomics to accelerate rational anticancer drug discovery.

## Supporting information

S1 File Supplementary file contains figures and tables providing detailed information on EGFR structural characterization and validation, *Adenium obesum* phytochemicals and reference EGFR inhibitors, molecular docking validation and protein–ligand interactions, docking poses, secondary structure element analysis, ligand RMSF and torsional analyses, time-dependent ligand property analysis, and evolutionary conservation-guided mapping of ligand-contact residues and clinical hotspot sites in the EGFR kinase domain. (DOCX)
