# Supplementary material for: Computational identification and evaluation of Adenium obesum phytochemicals as potential EGFR inhibitors for targeted therapy against non-small-cell lung cancer
Source: PLoS One. 2026 Aug 31;21(8):e0357096. doi: 10.1371/journal.pone.0357096 (PMC13529004; doi:10.1371/journal.pone.0357096)
Supplement: Supplementary file 1 [file pone.0357096.s001.docx]

**S1 Fig. 3D protein structure of EGFR (1M17, 6LUD, 4I22).**

**S2 Fig. Validation of the molecular docking protocol through superimposition of the crystallographic native ligand (green) and the re-docked conformation (pale lavender) within the EGFR active site. The strong overlap between the two poses demonstrated accurate reproduction of the experimentally observed binding orientation with an RMSD value of 1.64 Å.**

**Hongheloside A with EGFR**

**SCHEMBL5933906 with EGFR**

**Obebioside A with EGFR**

**Strospeside with EGFR**

**Obeside C with EGFR**

**Isokaempferide with EGFR**

**Obetrioside B with EGFR**

**Obebioside B with EGFR**

**Cardenolide with EGFR**

**Echujin with EGFR**

**Hongheloside C with EGFR**

**Obebioside C with EGFR**

**Somalin with EGFR**

**Obetrioside A with EGFR**

**Neritaloside with EGFR**

**Mono-O-acetylacoschimperoside P with EGFR**

**S3 Fig. Molecular docking poses of selected phytochemicals with EGFR (PDB ID: 1M17). The 3D representations illustrate ligand orientation within the binding cavity, while the 2D interaction maps depict conventional and non-conventional hydrogen bonds, as well as hydrophobic interactions (π-alkyl, π-sigma, and alkyl) with key active-site residues.**

| % Helix | % Strand | % Total SSE |
| --- | --- | --- |
| 29.71 | 16.35 | 46.06 |

**S4 Fig. Secondary structure element (SSE) analysis.**

Protein secondary structure elements (SSE) like alpha-helices and beta-strands are monitored throughout the simulation. The plot above reports SSE distribution by residue index throughout the protein structure. The plot below summarizes the SSE composition for each trajectory frame over the course of the simulation, and the plot at the bottom monitors each residue and its SSE assignment over time.

**S5 Fig. Ligand RMSF Analysis**

Ligand RMSF analysis revealed low atomic fluctuations across most heavy atoms, indicating that the phytochemical adopts a well-defined bound conformation within the EGFR active site. Slightly elevated fluctuations were confined to peripheral substituents, which are likely solvent-exposed and do not contribute critically to binding stability. When analyzed relative to the protein backbone, ligand fluctuations remained minimal, confirming that internal flexibility rather than positional instability dominates ligand motion. This suggests that the ligand achieves an optimal balance between rigidity (for binding specificity) and flexibility (for adaptive fit), a hallmark of drug-like behavior.

**S6 Fig. Ligand torsional analysis**

The ligand torsion profile demonstrated that the ligand predominantly occupied low-energy conformational states while bound to EGFR, indicating minimal conformational strain. Such behavior suggests that the ligand’s bioactive conformation is energetically favorable and does not require significant distortion to maintain binding a hallmark of efficient and selective inhibitors.

**S7 Fig. Time-dependent analysis of ligand properties**

Ligand RMSD: Root means square deviation of a ligand with respect to the reference conformation (typically the first frame is used as the reference, and it is regarded as time *t*=0).

Radius of Gyration (rGyr): Measures the 'extendedness' of a ligand and is equivalent to its principal moment of inertia.

Intramolecular Hydrogen Bonds (intraHB): Number of internal hydrogen bonds (HB) within a ligand molecule.

Molecular Surface Area (MolSA): Molecular surface calculation with 1.4 Å probe radius. This value is equivalent to a van der Waals surface area.

Solvent Accessible Surface Area (SASA): Surface area of a molecule accessible by a water molecule.

Polar Surface Area (PSA): Solvent accessible surface area in a molecule contributed only by oxygen and nitrogen atoms.

Time-dependent analysis of ligand physicochemical properties further corroborated binding stability. The radius of gyration remained essentially constant, indicating that the ligand neither collapsed nor excessively extended during the simulation. Solvent-accessible surface area (SASA) and polar surface area (PSA) exhibited only minor fluctuations, reflecting consistent burial of hydrophobic moieties and stable exposure of polar functional groups.

**S8 Fig. Evolutionary conservation-guided mapping of key ligand-contact residues and clinical hotspot sites in the EGFR kinase domain (PDB: 1M17). (A) Overall structure highlighting ligand-contact residues (blue) and hotspot mutation sites (red). (B) Close-up of the ATP-binding pocket showing key stabilizing residues, indicating a conserved functional binding region.**

**S1 Table. Structural quality parameters of EGFR proteins (PDB ID: 1M17, 4I22, 6LUD).**

| **Parameter** | **EGFR Wild-Type** | **EGFR L858R/T790M** | **EGFR L858R/T790M/C797S** |
| --- | --- | --- | --- |
| **PDB ID** | 1M17 | 4I22 | 6LUD |
| **Organism** | *Homo sapiens* | *Homo sapiens* | *Homo sapiens* |
| **Molecule** | Epidermal Growth Factor Receptor (EGFR) | Epidermal Growth Factor Receptor (EGFR) | Epidermal Growth Factor Receptor (EGFR) |
| **Structural Background** | Wild-type | L858R/T790M | L858R/T790M/C797S |
| **Resolution (Å)** | 2.60 | 1.71 | 2.05 |
| **R-Value Free** | 0.295 | 0.224 | 0.222 |
| **R-Value Work** | 0.251 | 0.197 | 0.206 |

**S2 Table. Adenium obesum phytochemicals and standard EGFR inhibitors 2D chemical structures**

| Compound No. | Compound Name | Pubchem ID | 2D Chemical Structure |
| --- | --- | --- | --- |
| L01 | Hongheloside A | 56841096 |  |
| L02 | SCHEMBL5933906 | 6281 |  |
| L03 | Obebioside A | 23259011 |  |
| L04 | Strospeside | 21636336 |  |
| L05 | Obeside C | 23259009 |  |
| L06 | Obetrioside B | 21636338 |  |
| L07 | Obebioside B | 21636337 |  |
| L08 | Echujin | 44566920 |  |
| L09 | Hongheloside C | 71301114 |  |
| L10 | Obebioside C | 23259012 |  |
| L11 | Somalin | 12310598 |  |
| L12 | Obeside C | 23259014 |  |
| L13 | Neritaloside | 44566654 |  |
| L14 | Isokaempferide | 5280862 |  |
| L15 | Cardenolide | 53957771 |  |
| L16 | Mono-O-acetylacoschimperoside P | 91809626 |  |
| L17 | Osimertinib | 71496458 |  |
| L18 | Afatinib | 10184653 |  |
| L19 | Erlotinib | 176870 |  |
| L20 | Lazertinib | 121269225 |  |

**S3 Table. Validation of the molecular docking protocol using re-docking analysis of the co-crystallized EGFR ligand.**

| **Parameter** | **Description/Value** |
| --- | --- |
| Target protein & PDB ID | EGFR tyrosine kinase receptor (1M17) |
| Validation method | Re-docking of co-crystallized ligand |
| Docking software | AutoDock Vina implemented in PyRx |
| Binding region | ATP-binding pocket |
| Best docking affinity | −6.5 kcal/mol |
| RMSD value | 1.64 Å |
| Validation criterion | RMSD < 2.0 Å [52] |
| Validation outcome | Successful |

**S4 Table. Protein–ligand interaction analysis of selected compounds with EGFR (PDB ID: 1M17), showing conventional and carbon hydrogen bonds, π-alkyl/π-sigma interactions, and other non-bonded contacts with critical active-site residues.**

| **Name Of the Ligand** | **Name** | **Distance (Å)** | **Bond Catagory** | **Bond Type** |
| --- | --- | --- | --- | --- |
| Hongheloside A | A:THR766 | 2.41737 | Hydrogen Bond | Conventional Hydrogen Bond |
|  | A:THR830 | 2.12114 | Hydrogen Bond | Conventional Hydrogen Bond |
|  | A:GLY772 | 3.52892 | Hydrogen Bond | Carbon Hydrogen Bond |
|  | A:ARG817 | 3.5831 | Hydrogen Bond | Carbon Hydrogen Bond |
|  | A:ASP776 | 3.50585 | Hydrogen Bond | Carbon Hydrogen Bond |
|  | A:ASP776 | 3.69557 | Hydrogen Bond | Carbon Hydrogen Bond |
|  | A:CYS773 | 5.35336 | Hydrophobic | Alkyl |
| SCHEMBL5933906 | A:LYS721 | 5.18532 | Hydrophobic | Alkyl |
|  | A:MET742 | 5.36864 | Hydrophobic | Alkyl |
| Obebioside A | A:LEU694 | 2.73779 | Hydrogen Bond | Conventional Hydrogen Bond |
|  | A:ARG817 | 1.93601 | Hydrogen Bond | Conventional Hydrogen Bond |
|  | A:PHE699 | 5.37628 | Hydrophobic | Pi-Alkyl |
| Strospeside | A:LYS721 | 2.57757 | Hydrogen Bond | Conventional Hydrogen Bond |
|  | A:VAL702 | 5.08545 | Hydrophobic | Alkyl |
|  | A:CYS773 | 5.05942 | Hydrophobic | Alkyl |
|  | A:LEU820 | 5.40745 | Hydrophobic | Alkyl |
|  | A:PHE699 | 4.79507 | Hydrophobic | Pi-Alkyl |
|  | A:PHE699 | 4.8459 | Hydrophobic | Pi-Alkyl |
| Obeside C | A:ASP813 | 1.76039 | Hydrogen Bond | Conventional Hydrogen Bond |
|  | A:GLY772 | 3.40478 | Hydrogen Bond | Carbon Hydrogen Bond |
|  | A:ARG817 | 5.04547 | Hydrophobic | Alkyl |
| Isokaempferide | A:THR830 | 2.70639 | Hydrogen Bond | Conventional Hydrogen Bond |
|  | A:ASP831 | 3.34798 | Hydrogen Bond | Carbon Hydrogen Bond |
|  | A:VAL702 | 4.6497 | Hydrophobic | Pi-Alkyl |
|  | A:ALA719 | 4.88177 | Hydrophobic | Pi-Alkyl |
|  | A:LYS721 | 5.3646 | Hydrophobic | Pi-Alkyl |
|  | A:LEU820 | 5.41183 | Hydrophobic | Pi-Alkyl |
|  | A:VAL702 | 5.29626 | Hydrophobic | Pi-Alkyl |
|  | A:ALA719 | 4.70279 | Hydrophobic | Pi-Alkyl |
|  | A:LYS721 | 4.09913 | Hydrophobic | Pi-Alkyl |
|  | A:LEU694 | 5.01419 | Hydrophobic | Pi-Alkyl |
|  | A:VAL702 | 5.02567 | Hydrophobic | Pi-Alkyl |
|  | A:LEU820 | 5.07249 | Hydrophobic | Pi-Alkyl |
| Obetrioside B | A:MET769 | 2.31593 | Hydrogen Bond | Conventional Hydrogen Bond |
|  | A:CYS773 | 2.27192 | Hydrogen Bond | Conventional Hydrogen Bond |
|  | A:ASP831 | 1.97495 | Hydrogen Bond | Conventional Hydrogen Bond |
|  | A:LYS851 | 2.867 | Hydrogen Bond | Conventional Hydrogen Bond |
|  | A:GLY833 | 3.42304 | Hydrogen Bond | Carbon Hydrogen Bond |
|  | A:GLU738 | 3.41363 | Hydrogen Bond | Carbon Hydrogen Bond |
|  | A:PHE699 | 5.15567 | Hydrophobic | Pi-Alkyl |
| Obebioside B | A:GLU734 | 2.30584 | Hydrogen Bond | Conventional Hydrogen Bond |
|  | A:ASP831 | 3.02141 | Hydrogen Bond | Carbon Hydrogen Bond |
|  | A:PHE699 | 3.47143 | Hydrogen Bond | Carbon Hydrogen Bond |
|  | A:PHE699 | 5.22671 | Hydrophobic | Pi-Alkyl |
| Cardenolide | A:ASP831 | 2.1615 | Hydrogen Bond | Conventional Hydrogen Bond |
|  | A:LEU694 | 5.22069 | Hydrophobic | Alkyl |
|  | A:LEU694 | 3.73304 | Hydrophobic | Alkyl |
|  | A:VAL702 | 5.27966 | Hydrophobic | Alkyl |
|  | A:VAL702 | 3.98429 | Hydrophobic | Alkyl |
|  | A:ALA719 | 4.98923 | Hydrophobic | Alkyl |
|  | A:ALA719 | 4.88268 | Hydrophobic | Alkyl |
|  | A:LYS721 | 4.86304 | Hydrophobic | Alkyl |
|  | A:LEU820 | 4.71337 | Hydrophobic | Alkyl |
| Echujin | A:MET769 | 2.03445 | Hydrogen Bond | Conventional Hydrogen Bond |
|  | A:GLU738 | 2.76388 | Hydrogen Bond | Conventional Hydrogen Bond |
|  | A:GLU734 | 2.53354 | Hydrogen Bond | Conventional Hydrogen Bond |
|  | A:GLU738 | 2.46956 | Hydrogen Bond | Conventional Hydrogen Bond |
|  | A:GLU734 | 2.23301 | Hydrogen Bond | Conventional Hydrogen Bond |
|  | A:LYS851 | 2.87917 | Hydrogen Bond | Conventional Hydrogen Bond |
|  | A:LYS851 | 1.88851 | Hydrogen Bond | Conventional Hydrogen Bond |
|  | A:ARG817 | 3.74555 | Hydrogen Bond | Carbon Hydrogen Bond |
|  | A:ARG817 | 5.35494 | Hydrophobic | Alkyl |
| Hongheloside C | A:LYS721 | 2.68767 | Hydrogen Bond | Conventional Hydrogen Bond |
|  | A:GLU738 | 2.27001 | Hydrogen Bond | Conventional Hydrogen Bond |
|  | A:GLU734 | 3.06235 | Hydrogen Bond | Conventional Hydrogen Bond |
|  | A:GLU738 | 2.25387 | Hydrogen Bond | Conventional Hydrogen Bond |
|  | A:GLU734 | 3.3682 | Hydrogen Bond | Carbon Hydrogen Bond |
|  | A:PHE699 | 3.9939 | Hydrophobic | Pi-Sigma |
|  | A:PHE699 | 3.79772 | Hydrophobic | Pi-Sigma |
|  | A:ARG817 | 4.92023 | Hydrophobic | Alkyl |
| Obebioside C | A:LEU694 | 2.79535 | Hydrogen Bond | Conventional Hydrogen Bond |
|  | A:ASP831 | 2.53835 | Hydrogen Bond | Conventional Hydrogen Bond |
|  | A:ARG817 | 1.93528 | Hydrogen Bond | Conventional Hydrogen Bond |
|  | A:PHE699 | 5.32942 | Hydrophobic | Pi-Alkyl |
| Somalin | A:ARG817 | 4.95303 | Hydrophobic | Alkyl |
|  | A:ARG817 | 2.92489 | Hydrogen Bond | Conventional Hydrogen Bond |
|  | A:LEU694 | 3.97881 | Hydrophobic | Alkyl |
| Obeside C | A:LYS721 | 2.04395 | Hydrogen Bond | Conventional Hydrogen Bond |
|  | A:MET769 | 2.07047 | Hydrogen Bond | Conventional Hydrogen Bond |
|  | A:PHE699 | 2.409 | Hydrogen Bond | Conventional Hydrogen Bond |
|  | A:PHE699 | 2.77819 | Hydrogen Bond | Conventional Hydrogen Bond |
|  | A:ASP831 | 2.13181 | Hydrogen Bond | Conventional Hydrogen Bond |
|  | A:ASP831 | 2.04229 | Hydrogen Bond | Conventional Hydrogen Bond |
|  | A:GLY833 | 3.68316 | Hydrogen Bond | Carbon Hydrogen Bond |
|  | A:LYS851 | 3.4637 | Hydrogen Bond | Carbon Hydrogen Bond |
|  | A:PHE699 | 5.38326 | Hydrophobic | Pi-Alkyl |
| Neritaloside | A:THR766 | 2.29839 | Hydrogen Bond | Conventional Hydrogen Bond |
|  | A:THR830 | 1.99325 | Hydrogen Bond | Conventional Hydrogen Bond |
|  | A:ASP831 | 2.38893 | Hydrogen Bond | Conventional Hydrogen Bond |
| Mono-O-acetylacoschimperoside P | A:ARG817 | 2.68496 | Hydrogen Bond | Conventional Hydrogen Bond |
|  | A:ARG817 | 5.45321 | Hydrophobic | Alkyl |
