# Supplementary material for: Computational identification and evaluation of Adenium obesum phytochemicals as potential EGFR inhibitors for targeted therapy against non-small-cell lung cancer
Source: PLoS One. 2026 Aug 31;21(8):e0357096. doi: 10.1371/journal.pone.0357096 (PMC13529004; doi:10.1371/journal.pone.0357096)
Supplement: Supplementary file 2 [file pone.0357096.s002.docx]

**Response to the Editor and Reviewers**

Manuscript ID: **PONE-D-26-27333**

Title: **Computational Identification and Evaluation of Adenium obesum Phytochemicals as Potential EGFR Inhibitors for Targeted Therapy Against Non-Small-Cell Lung Cancer**

Dear Editor and Reviewers,

We sincerely thank the Academic Editor and all reviewers for their valuable time and insightful comments. We have revised our manuscript thoroughly and addressed all comments point by point below. All changes have been incorporated into the revised version, with tracked changes indicated. We believe these revisions have significantly improved the quality and clarity of our manuscript. All the revisions are highlighted by yellow color.

**Response to the Editor**

We sincerely thank the Editor for the careful evaluation of our manuscript and for providing constructive guidance regarding the major issues that required attention. We fully acknowledge the concerns raised and have substantially revised the manuscript to improve the scientific rigor, balance, methodological transparency, and clinical relevance of the study.

In response to the Editor's comments, we have made the following major revisions:

1. **Experimental validation and interpretation of computational findings:** We have explicitly acknowledged throughout the revised manuscript that the present study is entirely computational and that the findings are hypothesis-generating. Claims suggesting confirmed EGFR inhibition or therapeutic efficacy have been moderated accordingly. Cardenolide is now described as a *computationally prioritized candidate* or *potential EGFR-targeting compound*, and the need for subsequent biochemical, cellular, and in vivo experimental validation has been clearly stated.
2. **Docking-score interpretation:** We have revised the molecular docking Results and Discussion to clarify that docking scores represent predicted binding characteristics and cannot independently establish binding affinity, inhibitory potency, or therapeutic efficacy. Comparisons with approved EGFR inhibitors have been reframed as computational observations rather than evidence of superior inhibitory activity.
3. **Clinically relevant EGFR mutations:** To improve the translational relevance of the study, we performed additional comparative molecular docking of all 16 investigated *Adenium obesum* phytochemicals against EGFR structures representing wild-type EGFR (PDB ID: 1M17), L858R/T790M EGFR (PDB ID: 4I22), and L858R/T790M/C797S EGFR (PDB ID: 6LUD). The revised manuscript now presents and discusses the compound-dependent differences in predicted docking behavior across these structural backgrounds. We have also clearly acknowledged that these analyses do not establish mutant EGFR inhibition or the ability to overcome clinically established EGFR resistance mechanisms.
4. **Rationale for Cardenolide prioritization:** We have clarified that Cardenolide was not selected solely on the basis of its docking score. Although SCHEMBL5933906 and Echujin demonstrated comparable or more favorable predicted docking scores, Cardenolide was computationally prioritized based on the overall balance of its predicted docking characteristics, physicochemical properties, drug-likeness, pharmacokinetic profile, and toxicity-related predictions. We have also explicitly acknowledged that SCHEMBL5933906 and Echujin were not subjected to molecular dynamics simulations and therefore that MD results were not used as a comparative criterion for selecting Cardenolide over these compounds.
5. **Pharmacokinetic limitations and Lipinski violations:** The revised manuscript now provides a more balanced discussion of the predicted drug-likeness and pharmacokinetic limitations of the investigated phytochemicals. In particular, we discuss the multiple predicted Lipinski rule violations, high molecular weight, elevated TPSA, and low predicted gastrointestinal absorption observed for several highly ranked glycosylated phytochemicals. We have also clarified that Cardenolide itself should not be considered uniformly optimal, particularly in view of its predicted lipophilicity, and that all ADME and toxicity predictions require experimental validation.
6. **Limitations of the computational approach:** The Discussion and Conclusion have been revised to provide a more balanced interpretation of the findings. The limitations of molecular docking, molecular dynamics, ADME prediction, toxicity prediction, and other computational approaches have been more explicitly acknowledged, and the need for experimental validation has been emphasized.
7. **Language and manuscript organization:** The manuscript has undergone extensive language editing to correct grammatical and typographical errors. The Introduction has been condensed to remove repetitive and unnecessary details while retaining the essential background, research gap, rationale, and objectives.
8. **Methodological transparency:** The Methods section has been revised to provide more consistent information regarding software versions, access dates, docking parameters, receptor structures, and grid-box settings. The grid-box parameters for the wild-type and mutation-containing EGFR structures are now reported in a consolidated table to facilitate reproducibility.
9. **Molecular interaction visualization:** The molecular docking figures have been improved by providing higher-resolution interaction images and informative two-dimensional protein–ligand interaction diagrams to facilitate clearer interpretation of the predicted binding interactions.
10. **Comparison with previously reported natural EGFR-related compounds:** The Discussion has been expanded to briefly contextualize the present findings with previously investigated natural products, including curcumin, epigallocatechin-3-gallate (EGCG), quercetin, resveratrol, berberine, and genistein. This comparison helps establish the novelty of investigating *Adenium obesum* phytochemicals through an integrated computational framework while maintaining appropriate caution regarding the distinction between EGFR-related activity and experimentally confirmed direct EGFR inhibition.

We believe that these comprehensive revisions have substantially strengthened the manuscript and have addressed the Editor's and Reviewer's concerns regarding the interpretation, methodological rigor, translational relevance, and presentation of the study. We have also carefully reviewed the revised manuscript to ensure that the conclusions are appropriately aligned with the computational nature of the evidence and do not exceed the limitations of the study.

We sincerely appreciate the Editor's and Reviewer's constructive comments, which have helped us improve the quality, clarity, and scientific rigor of the manuscript.

**Response to the Reviewers**

**Reviewer #1**

**Major Comments**

**Comment 1:** The study is entirely computational, and the conclusions regarding Cardenolide as a promising EGFR inhibitor are not supported by in vitro or in vivo validation.

**Author Response:**

We sincerely thank the reviewer for this important and constructive comment. We fully agree that the absence of experimental validation is an important limitation of the present study and that the computational findings should not be interpreted as evidence of experimentally confirmed EGFR inhibitory activity or therapeutic efficacy. The objective of our study was to computationally prioritize phytochemicals from *Adenium obesum* based on their predicted EGFR-binding characteristics, molecular interactions, structural stability, physicochemical properties, ADMET profiles, and toxicity predictions. We acknowledge that these computational approaches are hypothesis-generating and cannot substitute for direct biochemical, cellular, or in vivo validation.

In response to the reviewer's concern, we have carefully revised the manuscript to ensure that the conclusions are appropriately aligned with the evidence generated by the computational analyses. In particular, we have removed or modified statements that could be interpreted as claiming that Cardenolide is a confirmed EGFR inhibitor or that it possesses demonstrated therapeutic efficacy against NSCLC. Throughout the revised manuscript, we now consistently refer to Cardenolide as a **"computationally prioritized candidate," "putative EGFR-binding candidate,"** or **"potential EGFR-targeting compound,"** where appropriate.

We have also explicitly acknowledged that the present study is entirely computational and that the findings should be considered hypothesis-generating. We now clearly state that experimental validation is required to determine whether the predicted interactions translate into actual EGFR inhibitory activity and anticancer efficacy. Specifically, we have highlighted the need for biochemical assays, including direct EGFR kinase inhibition studies, followed by cellular validation in relevant NSCLC models. Further experimental studies will also be necessary to establish the pharmacokinetic properties, mechanism of action, and safety profile of Cardenolide.

The following major revisions have been made:

1. **Abstract:** The statement identifying Cardenolide as a "potential EGFR inhibitor" has been revised to describe it as a **"computationally prioritized candidate with favorable predicted EGFR-binding characteristics, structural stability, and physicochemical and toxicity profiles."** We have also explicitly stated that the study is entirely computational, that the findings are hypothesis-generating, and that biochemical and cellular experimental validation is required.
2. **Introduction:** We have revised the study rationale and objectives to clarify that the computational workflow is designed to **prioritize candidate EGFR-targeting compounds** rather than establish experimentally confirmed inhibitory activity. We now explicitly state that the computational findings provide a basis for selecting candidates for subsequent biochemical and cellular validation.
3. **Conclusion:** The conclusion has been substantially revised to clarify that the predicted docking scores, molecular interactions, molecular dynamics stability, and in silico ADMET and toxicity profiles do not establish experimentally confirmed EGFR inhibitory activity, anticancer efficacy, or therapeutic safety. Cardenolide is now presented as a **computationally prioritized candidate for further experimental investigation**, rather than as a confirmed EGFR inhibitor.
4. **Throughout the manuscript:** We have systematically reviewed the terminology and revised overstatements where necessary. Claims implying confirmed EGFR inhibition, therapeutic efficacy, or safety have been replaced with appropriately cautious terminology such as **"predicted EGFR-binding potential," "computationally prioritized candidate,"** and **"candidate EGFR-targeting compound."** Similarly, statements concerning ATP-site binding are now framed as predicted structural compatibility with the EGFR ATP-binding pocket rather than experimentally established ATP-competitive inhibition.

We believe these revisions have substantially improved the scientific rigor and transparency of the manuscript by ensuring that our conclusions accurately reflect the scope and limitations of the computational evidence. We are grateful to the reviewer for highlighting this important issue, which has helped us to better distinguish between computational prioritization and experimentally validated biological activity and to present Cardenolide as a testable candidate for future biochemical and cellular investigation rather than as a confirmed EGFR inhibitor.

**Comment 2:** The manuscript overstates the superiority of several phytochemicals over approved EGFR inhibitors based solely on docking scores, whereas docking energies alone are insufficient to establish inhibitory potency or therapeutic efficacy.

**Author Response:**

We sincerely thank the reviewer for this important observation. We fully agree that docking scores are model-dependent computational estimates and should not be interpreted as experimentally measured binding affinities, binding free energies, inhibitory potencies, or therapeutic efficacy. We acknowledge that our original wording could have inadvertently overstated the significance of the more favorable docking scores obtained by several *Adenium obesum* phytochemicals relative to the reference EGFR-targeting compounds.

In response to this comment, we have substantially revised the presentation and interpretation of the molecular docking results throughout the manuscript. We no longer describe the phytochemicals as having experimentally "higher affinity" or as "outperforming" approved EGFR-targeting drugs based solely on docking scores. Instead, the docking results are now presented as relative computational ranking metrics within the applied docking protocol, with more negative scores indicating more favorable predicted docking scores under the AutoDock Vina scoring function.

Specifically, the following changes have been made:

1. The molecular docking Results section has been revised to clarify that docking scores represent **model-dependent computational estimates** and cannot be directly interpreted as experimentally measured binding affinities, binding free energies, or inhibitory potencies.
2. The previous terminology of "high-affinity," "moderate-affinity," and "lower-affinity" binders has been replaced with more appropriate descriptions such as **"top-ranked compounds based on docking score," "intermediate-ranked compounds,"** and **"compounds with relatively less favorable docking scores."** This change was made to avoid implying that the selected docking-score ranges represent experimentally validated affinity thresholds.
3. Statements suggesting that phytochemicals "outperformed" or possessed "stronger binding" than approved EGFR inhibitors have been revised. The manuscript now states that certain phytochemicals received **more favorable docking scores than the reference compounds under the same computational docking protocol**, while explicitly emphasizing that this numerical difference does not demonstrate superior experimental binding affinity, inhibitory potency, or therapeutic efficacy.
4. The comparison with Osimertinib, Erlotinib, Afatinib, and Lazertinib has been retained as a **computational reference comparison** rather than as evidence of pharmacological superiority. We now explicitly state that similar or more favorable docking scores indicate only relative ranking within the applied computational framework and do not establish equivalent or superior biological activity.
5. The interpretation of compounds with intermediate and relatively less favorable docking scores has also been revised. We no longer infer pharmacological relevance solely from their docking-score ranges. Instead, we emphasize that docking results should be considered together with binding-mode plausibility, protein–ligand interaction patterns, physicochemical properties, ADMET characteristics, toxicity predictions, and molecular dynamics behavior.
6. A clarification has been added to the docking results and Table 3 indicating that docking scores should be interpreted as **relative computational prioritization metrics** and not as direct measures of experimental binding affinity or inhibitory potency.
7. The Discussion has been revised to explicitly acknowledge that differences in docking scores cannot establish the superiority of the phytochemicals over clinically used EGFR-targeting drugs. We now emphasize that experimental biochemical assays, including direct EGFR kinase inhibition measurements, are required to determine actual binding affinity and inhibitory potency.

These revisions have substantially moderated the interpretation of the docking results and aligned our conclusions with the limitations of computational docking. We now use the docking analysis primarily as an **initial prioritization step** within a broader integrated computational workflow rather than as standalone evidence of EGFR inhibitory activity or therapeutic efficacy. We appreciate the reviewer for highlighting this issue, which has helped us improve the scientific rigor and precision of the manuscript.

**Comment 3**: The use of only the wild-type EGFR structure (PDB: 1M17) does not adequately represent clinically important resistant NSCLC mutations such as L858R, T790M, and C797S, reducing the clinical relevance of the study.

**Authors Response:**

We sincerely thank the reviewer for raising this important concern. We agree that an analysis restricted to wild-type EGFR would provide limited insight into the potential binding behavior of candidate compounds in clinically relevant mutant EGFR backgrounds. We have therefore expanded the molecular docking analysis to include additional EGFR structures carrying clinically important mutation combinations associated with NSCLC biology and therapeutic resistance.

Specifically, in addition to the wild-type EGFR structure (PDB ID: 1M17), all 16 *Adenium obesum* phytochemicals investigated in the present study were docked against two additional experimentally determined EGFR mutant structures: PDB ID 4I22, representing an EGFR background carrying L858R and T790M mutations, and PDB ID 6LUD, representing an EGFR background carrying L858R, T790M, and C797S mutations. Thus, the revised analysis now compares predicted docking behavior across wild-type EGFR, an L858R/T790M double-mutant background, and an L858R/T790M/C797S triple-mutant background.

The comparative analysis revealed compound-dependent changes in predicted docking scores across the three EGFR structural backgrounds. Several phytochemicals, including SCHEMBL5933906, Obebioside A, Strospeside, Obeside C, Obetrioside B, Obebioside B, Hongheloside C, and Obebioside C, exhibited similar or more favorable docking scores in at least one mutant structural background compared with wild-type EGFR. In contrast, other compounds, including Cardenolide and Echujin, showed less favorable predicted docking scores in the mutant systems. These results indicate that the effects of the mutation-associated structural changes on predicted ligand docking behavior are compound-dependent.

We have carefully avoided interpreting these findings as evidence of experimentally demonstrated activity against mutant EGFR or as proof that any candidate can overcome EGFR TKI resistance. In particular, because PDB 4I22 and PDB 6LUD contain combinations of mutations rather than isolated single mutations, we have explicitly described the analysis as a comparative evaluation of wild-type, double-mutant, and triple-mutant EGFR structural backgrounds. We do not attribute observed docking-score changes to the isolated effect of any individual mutation.

The corresponding methodology has been added to the revised Methods section, and the comparative docking results for all 16 phytochemicals have been incorporated into the revised Results section and presented in Table 4. We have also added an explicit discussion of the limitations of computational mutant docking and clarified that experimental biochemical and cellular validation is required to determine whether the predicted interactions translate into actual inhibitory activity against mutant EGFR.

We believe that this additional analysis substantially strengthens the clinical relevance of the manuscript by extending the computational evaluation beyond wild-type EGFR and examining the predicted behavior of all 16 phytochemicals in EGFR structural backgrounds containing clinically relevant mutation combinations. We are grateful to the reviewer for this valuable suggestion, which has enabled us to provide a more comprehensive and clinically contextualized computational assessment.

**Comment 4**: The selection of Cardenolide as the lead compound is not sufficiently justified because SCHEMBL5933906 and other phytochemicals exhibited equal or better docking scores.

**Authors' Response:**

We sincerely thank the Reviewer for this important and constructive observation. We agree that the previous version of the manuscript did not sufficiently explain the rationale for selecting Cardenolide for subsequent detailed computational investigation, particularly given that SCHEMBL5933906 and Echujin exhibited comparable or more favorable predicted docking scores. We have therefore revised the manuscript to clarify that the selection of Cardenolide was not based solely on its molecular docking score.

In the revised manuscript, we explicitly acknowledge that SCHEMBL5933906 exhibited a slightly more favorable predicted docking score against wild-type EGFR (−10.0 kcal/mol) than Cardenolide (−9.9 kcal/mol) and also showed favorable predicted docking scores against the L858R/T790M and L858R/T790M/C797S EGFR structures. Echujin likewise demonstrated highly favorable predicted docking characteristics. We have therefore avoided presenting Cardenolide as the compound with the strongest docking performance or as universally superior to the other investigated phytochemicals.

Instead, we have clarified that Cardenolide was computationally prioritized based on the overall balance of the evaluated computational parameters, including predicted docking characteristics, physicochemical properties, drug-likeness, pharmacokinetic properties, and toxicity-related predictions. In particular, Cardenolide exhibited a comparatively favorable physicochemical and pharmacokinetic profile, with a molecular weight of 342.00 g/mol, one predicted Lipinski rule violation, a TPSA of 26.30 Å², a predicted bioavailability score of 0.55, and high predicted gastrointestinal absorption. In contrast, SCHEMBL5933906 and Echujin exhibited substantially higher molecular weights (764.94 and 843.00 g/mol, respectively), multiple predicted Lipinski rule violations, higher TPSA values, lower predicted bioavailability scores (0.17 for both compounds), and low predicted gastrointestinal absorption. These differences were considered relevant when prioritizing compounds for further computational characterization.

We have also clarified an important methodological point regarding molecular dynamics simulation. Cardenolide was selected for molecular dynamics simulation after the initial integrated assessment of docking and predicted physicochemical, pharmacokinetic, and toxicity-related properties. Molecular dynamics simulation was subsequently performed to further investigate the temporal stability and interaction behavior of the selected Cardenolide–EGFR complex. Because SCHEMBL5933906 and Echujin were not subjected to molecular dynamics simulations in the present study, we have removed any implication that Cardenolide was selected because it demonstrated superior molecular dynamics stability relative to these compounds. We now explicitly state that the molecular dynamics results provide additional characterization of the selected Cardenolide–EGFR complex but do not establish comparative dynamic superiority over compounds that were not simulated.

Furthermore, we have moderated our interpretation throughout the manuscript and now describe Cardenolide as a "computationally prioritized candidate" rather than a definitively superior lead or experimentally confirmed EGFR inhibitor. We also explicitly acknowledge that the computational findings are hypothesis-generating and that experimental biochemical and cellular studies are required to determine whether the predicted characteristics of Cardenolide translate into measurable EGFR-targeting activity.

These revisions have been incorporated into the Results and Discussion sections, particularly in the newly revised discussion of the rationale for computational prioritization of Cardenolide. The relevant sections now explicitly acknowledge the favorable docking performance of SCHEMBL5933906 and Echujin while explaining why Cardenolide was prioritized for further computational investigation based on its comparatively balanced predicted developability profile.

We appreciate the Reviewer for raising this issue, as it has helped us improve the transparency and scientific rigor of the candidate-selection rationale and avoid overinterpretation of docking results.

**Comment 5:** Several top-ranked phytochemicals show multiple Lipinski rule violations and poor predicted gastrointestinal absorption.

**Author Response:**

We sincerely thank the Reviewer for this important observation. We agree that several of the top-ranked *Adenium obesum* phytochemicals exhibited multiple predicted Lipinski rule violations and low predicted gastrointestinal (GI) absorption, and that these characteristics may represent important developability limitations that should be considered when interpreting their potential as drug candidates. We have therefore revised the manuscript to provide a more balanced and transparent interpretation of the drug-likeness and pharmacokinetic profiles of all 16 investigated phytochemicals.

Specifically, we have expanded and revised the "Analysis of Drug-Like Properties" section in the Results to explicitly report the differences in molecular weight, hydrogen-bonding capacity, topological polar surface area (TPSA), Lipinski rule compliance, predicted bioavailability, GI absorption, and lipophilicity across the complete phytochemical panel (Table 5). The revised analysis now clearly acknowledges that several compounds with favorable predicted docking characteristics including SCHEMBL5933906, Obebioside A, Obetrioside B, Obebioside B, Echujin, Hongheloside C, Obebioside C, Obetrioside A, and Mono-O-acetylacoschimperoside P also exhibited two or three predicted Lipinski rule violations and low predicted GI absorption. We have clarified that these physicochemical and pharmacokinetic characteristics may present challenges for oral drug development and systemic exposure.

We have also revised the interpretation to avoid equating favorable target-binding predictions with overall drug-likeness or therapeutic suitability. The revised manuscript now emphasizes that molecular docking, drug-likeness, and pharmacokinetic properties represent complementary but distinct aspects of computational candidate evaluation. Thus, a favorable predicted docking score alone does not necessarily indicate favorable oral absorption or overall developability.

In addition, we have clarified that Lipinski's Rule of Five is an empirical guideline rather than an absolute exclusion criterion, particularly in the context of natural products. Therefore, predicted Lipinski violations do not necessarily preclude biological activity. However, multiple violations combined with high molecular weight, elevated polarity, and low predicted GI absorption may indicate potential developability challenges and may warrant structural optimization, formulation strategies, alternative delivery approaches, or experimental pharmacokinetic evaluation.

We have also acknowledged the potential limitations of Cardenolide's own predicted pharmacokinetic profile. Although Cardenolide exhibited comparatively favorable characteristics in terms of molecular weight, number of Lipinski violations, TPSA, predicted bioavailability score, and GI absorption relative to several highly glycosylated compounds, it also showed a relatively high predicted consensus Log Po/w value (5.22) and low predicted water solubility. These properties are now explicitly discussed as potential limitations rather than being overlooked. Accordingly, the manuscript no longer presents Cardenolide as having universally favorable ADME properties.

Importantly, the revised Results section presents the drug-likeness characteristics of all 16 phytochemicals objectively without prematurely designating a lead compound. The rationale for the subsequent computational prioritization of Cardenolide is discussed separately in the Discussion based on the integrated evidence from docking, predicted interaction characteristics, molecular dynamics, drug-likeness, ADME, toxicity, and other computational analyses. This distinction has been introduced to ensure that the selection of Cardenolide is not based solely on docking performance or a single pharmacokinetic parameter.

Finally, we have explicitly clarified throughout the revised manuscript that all drug-likeness, ADME, and pharmacokinetic findings are computational predictions and should not be interpreted as experimental evidence of oral bioavailability, systemic exposure, or therapeutic efficacy. Experimental pharmacokinetic and biochemical studies will be required to validate these predictions and determine the actual developability and therapeutic potential of the identified compounds.

**Minor Comments**

**Comment 1:** The manuscript contains several grammatical errors and typographical mistakes that require careful English language editing throughout the text.

**Author Response:**

Thank you for this valuable comment. The manuscript has been thoroughly reviewed and carefully edited throughout to correct grammatical errors, typographical mistakes, sentence structure, and language inconsistencies. The revised manuscript has also been carefully proofread to improve overall clarity, readability, and academic English.

**Comment 2:**The Introduction is excessively detailed and repetitive in several sections, and could be condensed.

**Author Response:**

Thank you for this helpful suggestion. The Introduction has been carefully revised and condensed by removing repetitive statements and unnecessary details while retaining the essential background, research gap, rationale, and objectives of the study. These revisions have improved the focus and overall readability of the Introduction.

**Comment 3:** Software versions, access dates, and computational parameters should be reported consistently for all bioinformatics tools to improve methodological transparency.

**Author Response:**

We appreciate this important suggestion. The Methods section has been carefully reviewed and revised to provide software names, version numbers, access dates where applicable, and relevant computational parameters consistently throughout the manuscript. Additional details regarding the molecular docking protocols, EGFR structural models, grid-box parameters, and computational settings have also been provided to improve methodological transparency and reproducibility.

**Comment 4:** The docking interaction figures would benefit from higher resolution. A 2D interaction diagram can prove to be useful to understand the active site of the proteins.

**Author Response:**

Thank you for this constructive suggestion. The molecular docking interaction figures have been revised and replaced with higher-resolution images. In addition, two-dimensional protein–ligand interaction diagrams have been included to provide a clearer visualization of the predicted binding interactions between the selected phytochemical and the EGFR binding site.

**Comment 5:** The discussion should include a more comprehensive comparison of the identified phytochemicals with previously reported natural EGFR inhibitors to better establish the novelty of the findings.

**Author Response:**

We thank the reviewer for this helpful suggestion. The Discussion section has been revised to include a concise comparison of the investigated *Adenium obesum* phytochemicals with previously reported natural products, including curcumin, epigallocatechin-3-gallate (EGCG), quercetin, resveratrol, berberine, and genistein, which have been investigated in relation to EGFR activity or EGFR-associated signaling. This comparison places the present findings within the broader context of natural-product-based EGFR research and clarifies the novelty of systematically evaluating *A. obesum* phytochemicals using an integrated computational workflow. The revised text also emphasizes that the present findings are computational and hypothesis-generating, requiring experimental validation.

**Reviewer #2**

**Comment:**

Limited Novelty: The study follows a well-established experimental strategy without introducing a new concept, methodology, or significant mechanistic insight. Similar studies have already been reported extensively in the literature.

Limited Depth of Investigation.

Scientific Impact: While the work may have some confirmatory value, its potential impact is limited because it does not provide substantial conceptual, methodological, or transnational advancement.

**Author Response:**

We sincerely thank the reviewer for the thoughtful assessment of the novelty, depth, and potential scientific impact of our study. We agree that the computational approaches employed in this work, including molecular docking, molecular dynamics simulation, ADME and toxicity prediction, DFT analysis, and cancer bioinformatics, are established methods in computational drug discovery. We have therefore revised the manuscript to avoid implying that the methodological framework itself represents a fundamentally new computational strategy.

However, we respectfully clarify that the novelty of the present study lies primarily in the systematic application and integration of these complementary computational approaches to investigate the largely underexplored phytochemical repertoire of *Adenium obesum* in the context of EGFR-targeted NSCLC research. To the best of our knowledge, a comprehensive computational assessment integrating these approaches for *A. obesum*-derived phytochemicals against EGFR, together with comparative evaluation across wild-type and clinically relevant mutation-containing EGFR structures, has not previously been reported.

In response to the reviewer's concern regarding the depth of investigation, we have substantially strengthened the revised manuscript by expanding the computational analysis beyond the initial docking-based screening. Specifically, we have incorporated comparative docking against EGFR structures representing L858R/T790M and L858R/T790M/C797S mutation backgrounds, comprehensive physicochemical and pharmacokinetic assessment, toxicity prediction, molecular dynamics simulation of the computationally prioritized candidate, DFT-based electronic characterization, evolutionary conservation analysis, and cancer bioinformatics analyses. These additions provide a broader characterization of the computationally prioritized candidate and place the findings within the biological and clinical context of EGFR-driven NSCLC.

We have also revised the Discussion to provide a more balanced interpretation of the study's contribution. In particular, we now explicitly acknowledge that the present work does not establish experimentally confirmed EGFR inhibition, therapeutic efficacy, or the ability to overcome EGFR TKI resistance. Rather, the study is intended to generate testable hypotheses and computationally prioritize candidates for subsequent biochemical, cellular, and in vivo investigation.

Furthermore, we have moderated the claims regarding the significance and translational implications of the findings throughout the manuscript. The computationally prioritized compound is now described as a "computationally prioritized candidate" or "potential EGFR-targeting compound", rather than as a confirmed EGFR inhibitor. The revised manuscript also emphasizes that experimental validation will be essential to determine whether the predicted computational characteristics translate into measurable biological activity.

We appreciate the reviewer’s comment, which has helped us clarify the actual contribution of the study and avoid overstating its novelty or scientific impact. We believe that the revised manuscript now presents a more appropriately balanced contribution: rather than proposing a new computational methodology, it provides an integrated, hypothesis-generating computational investigation of an underexplored natural-product source and identifies candidates that may warrant future experimental evaluation.
