# Supplementary material for: Computational identification and evaluation of Adenium obesum phytochemicals as potential EGFR inhibitors for targeted therapy against non-small-cell lung cancer
Source: PLoS One. 2026 Aug 31;21(8):e0357096. doi: 10.1371/journal.pone.0357096 (PMC13529004; doi:10.1371/journal.pone.0357096)
Supplement: Peer review history

# Peer review: Decision Letter 0

**DOI:** 10.1371/journal.pone.0357096.r001

**Authors:** Lalit Samant

15 Jul 2026

Computational Identification and Evaluation of Adenium obesum Phytochemicals as Potential EGFR Inhibitors for Targeted Therapy Against Non-Small-Cell Lung Cancer

PLOS One

Dear Dr. Kumer,

Thank you for submitting your manuscript to PLOS ONE. After careful consideration, we feel that it has merit but does not fully meet PLOS ONE’s publication criteria as it currently stands. Therefore, we invite you to submit a revised version of the manuscript that addresses the points raised during the review process.

Please submit your revised manuscript by Sep 13 2026 11:59PM. If you will need more time than this to complete your revisions, please reply to this message or contact the journal office at plosone@plos.org. When you're ready to submit your revision, log on to https://www.editorialmanager.com/pone/ and select the 'Submissions Needing Revision' folder to locate your manuscript file.

Please include the following items when submitting your revised manuscript:

- A letter that responds to each point raised by the academic editor and reviewer(s). You should upload this letter as a separate file labeled 'Response to Reviewers'.
- A marked-up copy of your manuscript that highlights changes made to the original version. You should upload this as a separate file labeled 'Revised Manuscript with Track Changes'.
- An unmarked version of your revised paper without tracked changes. You should upload this as a separate file labeled 'Manuscript'.

If you would like to make changes to your financial disclosure, please include your updated statement in your cover letter. Guidelines for resubmitting your figure files are available below the reviewer comments at the end of this letter.

If applicable, we recommend that you deposit your laboratory protocols in protocols.io to enhance the reproducibility of your results. Protocols.io assigns your protocol its own identifier (DOI) so that it can be cited independently in the future. For instructions see: https://journals.plos.org/plosone/s/submission-guidelines#loc-laboratory-protocols. Additionally, PLOS ONE offers an option for publishing peer-reviewed Lab Protocol articles, which describe protocols hosted on protocols.io. Read more information on sharing protocols at https://plos.org/protocols?utm_medium=editorial-email&utm_source=authorletters&utm_campaign=protocols.

As the corresponding author, your ORCID iD is verified in the submission system and will appear in the published article. PLOS supports the use of ORCID, and we encourage all coauthors to register for an ORCID iD and use it as well. Please encourage your coauthors to verify their ORCID iD within the submission system before final acceptance, as unverified ORCID iDs will not appear in the published article. *Only*  the individual author can complete the verification step; PLOS staff *cannot*  verify ORCID iDs on behalf of authors.

We look forward to receiving your revised manuscript.

Kind regards,

Lalit Samant

Academic Editor

PLOS One

Journal Requirements:

When submitting your revision, we need you to address these additional requirements.

1.Please ensure that your manuscript meets PLOS ONE's style requirements, including those for file naming. The PLOS ONE style templates can be found at

https://journals.plos.org/plosone/s/file?id=wjVg/PLOSOne_formatting_sample_main_body.pdf and

https://journals.plos.org/plosone/s/file?id=ba62/PLOSOne_formatting_sample_title_authors_affiliations.pdf

2. Please note that PLOS One has specific guidelines on code sharing for submissions in which author-generated code underpins the findings in the manuscript. In these cases, we expect all author-generated code to be made available without restrictions upon publication of the work. Please review our guidelines at https://journals.plos.org/plosone/s/materials-and-software-sharing#loc-sharing-code and ensure that your code is shared in a way that follows best practice and facilitates reproducibility and reuse.

3. When completing the data availability statement of the submission form, you indicated that you will make your data available on acceptance. We strongly recommend all authors decide on a data sharing plan before acceptance, as the process can be lengthy and hold up publication timelines. Please note that, though access restrictions are acceptable now, your entire data will need to be made freely accessible if your manuscript is accepted for publication. This policy applies to all data except where public deposition would breach compliance with the protocol approved by your research ethics board. If you are unable to adhere to our open data policy, please kindly revise your statement to explain your reasoning and we will seek the editor's input on an exemption. Please be assured that, once you have provided your new statement, the assessment of your exemption will not hold up the peer review process.

4. If the reviewer comments include a recommendation to cite specific previously published works, please review and evaluate these publications to determine whether they are relevant and should be cited. There is no requirement to cite these works unless the editor has indicated otherwise.

Additional Editor Comments:

Based on the reviewers' comments, the manuscript requires **major revision**  before it can be considered further for publication.

The principal concerns relate to the reliance on computational analyses without experimental validation, the overinterpretation of molecular docking results, and the limited clinical relevance arising from the exclusive use of the wild-type EGFR structure. In particular, the conclusions regarding the identification of Cardenolide as a promising EGFR inhibitor should be moderated and supported by stronger scientific justification. The rationale for selecting the lead compound should be clarified, especially in light of other compounds demonstrating comparable or superior docking scores. Additionally, the pharmacokinetic limitations of the proposed lead compounds, including Lipinski rule violations and poor predicted gastrointestinal absorption, should be critically discussed.

The authors should also strengthen the manuscript by providing a balanced interpretation of the computational findings, acknowledging the limitations of molecular docking as a predictive tool, and discussing the need for subsequent in vitro and in vivo validation. Inclusion of analyses involving clinically relevant EGFR resistance mutations (e.g., L858R, T790M, and C797S), or a clear justification for their omission, would substantially improve the translational relevance of the study.

In addition, the manuscript requires careful language editing, condensation of the Introduction to improve clarity, consistent reporting of software versions and computational parameters, higher-quality molecular interaction figures (including informative 2D interaction diagrams), and a more comprehensive comparison with previously reported natural EGFR inhibitors to better establish the novelty and significance of the findings.

Overall, the study presents potentially interesting computational observations; however, the current manuscript overstates its conclusions and requires substantial revision to ensure that the interpretations are fully supported by the presented evidence. I therefore recommend **Major Revision** .

[Note: HTML markup is below. Please do not edit.]

Reviewer's Responses to Questions

**Comments to the Author**

1. Is the manuscript technically sound, and do the data support the conclusions?

Reviewer #1: Yes

Reviewer #2: No

**********

2. Has the statistical analysis been performed appropriately and rigorously?

Reviewer #1: No

Reviewer #2: No

**********

3. Have the authors made all data underlying the findings in their manuscript fully available?

The PLOS Data policy

Reviewer #1: Yes

Reviewer #2: No

**********

4. Is the manuscript presented in an intelligible fashion and written in standard English?

Reviewer #1: Yes

Reviewer #2: Yes

**********

Reviewer #1: Major Comments

1. The study is entirely computational, and the conclusions regarding Cardenolide as a promising EGFR inhibitor are not supported by in vitro or in vivo validation.

2. The manuscript overstates the superiority of several phytochemicals over approved EGFR inhibitors based solely on docking scores, whereas docking energies alone are insufficient to establish inhibitory potency or therapeutic efficacy.

3. The use of only the wild-type EGFR structure (PDB: 1M17) does not adequately represent clinically important resistant NSCLC mutations such as L858R, T790M, and C797S, reducing the clinical relevance of the study.

4. The selection of Cardenolide as the lead compound is not sufficiently justified because SCHEMBL5933906 and other phytochemicals exhibited equal or better docking scores.

5. Several top-ranked phytochemicals show multiple Lipinski rule violations and poor predicted gastrointestinal absorption.

Minor Comments

1. The manuscript contains several grammatical errors and typographical mistakes that require careful English language editing throughout the text.

2. The Introduction is excessively detailed and repetitive in several sections, and could be condensed.

3. Software versions, access dates, and computational parameters should be reported consistently for all bioinformatics tools to improve methodological transparency.

4. The docking interaction figures would benefit from higher resolution. A 2D interaction diagram ca prove to be useful to understand the active site of the proteins.

5. The discussion should include a more comprehensive comparison of the identified phytochemicals with previously reported natural EGFR inhibitors to better establish the novelty of the findings.

Reviewer #2: Limited Novelty: The study follows a well-established experimental strategy without introducing a new concept, methodology, or significant mechanistic insight. Similar studies have already been reported extensively in the literature.

Limited Depth of Investigation.

Scientific Impact: While the work may have some confirmatory value, its potential impact is limited because it does not provide substantial conceptual, methodological, or transnational advancement.

**********

what does this mean?). If published, this will include your full peer review and any attached files

If you choose “no”, your identity will remain anonymous but your review may still be made public.

**Do you want your identity to be public for this peer review?**  For information about this choice, including consent withdrawal, please see our Privacy Policy

Reviewer #1: **Yes:**  Dr. Sanket Bapat

Reviewer #2: No

**********

[NOTE: If reviewer comments were submitted as an attachment file, they will be attached to this email and accessible via the submission site. Please log into your account, locate the manuscript record, and check for the action link "View Attachments". If this link does not appear, there are no attachment files.]

To ensure your figures meet our technical requirements, please review our figure guidelines: https://journals.plos.org/plosone/s/figures

You may also use PLOS’s free figure tool, NAAS, to help you prepare publication quality figures: https://journals.plos.org/plosone/s/figures#loc-tools-for-figure-preparation.

NAAS will assess whether your figures meet our technical requirements by comparing each figure against our figure specifications.

# Author comment: Author response to Decision Letter 1

**DOI:** 10.1371/journal.pone.0357096.r002

4 Aug 2026

Response to the Editor and Reviewers

Manuscript ID: PONE-D-26-27333

Title: Computational Identification and Evaluation of Adenium obesum Phytochemicals as Potential EGFR Inhibitors for Targeted Therapy Against Non-Small-Cell Lung Cancer

Dear Editor and Reviewers,

We sincerely thank the Academic Editor and all reviewers for their valuable time and insightful comments. We have revised our manuscript thoroughly and addressed all comments point by point below. All changes have been incorporated into the revised version, with tracked changes indicated. We believe these revisions have significantly improved the quality and clarity of our manuscript. All the revisions are highlighted by yellow color.

Response to the Editor

We sincerely thank the Editor for the careful evaluation of our manuscript and for providing constructive guidance regarding the major issues that required attention. We fully acknowledge the concerns raised and have substantially revised the manuscript to improve the scientific rigor, balance, methodological transparency, and clinical relevance of the study.

In response to the Editor's comments, we have made the following major revisions:

1. Experimental validation and interpretation of computational findings: We have explicitly acknowledged throughout the revised manuscript that the present study is entirely computational and that the findings are hypothesis-generating. Claims suggesting confirmed EGFR inhibition or therapeutic efficacy have been moderated accordingly. Cardenolide is now described as a computationally prioritized candidate or potential EGFR-targeting compound, and the need for subsequent biochemical, cellular, and in vivo experimental validation has been clearly stated.

2. Docking-score interpretation: We have revised the molecular docking Results and Discussion to clarify that docking scores represent predicted binding characteristics and cannot independently establish binding affinity, inhibitory potency, or therapeutic efficacy. Comparisons with approved EGFR inhibitors have been reframed as computational observations rather than evidence of superior inhibitory activity.

3. Clinically relevant EGFR mutations: To improve the translational relevance of the study, we performed additional comparative molecular docking of all 16 investigated Adenium obesum phytochemicals against EGFR structures representing wild-type EGFR (PDB ID: 1M17), L858R/T790M EGFR (PDB ID: 4I22), and L858R/T790M/C797S EGFR (PDB ID: 6LUD). The revised manuscript now presents and discusses the compound-dependent differences in predicted docking behavior across these structural backgrounds. We have also clearly acknowledged that these analyses do not establish mutant EGFR inhibition or the ability to overcome clinically established EGFR resistance mechanisms.

4. Rationale for Cardenolide prioritization: We have clarified that Cardenolide was not selected solely on the basis of its docking score. Although SCHEMBL5933906 and Echujin demonstrated comparable or more favorable predicted docking scores, Cardenolide was computationally prioritized based on the overall balance of its predicted docking characteristics, physicochemical properties, drug-likeness, pharmacokinetic profile, and toxicity-related predictions. We have also explicitly acknowledged that SCHEMBL5933906 and Echujin were not subjected to molecular dynamics simulations and therefore that MD results were not used as a comparative criterion for selecting Cardenolide over these compounds.

5. Pharmacokinetic limitations and Lipinski violations: The revised manuscript now provides a more balanced discussion of the predicted drug-likeness and pharmacokinetic limitations of the investigated phytochemicals. In particular, we discuss the multiple predicted Lipinski rule violations, high molecular weight, elevated TPSA, and low predicted gastrointestinal absorption observed for several highly ranked glycosylated phytochemicals. We have also clarified that Cardenolide itself should not be considered uniformly optimal, particularly in view of its predicted lipophilicity, and that all ADME and toxicity predictions require experimental validation.

6. Limitations of the computational approach: The Discussion and Conclusion have been revised to provide a more balanced interpretation of the findings. The limitations of molecular docking, molecular dynamics, ADME prediction, toxicity prediction, and other computational approaches have been more explicitly acknowledged, and the need for experimental validation has been emphasized.

7. Language and manuscript organization: The manuscript has undergone extensive language editing to correct grammatical and typographical errors. The Introduction has been condensed to remove repetitive and unnecessary details while retaining the essential background, research gap, rationale, and objectives.

8. Methodological transparency: The Methods section has been revised to provide more consistent information regarding software versions, access dates, docking parameters, receptor structures, and grid-box settings. The grid-box parameters for the wild-type and mutation-containing EGFR structures are now reported in a consolidated table to facilitate reproducibility.

9. Molecular interaction visualization: The molecular docking figures have been improved by providing higher-resolution interaction images and informative two-dimensional protein–ligand interaction diagrams to facilitate clearer interpretation of the predicted binding interactions.

10. Comparison with previously reported natural EGFR-related compounds: The Discussion has been expanded to briefly contextualize the present findings with previously investigated natural products, including curcumin, epigallocatechin-3-gallate (EGCG), quercetin, resveratrol, berberine, and genistein. This comparison helps establish the novelty of investigating Adenium obesum phytochemicals through an integrated computational framework while maintaining appropriate caution regarding the distinction between EGFR-related activity and experimentally confirmed direct EGFR inhibition.

We believe that these comprehensive revisions have substantially strengthened the manuscript and have addressed the Editor's and Reviewer's concerns regarding the interpretation, methodological rigor, translational relevance, and presentation of the study. We have also carefully reviewed the revised manuscript to ensure that the conclusions are appropriately aligned with the computational nature of the evidence and do not exceed the limitations of the study.

We sincerely appreciate the Editor's and Reviewer's constructive comments, which have helped us improve the quality, clarity, and scientific rigor of the manuscript.

Response to the Reviewers

Reviewer #1

Major Comments

Comment 1: The study is entirely computational, and the conclusions regarding Cardenolide as a promising EGFR inhibitor are not supported by in vitro or in vivo validation.

Author Response:

We sincerely thank the reviewer for this important and constructive comment. We fully agree that the absence of experimental validation is an important limitation of the present study and that the computational findings should not be interpreted as evidence of experimentally confirmed EGFR inhibitory activity or therapeutic efficacy. The objective of our study was to computationally prioritize phytochemicals from Adenium obesum based on their predicted EGFR-binding characteristics, molecular interactions, structural stability, physicochemical properties, ADMET profiles, and toxicity predictions. We acknowledge that these computational approaches are hypothesis-generating and cannot substitute for direct biochemical, cellular, or in vivo validation.

In response to the reviewer's concern, we have carefully revised the manuscript to ensure that the conclusions are appropriately aligned with the evidence generated by the computational analyses. In particular, we have removed or modified statements that could be interpreted as claiming that Cardenolide is a confirmed EGFR inhibitor or that it possesses demonstrated therapeutic efficacy against NSCLC. Throughout the revised manuscript, we now consistently refer to Cardenolide as a "computationally prioritized candidate," "putative EGFR-binding candidate," or "potential EGFR-targeting compound," where appropriate.

We have also explicitly acknowledged that the present study is entirely computational and that the findings should be considered hypothesis-generating. We now clearly state that experimental validation is required to determine whether the predicted interactions translate into actual EGFR inhibitory activity and anticancer efficacy. Specifically, we have highlighted the need for biochemical assays, including direct EGFR kinase inhibition studies, followed by cellular validation in relevant NSCLC models. Further experimental studies will also be necessary to establish the pharmacokinetic properties, mechanism of action, and safety profile of Cardenolide.

The following major revisions have been made:

1. Abstract: The statement identifying Cardenolide as a "potential EGFR inhibitor" has been revised to describe it as a "computationally prioritized candidate with favorable predicted EGFR-binding characteristics, structural stability, and physicochemical and toxicity profiles." We have also explicitly stated that the study is entirely computational, that the findings are hypothesis-generating, and that biochemical and cellular experimental validation is required.

2. Introduction: We have revised the study rationale and objectives to clarify that the computational workflow is designed to prioritize candidate EGFR-targeting compounds rather than establish experimentally confirmed inhibitory activity. We now explicitly state that the computational findings provide a basis for selecting candidates for subsequent biochemical and cellular validation.

3. Conclusion: The conclusion has been substantially revised to clarify that the predicted docking scores, molecular interactions, molecular dynamics stability, and in silico ADMET and toxicity profiles do not establish experimentally confirmed EGFR inhibitory activity, anticancer efficacy, or therapeutic safety. Cardenolide is now presented as a computationally prioritized candidate for further experimental investigation, rather than as a confirmed EGFR inhibitor.

4. Throughout the manuscript: We have systematically reviewed the terminology and revised overstatements where necessary. Claims implying confirmed EGFR inhibition, therapeutic efficacy, or safety have been replaced with appropriately cautious terminology such as "predicted EGFR-binding potential," "computationally prioritized candidate," and "candidate EGFR-targeting compound." Similarly, statements concerning ATP-site binding are now framed as predicted structural compatibility with the EGFR ATP-binding pocket rather than experimentally established ATP-competitive inhibition.

We believe these revisions have substantially improved the scientific rigor and transparency of the manuscript by ensuring that our conclusions accurately reflect the scope and limitations of the computational evidence. We are grateful to the reviewer for highlighting this important issue, which has helped us to better distinguish between computational prioritization and experimentally validated biological activity and to present Cardenolide as a testable candidate for future biochemical and cellular investigation rather than as a confirmed EGFR inhibitor.

Comment 2: The manuscript overstates the superiority of several phytochemicals over approved EGFR inhibitors based solely on docking scores, whereas docking energies alone are insufficient to establish inhibitory potency or therapeutic efficacy.

Author Response:

We sincerely thank the reviewer for this important observation. We fully agree that docking scores are model-dependent computational estimates and should not be interpreted as experimentally measured binding affinities, binding free energies, inhibitory potencies, or therapeutic efficacy. We acknowledge that our original wording could have inadvertently overstated the significance of the more favorable docking scores obtained by several Adenium obesum phytochemicals relative to the reference EGFR-targeting compounds.

In response to this comment, we have substantially revised the presentation and interpretation of the molecular docking results throughout the manuscript. We no longer describe the phytochemicals as having experimentally "higher affinity" or as "outperforming" approved EGFR-targeting drugs based solely on docking scores. Instead, the docking results are now presented as relative computational ranking metrics within the applied docking protocol, with more negative scores indicating more favorable predicted docking scores under the AutoDock Vina scoring function.

Specifically, the following changes have been made:

1. The molecular docking Results section has been revised to clarify that docking scores represent model-dependent computational estimates and cannot be directly interpreted as experimentally measured binding affinities, binding free energies, or inhibitory potencies.

2. The previous terminology of "high-affinity," "moderate-affinity," and "lower-affinity" binders has been replaced with more appropriate descriptions such as "top-ranked compounds based on docking score," "intermediate-ranked compounds," and "compounds with relatively less favorable docking scores." This change was made to avoid implying that the selected docking-score ranges represent experimentally validated affinity thresholds.

3. Statements suggesting that phytochemicals "outperformed" or possessed "stronger binding" than approved EGFR inhibitors have been revised. The manuscript now states that certain phytochemicals received more favorable docking scores than the reference compounds under the same computational docking protocol, while explicitly emphasizing that this numerical difference does not demonstrate superior experimental binding affinity, inhibitory potency, or therapeutic efficacy.

4. The comparison with Osimertinib, Erlotinib, Afatinib, and Lazertinib has been retained as a computational reference comparison rather than as evidence of pharmacological superiority. We now explicitly state that similar or more favorable docking scores indicate only relative ranking within the applied computational framework and do not establish equivalent or superior biological activity.

5. The interpretation of compounds with intermediate and relatively less favorable docking scores has also been revised. We no longer infer pharmacological relevance solely from their docking-score ranges. Instead, we emphasize that docking results should be considered together with binding-mode plausibility, protein–ligand interaction patterns, physicochemical properties, ADMET characteristics, toxicity predictions, and molecular dynamics behavior.

6. A clarification has been added to the docking results and Table 3 indicating that docking scores should be interpreted as relative computational prioritization metrics and not as direct measures of experimental binding affinity or inhibitory potency.

7. The Discussion has been revised to explicitly acknowledge that differences in docking scores cannot establish the superiority of the phytochemicals over clinically used EGFR-targeting drugs. We now emphasize that experimental biochemical assays, including direct EGFR kinase inhibition measurements, are required to determine actual binding affinity and inhibitory potency.

These revisions have substantially moderated the interpretation of the docking results and aligned our conclusions with the limitations of computational docking. We now use the docking analysis primarily as an initial prioritization step within a broader integrated computational workflow rather than as standalone evidence of EGFR inhibitory activity or therapeutic efficacy. We appreciate the reviewer for highlighting this issue, which has helped us improve the scientific rigor and precision of the manuscript.

Comment 3: The use of only the wild-type EGFR structure (PDB: 1M17) does not adequately represent clinically important resistant NSCLC mutations such as L858R, T790M, and C797S, reducing the clinical r

Attachment Submitted filename: Response to the Editor and Reviewers.docx

# Editor report: Decision Letter 1

**DOI:** 10.1371/journal.pone.0357096.r003

**Authors:** Lalit Samant

12 Aug 2026

Computational Identification and Evaluation of Adenium obesum Phytochemicals as Potential EGFR Inhibitors for Targeted Therapy against Non-Small-Cell Lung Cancer

PONE-D-26-27333R1

Dear Dr. Kumer,

We’re pleased to inform you that your manuscript has been judged scientifically suitable for publication and will be formally accepted for publication once it meets all outstanding technical requirements.

Within one week, you’ll receive an e-mail detailing the required amendments. When these have been addressed, you’ll receive a formal acceptance letter and your manuscript will be scheduled for publication.

An invoice will be generated when your article is formally accepted. Please note, if your institution has a publishing partnership with PLOS and your article meets the relevant criteria, all or part of your publication costs will be covered. Please make sure your user information is up-to-date by logging into Editorial Manager at Editorial Manager® and clicking the ‘Update My Information' link at the top of the page. For questions related to billing, please contact billing support.

If your institution or institutions have a press office, please notify them about your upcoming paper to help maximize its impact. If they’ll be preparing press materials, please inform our press team as soon as possible -- no later than 48 hours after receiving the formal acceptance. Your manuscript will remain under strict press embargo until 2 pm Eastern Time on the date of publication. For more information, please contact onepress@plos.org.

Kind regards,

Lalit Samant

Academic Editor

PLOS One

Additional Editor Comments (optional):

Reviewers' comments:

# Editor report: Acceptance letter

**DOI:** 10.1371/journal.pone.0357096.r004

**Authors:** Lalit Samant

PONE-D-26-27333R1

PLOS One

Dear Dr. Kumer,

I'm pleased to inform you that your manuscript has been deemed suitable for publication in PLOS One. Congratulations! Your manuscript is now being handed over to our production team.

At this stage, our production department will prepare your paper for publication. This includes ensuring the following:

* All references, tables, and figures are properly cited

* All relevant supporting information is included in the manuscript submission,

* There are no issues that prevent the paper from being properly typeset

You will receive further instructions from the production team, including instructions on how to review your proof when it is ready. Please keep in mind that we are working through a large volume of accepted articles, so please give us a few days to review your paper and let you know the next and final steps.

Lastly, if your institution or institutions have a press office, please let them know about your upcoming paper now to help maximize its impact. If they'll be preparing press materials, please inform our press team within the next 48 hours. Your manuscript will remain under strict press embargo until 2 pm Eastern Time on the date of publication. For more information, please contact onepress@plos.org.

You will receive an invoice from PLOS for your publication fee after your manuscript has reached the completed accept phase. If you receive an email requesting payment before acceptance or for any other service, this may be a phishing scheme. Learn how to identify phishing emails and protect your accounts at https://explore.plos.org/phishing.

If we can help with anything else, please email us at customercare@plos.org.

Thank you for submitting your work to PLOS One and supporting open access.

Kind regards,

PLOS ONE Editorial Office Staff

on behalf of

Dr. Lalit Samant

Academic Editor

PLOS One
